# Hyperbolic $P(\Phi )_2$-model on the Plane

**DOI:** 10.1007/s00220-025-05486-0

**Published:** 2026-01-14

**Authors:** Tadahiro Oh, Leonardo Tolomeo, Yuzhao Wang, Guangqu Zheng

**Affiliations:** 1 School of Mathematics and Statistics, Beijing Institute of Technology, Beijing, 100081 China; 2 School of Mathematics, The University of Edinburgh, James Clerk Maxwell Building, The King’s Buildings, Peter Guthrie Tait Road, Edinburgh, UK; 3 The Maxwell Institute for the Mathematical Sciences, James Clerk Maxwell Building, The King’s Buildings, Peter Guthrie Tait Road, Edinburgh, UK; 4 School of Mathematics, Watson Building University of Birmingham, Edgbaston, Birmingham, B15 2TT UK; 5 Department of Mathematical Sciences, University of Liverpool, Mathematical Sciences Building, Liverpool, L69 7ZL UK; 6 Department of Mathematics and Statistics, Boston University, 665 Commonwealth Avenue, Boston, MA 02215 USA

## Abstract

In this paper, we construct invariant Gibbs dynamics for the hyperbolic $\Phi ^{k+1}_2$-model (namely, defocusing stochastic damped nonlinear wave equation forced by an additive space-time white noise) on the plane. (i) For this purpose, we first revisit the construction of a $\Phi ^{k+1}_2$-measure on the plane. More precisely, by establishing coming down from infinity for the associated stochastic nonlinear heat equation (SNLH) on the plane, we first construct a $\Phi ^{k+1}_2$-measure on the plane as a limit of the $\Phi ^{k+1}_2$-measures on large tori. (ii) We then construct invariant Gibbs dynamics for the hyperbolic $\Phi ^{k+1}_2$-model on the plane, by taking a limit of the invariant Gibbs dynamics on large tori constructed by the first two authors with Gubinelli and Koch (Int Math Res Not 21:16954–16999,2022). Here, our main strategy is to develop further the ideas from a recent work on the hyperbolic $\Phi ^3_3$-model on the three-dimensional torus by the first two authors and Okamoto (Mem Eur Math Soc 16, 2025), and to study convergence of the so-called enhanced Gibbs measures, for which coming down from infinity for the associated SNLH with positive regularity plays a crucial role. By combining wave and heat analysis together with ideas from optimal transport theory, we then conclude global well-posedness of the hyperbolic $\Phi ^{k+1}_2$-model on the plane and invariance of the associated Gibbs measure. As a byproduct of our argument, we also obtain invariance of the limiting $\Phi ^{k+1}_2$-measure on the plane under the dynamics of the parabolic $\Phi ^{k+1}_2$-model.

## Introduction

### Hyperbolic $\Phi ^{k+1}_2$-model

We study the following stochastic damped nonlinear wave equation (SdNLW) forced by an additive space-time white noise, posed on the plane ${\mathbb {R}}^2$:

1.1 $$\begin{aligned} \partial _t^2 u + \partial _tu + (1- \Delta )u \, + u^k = \sqrt{2}\xi , \end{aligned}$$

where $k \in 2{\mathbb {N}}+1$, *u* is a real-valued unknown, and $\xi (x, t)$ is a Gaussian space-time white noise on ${\mathbb {R}}^2 \times {\mathbb {R}}_+$ with the space-time covariance given by

$$\begin{aligned} {\mathbb {E}}\big [ \xi (x_1, t_1) \xi (x_2, t_2) \big ] = \delta (x_1 - x_2) \delta (t_1 - t_2). \end{aligned}$$

With $\vec {u} = (u, \partial _tu)$, define the energy ${\mathcal {E}}(\vec {u})$ by

1.2 $$\begin{aligned} \begin{aligned} {\mathcal {E}}(\vec {u})&= E(u) + \frac{1}{2} \int _{{\mathbb {R}}^2} (\partial _tu)^2 dx \\&= \frac{1}{2} \int _{{\mathbb {R}}^2} |\langle \nabla \rangle u|^2 dx + \frac{1}{2} \int _{{\mathbb {R}}^2} (\partial _tu)^2 dx + \frac{1}{k+1} \int _{{\mathbb {R}}^2} u^{k+1} dx, \end{aligned} \end{aligned}$$

where *E*(*u*) is given by

1.3 $$\begin{aligned} E(u) = \frac{1}{2} \int _{{\mathbb {R}}^2} |\langle \nabla \rangle u|^2 dx + \frac{1}{k+1} \int _{{\mathbb {R}}^2} u^{k+1} dx. \end{aligned}$$

Note that the energy ${\mathcal {E}}(\vec {u})$ is precisely the energy (= Hamiltonian) for the (deterministic) nonlinear wave equation (NLW):

1.4 $$\begin{aligned} \partial _t^2 u + (1 - \Delta ) u + u^k = 0. \end{aligned}$$

Namely, with $v = \partial _tu$, we can write NLW (1.4) in the following Hamiltonian formulation:1.5Similarly, we can write SdNLW (1.1) as1.6Consider a Gibbs measure $\vec {\rho }$ of the form

1.7 $$\begin{aligned} ``d\vec {\rho }(\vec {u} ) = {\mathcal {Z}}^{-1}e^{-{\mathcal {E}}(\vec {u} )}d\vec {u} = d\rho \otimes d\mu _0(\vec {u} )\text {''}, \end{aligned}$$

where $\vec {u} = (u, v) = (u, \partial _tu)$, $\rho$ is a $\Phi ^{k+1}_2$-measure on ${\mathbb {R}}^2$, and $\mu _0$ is the (spatial) white noise measure on ${\mathbb {R}}^2$. By drawing an analogy to the finite-dimensional Hamiltonian dynamics, we expect that the Gibbs measure $\vec {\rho }$ in (1.7) is invariant under the NLW dynamics (1.4) on ${\mathbb {R}}^2$. Moreover, it is easy to see that the white noise measure $\mu _0$ on the second component $v= \partial _tu$ is invariant under the Ornstein-Uhlenbeck dynamics:

1.8 $$\begin{aligned} \partial _tv = - v + \sqrt{2} \xi . \end{aligned}$$

Thus, by viewing the SdNLW dynamics (1.6) as a superposition of the NLW dynamics (1.5) and the Ornstein-Uhlenbeck dynamics (1.8), we expect the Gibbs measure $\vec {\rho }$ in (1.7) to be invariant under the SdNLW dynamics (1.1).

Indeed, from the stochastic quantization point of view [84, 86], the equation (1.1) corresponds to the so-called canonical stochastic quantization equation^1^ for the $\Phi ^{k+1}_2$-measure and thus is of importance in mathematical physics (in particular, constructive quantum field theory); see [86], where the terminology “canonical stochastic quantization” was introduced. In the parabolic setting, the stochastic quantization equation of the $\Phi ^{k+1}_d$-measure^2^ is given by the following parabolic $\Phi ^{k+1}_d$-model (= stochastic nonlinear heat equation (SNLH)):

1.9 $$\begin{aligned} \partial _tX + (1- \Delta )X \, + X^k = \sqrt{2}\xi , \end{aligned}$$

under which the $\Phi ^{k+1}_d$-measure remains invariant; see [1, 2, 24, 41, 42, 46, 47, 60, 61]. The equation (1.1) is the hyperbolic counterpart of the parabolic $\Phi ^{k+1}_d$-model when $d = 2$. For this reason, we refer to (1.1) as the *hyperbolic *$\Phi ^{k+1}_2$*-model*. Our main goal in this paper is to construct the global-in-time dynamics at the Gibbs equilibrium for the hyperbolic $\Phi ^{k+1}_2$-model (1.1) posed on ${\mathbb {R}}^2$.

In the parabolic case, Mourrat and Weber [60] proved pathwise global well-posedness of the parabolic $\Phi ^{k+1}_2$-model (1.9) on ${\mathbb {R}}^2$ with given deterministic initial data (of negative regularity) for any $k \in 2{\mathbb {N}}+1$ (see Remark 4 in [60]).^3^ When $k = 3$, the second author proved pathwise global well-posedness of the hyperbolic $\Phi ^{4}_2$-model on ${\mathbb {R}}^2$ with given deterministic initial data (but of positive regularity). However, such a pathwise global well-posedness result is not available for higher values of *k*. In order to overcome this difficulty, we apply Bourgain’s invariant measure argument [11, 12] ‘in spirit’ and construct global-in-time dynamics in a probabilistic manner.

For this purpose, we first revisit the construction of a $\Phi ^{k+1}_2$-measure on the plane. More precisely, by establishing coming down from infinity for the associated SNLH on the plane, we construct a $\Phi ^{k+1}_2$-measure on the plane as a limit of the $\Phi ^{k+1}_2$-measures on large tori. In order to construct invariant Gibbs dynamics for the hyperbolic $\Phi ^{k+1}_2$-model (1.1), we develop further the ideas from a recent work on the hyperbolic $\Phi ^3_3$-model [70] by the first two authors and Okamoto. More precisely, our main strategy is to study convergence of the so-called *enhanced Gibbs measures* (= distributions of the enhanced data sets), which provides the main statistical control, by combining wave and heat analysis together with ideas from optimal transport theory. In our analysis, we crucially exploit the finite speed of propagation for the hyperbolic $\Phi ^{k+1}_2$-model (1.1). Moreover, coming down from infinity for the associated SNLH with positive regularity plays a fundamental role, which is of independent interest. As a byproduct of our argument, we also obtain invariance of the limiting $\Phi ^{k+1}_2$-measure on the plane under the dynamics of the parabolic $\Phi ^{k+1}_2$-model; see Remarks 1.5 and 4.7.

### Review of the periodic problem

Following the previous works [43, 81], Gubinelli, Koch, and the first two authors [45] studied SdNLW (1.1) on the two-dimensional torus ${\mathbb {T}}^2= ({\mathbb {R}}/{\mathbb {Z}})^2$. By introducing a proper renormalization (see (1.25) below; see also [43, 81]), they constructed global-in-time invariant Gibbs dynamics for SdNLW (1.1) on ${\mathbb {T}}^2$ via the so-called Bourgain’s invariant measure argument [11, 12]. See [81] for the construction of invariant Gibbs dynamics for the deterministic NLW (1.4) on ${\mathbb {T}}^2$, preceding [45]. See also [76] for the corresponding results (for both SdNLW (1.1) and NLW (1.4)) on a two-dimensional compact Riemannian manifold without boundary.

Given $L > 0$, define a dilated torus ${\mathbb {T}}_L^2$ by setting

$$\begin{aligned} {\mathbb {T}}^2_L = ({\mathbb {R}}/L{\mathbb {Z}})^2. \end{aligned}$$

Then, the aforementioned result in [45] applies to SdNLW posed on ${\mathbb {T}}^2_L$ for any $L > 0$. Our main strategy for studying SdNLW (1.1) on ${\mathbb {R}}^2$ is then to take a large torus limit $L \rightarrow \infty$ of the *L*-periodic SdNLW dynamics on ${\mathbb {T}}^2_L$. As such, we first provide a review of the *L*-periodic problem on the dilated torus ${\mathbb {T}}^2_L$ in this subsection (especially since the presentation in [45] is only for the $L = 1$ case).

Let us first introduce some notations. Fix $L > 0$ and set

$$\begin{aligned} {\mathbb {Z}}_L^2 = ({\mathbb {Z}}/L)^2. \end{aligned}$$

Given $\lambda \in {\mathbb {Z}}_L^2$, we set

1.10 $$\begin{aligned} e_{\lambda }^L(x) = \frac{1}{L} e^{2\pi i \lambda \cdot x} \end{aligned}$$

for $x \in {\mathbb {T}}^2_L$. Then, $\{e_{\lambda }^L\}_{\lambda \in {\mathbb {Z}}_L^2}$ forms an orthonormal basis of $L^2({\mathbb {T}}^2_L)$. We define the Fourier transform $\widehat{f}(\lambda )$ of a function *f* on ${\mathbb {T}}^2_L$ by

$$\begin{aligned} \widehat{f}(\lambda ) = \int _{{\mathbb {T}}^2_L} f(x) \overline{e_\lambda ^L(x)} dx, \quad \lambda \in {\mathbb {Z}}^2_L, \end{aligned}$$

with the associated Fourier series expansion^4^:

$$\begin{aligned} f(x) = \sum _{\lambda \in {\mathbb {Z}}_L^2} \widehat{f}(\lambda ) e_\lambda ^L(x). \end{aligned}$$

We first go over the construction of the Gibbs measure on ${\mathbb {T}}^2_L$; see [25, 80] for details (with $L = 1$). See also [54] for the construction of analogous log-correlated Gibbs measures in the one-dimensional setting. Given $s \in {\mathbb {R}}$, let $\mu _{s, L}$ denote a Gaussian measure on *L*-periodic distributions with the covariance operator $(1-\Delta _{{\mathbb {T}}^2_L})^{-s}$, formally defined by^5^

1.11 $$\begin{aligned} \begin{aligned} d \mu _{s, L} (u)&= {\mathcal {Z}}_{s, L}^{-1} \exp \bigg (-\frac{1}{2} \Vert u\Vert _{{H}^s({\mathbb {T}}^2_L)}^2\bigg ) du\\&= {\mathcal {Z}}_{s, L}^{-1} \prod _{\lambda \in {\mathbb {Z}}_L^2} e^{-\frac{1}{2} \langle \lambda \rangle ^{2s} |\widehat{u}(\lambda )|^2} d\widehat{u}(\lambda ) , \end{aligned} \end{aligned}$$

where $\langle \,\cdot \, \rangle = (1 + |\cdot |^2)^\frac{1}{2}$ and $\widehat{u}(\lambda )$, $\lambda \in {\mathbb {Z}}^2_L$, denotes the Fourier transform of *u* on ${\mathbb {T}}^2_L$. We note that $\mu _{1, L}$ corresponds to the massive Gaussian free field on ${\mathbb {T}}^2_L$, while $\mu _{0, L}$ corresponds to the white noise on ${\mathbb {T}}^2_L$. We then set

1.12 $$\begin{aligned} \vec {\mu }_L = \mu _{L} \otimes \mu _{0, L}, \quad \text {where} \quad \mu _{L} = \mu _{1, L}. \end{aligned}$$

Given $$s \in {\mathbb {R}}$$, let

$$\begin{aligned} \vec {H}^s({\mathbb {T}}^2_L) = H^s({\mathbb {T}}^2_L) \times H^{s-1}({\mathbb {T}}^2_L). \end{aligned}$$

Then, $\vec {\mu }_L$ in (1.12) is formally given by

1.13 $$\begin{aligned} d \vec {\mu }_{L} (u, v)&= {\mathcal {Z}}_{L}^{-1} \exp \bigg (-\frac{1}{2} \Vert (u, v) \Vert _{ \vec {H}^1({\mathbb {T}}^2_L)}^2\bigg ) du dv. \end{aligned}$$

Namely, the measure $\vec {\mu }_L$ is defined as the induced probability measure under the map:

$$\begin{aligned} \omega \in \Omega \longmapsto \big (u(\omega ), v(\omega )\big ) \in {\mathcal {D}}'({\mathbb {T}}_L^2)\times {\mathcal {D}}'({\mathbb {T}}_L^2) \subset {\mathcal {D}}'({\mathbb {R}}^2)\times {\mathcal {D}}'({\mathbb {R}}^2), \end{aligned}$$

where $u(\omega ) = u_L(\omega )$ and $v(\omega ) = v_L(\omega )$ are given by the following Gaussian Fourier series:

1.14 $$\begin{aligned}  &   u(\omega ) = u_L(\omega ) = \sum _{\lambda \in {\mathbb {Z}}^2_L} \frac{g_{L\lambda }(\omega )}{\langle \lambda \rangle }e_\lambda ^L \quad \text {and}\nonumber \\  &   v(\omega )= v_L(\omega ) = \sum _{\lambda \in {\mathbb {Z}}^2_L} h_{L\lambda }(\omega )e_\lambda ^L. \end{aligned}$$

Here, $\{g_n,h_n\}_{n\in {\mathbb {Z}}^2}$ denotes a family of independent standard complex-valued Gaussian random variables conditioned that $g_{-n} = \overline{g_n}$ and $h_{-n} = \overline{h_n}$, $n \in {\mathbb {Z}}^2$. From (1.14), it is easy to see that $\vec {\mu }_L = \mu _{1, L}\otimes \mu _{0, L}$ is supported on $\vec {H}^{s}({\mathbb {T}}^2_L) {\setminus } \vec {H}^0({\mathbb {T}}^2_L)$ for $s < 0$.

In view of (1.2) and (1.13), the Gibbs measure $\vec {\rho }_L$ on ${\mathbb {T}}^2_L$ is formally given by

1.15 $$\begin{aligned} d\vec {\rho }_L(u,\partial _tu ) = {\mathcal {Z}}_L^{-1} e^{ - \frac{1}{k+1} \int _{{\mathbb {T}}^2_L} u^{k+1} dx} d\vec {\mu }_L (u, \partial _tu ). \end{aligned}$$

Due to the roughness of the support of $\vec {\mu }_L$, the interaction potential $\int _{{\mathbb {T}}^2_L} u^{k+1} dx$ in (1.15) is not well defined and thus a renormalization is required to give a proper meaning to the expression in (1.15).

Given $N \in {\mathbb {N}}$, let ${\textbf{P}}_N$ be the Dirichlet projection onto the frequencies $\{|\lambda | \le N\}$. Then, with *u* as in (1.14), it follows from (1.14), (1.10), and a Riemann sum approximation that

1.16 $$\begin{aligned} \begin{aligned} \sigma _{N, L} :\!&= {\mathbb {E}}\big [\big ({\textbf{P}}_{N}u(x)\big )^2\big ] = \sum _{\begin{array}{c} \lambda \in {\mathbb {Z}}_L^2\\ |\lambda |\le N \end{array}}\frac{1}{\langle \lambda \rangle ^2}\frac{1}{L^2} \\&\hspace{0.5mm} = \sum _{\begin{array}{c} n\in {\mathbb {Z}}^2\\ |n|\le L N \end{array}}\frac{1}{\langle \frac{n}{L} \rangle ^2}\frac{1}{L^2} \sim \int _{{\mathbb {R}}^2} {\textbf{1}}_{|z|\le N} \frac{dz}{1 + |z|^2} \sim \log N \end{aligned} \end{aligned}$$

for $N \gg 1$, independent of $x\in {\mathbb {T}}^2_L$, which diverges to $\infty$ as $N\rightarrow \infty$ (for each fixed period $L > 0$). In particular, $u = \lim _{N \rightarrow \infty } {\textbf{P}}_N u$ is only a distribution and thus, for any integer $\ell \ge 2$, the power $({\textbf{P}}_N u)^\ell$ does not converge to any limit. For each $x \in {\mathbb {T}}^2_L$, we now define the Wick power $:\!({\textbf{P}}_{N} u)^{\ell }(x) \!: \, = \, :\!({\textbf{P}}_{N} u)^{\ell }(x) \!:_L$ by^6^

1.17 $$\begin{aligned} :\!({\textbf{P}}_{N} u)^{\ell }(x) \!: \, = H_{\ell } ({\textbf{P}}_{N} u(x); \sigma _{N, L}), \end{aligned}$$

where $H_{\ell }(x;\sigma )$ is the Hermite polynomial of degree $\ell \in {\mathbb {Z}}_{\ge 0}:= {\mathbb {N}}\cup \{0\}$ with a variance parameter $\sigma > 0$. Arguing as in [43–45], we can show that $:\!({\textbf{P}}_{N} u)^{\ell } \!:$ converges, almost surely and in $L^p(\Omega )$ for any finite $p \ge 1$, to a limit, denoted by $:\! u^{\ell } \!:$ in $H^s({\mathbb {T}}^2_L)$, $s < 0$. Then, by defining the truncated renormalized potential energy:

1.18 $$\begin{aligned} R_N^L(u) = \frac{1}{k+1} \int _{{\mathbb {T}}^2_L} :\!({\textbf{P}}_N u)^{k+1}(x) \!: dx, \end{aligned}$$

a standard computation shows that $\{R_N^L (u) \}_{N \in {\mathbb {N}}}$ converges to some limit, denoted by $R^L(u)$, in $L^p(d\mu _L)$ for any finite $p \ge 1$, as $N \rightarrow \infty$. The convergence of the truncated renormalized potential energy $\{R_N^L (u) \}_{N \in {\mathbb {N}}}$ together with Nelson’s estimate implies that the truncated density $\{e^{-R^L_N(u)}\}_{N\in {\mathbb {N}}}$ converges to the limiting density $e^{-R^L(u)}$ in $L^p(d\mu _L)$ for any finite $p \ge 1$, as $N \rightarrow \infty$. Hence, by defining the renormalized truncated Gibbs measure:

1.19 $$\begin{aligned} d\vec {\rho }_{N, L}(u,\partial _tu )= {\mathcal {Z}}_{N, L}^{-1}e^{-R_N^L(u)}d\vec {\mu }_L(u,\partial _tu), \end{aligned}$$

we then conclude that the renormalized truncated Gibbs measure $\vec {\rho }_{N, L}$ converges in total variation to the limiting Gibbs measure $\vec {\rho }_L$ given by

1.20 $$\begin{aligned} \begin{aligned} d\vec {\rho }_L(u,\partial _tu )&= {\mathcal {Z}}_L^{-1} e^{- R^L(u)}d\vec {\mu }_L(u, \partial _tu )\\&= {\mathcal {Z}}_L^{-1} \exp \bigg ( - \frac{1}{k+1} \int _{{\mathbb {T}}^2_L} :\! u^{k+1}(x) \!: dx\bigg )d\vec {\mu }_L(u, \partial _tu ). \end{aligned} \end{aligned}$$

Furthermore, for each fixed $0< L < \infty$, the resulting Gibbs measure $\vec {\rho }_L$ is equivalent^7^ to the base Gaussian measure $\vec {\mu }_L$.

#### Remark 1.1

The first marginal of the Gibbs measure $\vec {\rho }_L$ (namely, after integrating (1.20) in $\partial _tu$) is precisely the $\Phi ^{k+1}_2$-measure $\rho _L$ on ${\mathbb {T}}^2_L$, given by

1.21 $$\begin{aligned} \begin{aligned} d\rho _L(u)&= {\mathcal {Z}}_L^{-1} e^{- R^L(u)}d\mu _L(u)\\&= {\mathcal {Z}}_L^{-1} \exp \bigg ( - \frac{1}{k+1} \int _{{\mathbb {T}}^2_L} :\! u^{k+1}(x) \!: dx\bigg )d\mu _L(u ), \end{aligned} \end{aligned}$$

where $\mu _{L} = \mu _{1, L}$ is as in (1.12). Note that $\rho _L$ is the Gibbs measure associated with the energy functional *E*(*u*) in (1.3). The Gibbs measure $\vec {\rho }_L$ in (1.20) can then be written as $\vec {\rho }_L = \rho _L \otimes \mu _{0, L}$, where $\mu _{0, L}$ is the (spatial) white noise measure on ${\mathbb {T}}^2_L$.

Next, we discuss stochastic dynamics associated with the Gibbs measure $\vec {\rho }_L$ in (1.20). This process is known as stochastic quantization [84]. In the parabolic setting, Da Prato and Debussche [24] studied the parabolic $\Phi ^{k+1}_2$-model (1.9) on ${\mathbb {T}}^2$, associated with the $\Phi ^{k+1}_2$-measure $\rho _{L = 1}$ in (1.21). With the Wick renormalization, they constructed global-in-time invariant dynamics for (1.9) on ${\mathbb {T}}^2$ with the initial data distributed by the $\Phi ^{k+1}_2$-measure $\rho _{L = 1}$. This result is readily applicable to the parabolic $\Phi ^{k+1}_2$-model (1.9) posed on the dilated torus ${\mathbb {T}}^2_L$ for any $L > 0$. In [60], with an intricate use of weighted Besov spaces (see (2.25) below), Mourrat and Weber extended this result to the parabolic $\Phi ^{k+1}_2$-model on the plane ${\mathbb {R}}^2$.

We now consider the hyperbolic $\Phi ^{k+1}_2$-model on ${\mathbb {T}}^2_L$ for fixed $L > 0$:

1.22 $$\begin{aligned} \partial _t^2 u + \partial _tu + (1- \Delta )u \, + u^k = \sqrt{2}\xi _L \end{aligned}$$

with the Gibbsian initial data distributed by the Gibbs measure $\vec {\rho }_L$ in (1.20), where $\xi _L$ is a space-time white noise on ${\mathbb {T}}^2_L\times {\mathbb {R}}_+$. In view of the equivalence of the Gibbs measure $\vec {\rho }_L$ in (1.20) and the base Gaussian measure $\vec {\mu }_L$ in (1.12) for each fixed $L > 0$, it suffices to study (1.22) with the Gaussian initial data distributed by $\vec {\mu }_L$.

Let $\Phi = \Phi _L$ be the solution to the linear stochastic damped wave equation on ${\mathbb {T}}^2_L$ with the Gaussian initial data distributed by $\vec {\mu }_L$ in (1.12):

$$\begin{aligned} {\left\{ \begin{array}{ll} \partial _t^2 \Phi + \partial _t\Phi +(1-\Delta )\Phi = \sqrt{2}\xi _L\\ (\Phi ,\partial _t\Phi )|_{t=0}=(\phi _0,\phi _1) \quad \text {with }\ {{\,\textrm{Law}\,}}(\phi _0, \phi _1) = \vec {\mu }_L. \end{array}\right. } \end{aligned}$$

Here, ${{\,\textrm{Law}\,}}(X)$ of a random variable *X* denotes the law of *X*. Define the linear damped wave propagator ${\mathcal {D}}(t)$ by

1.23 $$\begin{aligned} {\mathcal {D}}(t) = e^{-\frac{t}{2}}\frac{\sin \Big (t\sqrt{\frac{3}{4}-\Delta }\Big )}{\sqrt{\frac{3}{4}-\Delta }} \end{aligned}$$

as a Fourier multiplier operator. Then, the stochastic convolution $\Phi$ defined above can be expressed as

$$\begin{aligned} \Phi (t) = \big (\partial _t{\mathcal {D}}(t) +{\mathcal {D}}(t)\big )\phi _0 + {\mathcal {D}}(t)\phi _1+ \sqrt{2}\int _0^t{\mathcal {D}}(t - t')dW_L(t'), \end{aligned}$$

where $W_L$ denotes a cylindrical Wiener process on $L^2({\mathbb {T}}^2_L)$:

1.24 $$\begin{aligned} W_L(t) = \sum _{\lambda \in {\mathbb {Z}}_L^2 } B_{\lambda } (t) e_\lambda ^L \end{aligned}$$

and $\{ B_\lambda \}_{\lambda \in {\mathbb {Z}}^2_L}$ is defined by $B_{\lambda }(t) = \langle \xi _L, {\textbf{1}}_{[0, t]} \cdot e_\lambda ^L \rangle _{{\mathbb {T}}^2_L\times {\mathbb {R}}_+}$. Here, $\langle \cdot , \cdot \rangle _{{\mathbb {T}}^2_L\times {\mathbb {R}}_+}$ denotes the duality pairing on ${\mathbb {T}}^2_L\times {\mathbb {R}}_+$. Then, we see that $\{ B_\lambda \}_{\lambda \in {\mathbb {Z}}^2_L}$ is a family of mutually independent complex-valued^8^ Brownian motions conditioned that $B_{-\lambda } = \overline{B_\lambda }$, $\lambda \in {\mathbb {Z}}^2_L$. Note that $\text {Var}(B_\lambda (t)) = t$, $\lambda \in {\mathbb {Z}}^2_L$.

Let $N \in {\mathbb {N}}$. Given $(x, t) \in {\mathbb {T}}^2_L \times {\mathbb {R}}_+$, we see that $\Phi _N(x, t)={\textbf{P}}_N\Phi (x, t)$ is a mean-zero real-valued Gaussian random variable with variance

$$\begin{aligned} {\mathbb {E}}\big [\Phi _N^2(x, t)\big ] = \sigma _{N, L} \sim \log N \longrightarrow \infty \end{aligned}$$

as $N \rightarrow \infty$, where $\sigma _{N, L}$ is as in (1.16). As in (1.17), we define the Wick power $:\!\Phi _N^{\ell }(x, t) \!:$ by setting

1.25 $$\begin{aligned} :\!\Phi _N^{\ell }(x, t) \!: \, = H_{\ell } (\Phi _N(x, t); \sigma _{N, L}). \end{aligned}$$

Then, $:\!\Phi _N^{\ell } \!:$ converges, almost surely and in $L^p(\Omega )$ for any finite $p \ge 1$, to a limit, denoted by $:\!\Phi ^{\ell } \!:$ , in $C({\mathbb {R}}_+; H^s({\mathbb {T}}^2_L))$, $s < 0$.^9^

Given $N \in {\mathbb {N}}$, consider the following truncated SdNLW on ${\mathbb {T}}^2_L$:

1.26 $$\begin{aligned} \partial _t^2 u_N + \partial _tu_N +(1-\Delta ) u_N + {\textbf{P}}_N\big (({\textbf{P}}_N u_N)^{k} \big ) = \sqrt{2} \xi _L . \end{aligned}$$

Proceeding with the first order expansion ( [12, 24, 55]):

1.27 $$\begin{aligned} u_N = \Phi _N + v_N, \end{aligned}$$

we see that the remainder term $v_N = u_N - \Phi _N$ satisfies

1.28 $$\begin{aligned} \partial _t^2 v_N + \partial _tv_N + (1 - \Delta ) v_N + \sum _{\ell =0}^k {k\atopwithdelims ()\ell } {\textbf{P}}_N \big (\Phi _N^\ell v_N^{k-\ell }\big ) = 0 \end{aligned}$$

with the zero initial data. As pointed out above, the power $\Phi _N^\ell$ does not converge to any limit as $N\rightarrow \infty$. Furthermore, a triviality is known for (1.28) (at least for $k = 3$). Namely, the solution $u_N$ to (1.26) tends to 0 as we remove the regularization ($N \rightarrow \infty$); see [68]. This triviality result necessitates the use of a renormalization. Therefore, we instead consider the following renormalized version of (1.28):

1.29 $$\begin{aligned} \partial _t^2 v_N + \partial _tv_N + (1 - \Delta ) v_N + \sum _{\ell =0}^k {k\atopwithdelims ()\ell } {\textbf{P}}_N \big (:\!\Phi _N^\ell \!: v_N^{k-\ell }\big ) = 0 \end{aligned}$$

with the zero initial data. By formally taking a limit as $N \rightarrow \infty$, we then obtain the limiting equation:

1.30 $$\begin{aligned} \partial _t^2 v +\partial _tv+ (1 - \Delta ) v + \sum _{\ell =0}^k {k\atopwithdelims ()\ell }:\!\Phi ^\ell \!: v^{k-\ell } = 0. \end{aligned}$$

Given the almost sure space-time regularity of the Wick powers $\{:\!\Phi ^\ell \!: \,\}_{\ell = 1}^k$, standard deterministic analysis with the product estimates (Lemma 2.8) and Sobolev’s inequality yields local well-posedness of (1.30) with the continuous map, sending the enhanced data set to the solution:

$$\begin{aligned} (\Phi , :\!\Phi ^2\!:, \dots , :\!\Phi ^k\!:\,)&\in \big (C([0, T]; H^{-\varepsilon }({\mathbb {T}}^2_L))\big )^{\otimes k} \\&\longmapsto (v, \partial _tv) \in C([0, T]; \vec {H}^{1-\varepsilon }({\mathbb {T}}^2_L)) \end{aligned}$$

for some small $\varepsilon > 0$. This deterministic local well-posedness analysis also applies to (1.29), uniformly in $N \in {\mathbb {N}}$.

In view of the decomposition (1.27), the renormalized truncated SdNLW (1.29) for $v_N$ corresponds to the following the renormalized truncated SdNLW for $u_N = \Phi _N + v_N$:

1.31 $$\begin{aligned} \partial _t^2 u_N +\partial _tu_N + (1 - \Delta ) u_N + {\textbf{P}}_N \big (:\! ({\textbf{P}}_N u_N)^k\!: \big ) = \sqrt{2}\xi _L. \end{aligned}$$

Here, the renormalized nonlinearity in (1.31) is interpreted as

$$\begin{aligned} {\textbf{P}}_N \big (:\! ({\textbf{P}}_N u_N)^k\!: \big )&= {\textbf{P}}_N \big (:\! (\Phi _N + {\textbf{P}}_N v_N)^k\!: \big )\\&= \sum _{\ell =0}^k {k\atopwithdelims ()\ell } {\textbf{P}}_N \big (:\!\Phi _N^\ell \!: v_N^{k-\ell }\big ). \end{aligned}$$

Then, the aforementioned local well-posedness of (1.30), together with the convergence of the truncated enhanced data set $\{:\!\Phi _N^\ell \!: \,\}_{\ell = 1}^k$ to the limiting enhanced data set $\{:\!\Phi ^\ell \!: \,\}_{\ell = 1}^k$, implies that $u_N$ converges almost surely to a stochastic process $u = \Phi + v$, where *v* satisfies (1.30). It is in this sense that we say that the renormalized SdNLW on the dilated torus ${\mathbb {T}}^2_L$:

1.32 $$\begin{aligned} \partial _t^2 u + \partial _tu + (1 - \Delta ) u + :\!u^k\!: \,= \sqrt{2} \xi _L \end{aligned}$$

is locally well-posed with the Gaussian initial data distributed by $\vec {\mu }_L$, and hence with the Gibbsian initial data in view of the equivalence of the Gibbs measure $\vec {\rho }_L$ and the base Gaussian measure $\vec {\mu }_L$.

Once the local-in-time dynamics is constructed, Bourgain’s invariant measure argument [11, 12] allows us to construct global-in-time dynamics for the hyperbolic $\Phi ^{k+1}_2$-model (1.32) on ${\mathbb {T}}^2_L$ and to prove invariance of the Gibbs measure $\vec {\rho }_L$ in (1.20). This argument is based essentially on the following three ingredients:

- invariance of the truncated Gibbs measure $\vec {\rho }_{N, L}$ in (1.19) under the truncated SdNLW dynamics (1.31), which provides a probabilistic growth bound on the solution $u_N$, uniformly in $N \in {\mathbb {N}}$,
- a PDE approximation argument (analogous to the local well-posedness argument in the current setting),
- convergence (in total variation) of the truncated Gibbs measure $\vec {\rho }_{N, L}$ in (1.19) to the limiting Gibbs measure $\vec {\rho }_L$ in (1.20).

See [76] for full details of this argument.

### Main result

Our goal in this paper is to extend the construction of the invariant Gibbs dynamics of the hyperbolic $\Phi ^{k+1}_2$-model on ${\mathbb {T}}^2_L$ described in the previous subsection to the plane ${\mathbb {R}}^2$. The main idea is to apply Bourgain’s invariant measure argument ‘in spirit’, where we replace the frequency truncation parameter $N \rightarrow \infty$ by the growing period $L \rightarrow \infty$.

We first state our main result. Given $s \in {\mathbb {R}}$, $1 \le p < \infty$, and $\mu > 0$, let $W^{s, p}_\mu ({\mathbb {R}}^2)$ be the weighted Sobolev space defined in (2.22) below. We set

$$\begin{aligned} \vec {W}^{s, p}_\mu ({\mathbb {R}}^2) = W^{s, p}_\mu ({\mathbb {R}}^2) \times W^{s-1, p}_\mu ({\mathbb {R}}^2). \end{aligned}$$

We also set

$$\begin{aligned} \vec {H}_\text {loc}^{s}({\mathbb {R}}^2) = H_\text {loc}^{s}({\mathbb {R}}^2) \times H_\text {loc}^{s-1}({\mathbb {R}}^2). \end{aligned}$$

Here, $H_\text {loc}^{s}({\mathbb {R}}^2)$ denotes the class of functions *u* belonging to $H^s(K)$ for each compact subset $K \subset {\mathbb {R}}^2$, where the $H^s(K)$-norm is defined as the restriction norm onto the set *K*; see (2.3) below. The topology on $H_\text {loc}^{s}({\mathbb {R}}^2)$ is induced by the following metric:

$$\begin{aligned} d_{H_\text {loc}^{s}}(f, g) = \sum _{j=1}^\infty 2^{-j} \frac{ \Vert f - g\Vert _{H^{s}([-j, j]^2)} }{1 + \Vert f - g\Vert _{ H^{s}([-j, j]^2) } }. \end{aligned}$$

By definition, we have $d_{H_\text {loc}^{s}}(f_n, f)\rightarrow 0$ if and only if $f_n$ converges to *f* in $H^{s}([-j, j]^2)$ for each $j\in {\mathbb {N}}$. In the following, we endow $C({\mathbb {R}}_+; \vec {H}_loc ^{s}({\mathbb {R}}^2))$ with the compact-open topology in time.^10^

Throughout the paper, we assume that random initial data are independent of a stochastic forcing (such as $\xi _L$ and $\xi$). Given $R> 0$, let $B_R = \{ x \in {\mathbb {R}}^2: |x|\le R\}$ denotes the (closed) ball of radius *R* centered at the origin and ${\textbf{C}}_R$ denote the cone given by

1.33 $$\begin{aligned} \begin{aligned} {\textbf{C}}_R&= \{ (x, t)\in {\mathbb {R}}^2\times {\mathbb {R}}_+: |x| + |t| \le R \} \\&= \{ (x, t)\in {\mathbb {R}}^2\times [0, R]: x \in B_{R-t} \} . \end{aligned} \end{aligned}$$

#### Theorem 1.2

Let $k \in 2{\mathbb {N}}+1$. There exists a subset ${\mathcal {A}}= \{ L_j: j \in {\mathbb {N}}\} \subset {\mathbb {N}}$ (with $L_j < L_{j'}$ for $j < j'$) such that the following statements hold.

(i) Let $s < 0$, finite $p \ge 1$, and $\mu > 0$. Let $\vec {\rho }_L$ be the Gibbs measure on the dilated torus ${\mathbb {T}}^2_{L}$ defined in (1.20). When viewed as a probability measure on the weighted Sobolev space $\vec {W}^{s, p}_\mu ({\mathbb {R}}^2)$, the $L_j$-periodic Gibbs measure $\vec {\rho }_{L_j}$ converges weakly to a limiting Gibbs measure $\vec {\rho }_\infty$ as $j \rightarrow \infty$. The limiting Gibbs measure $\vec {\rho }_\infty$ on ${\mathbb {R}}^2$ can be written as

$$\begin{aligned} \vec {\rho }_\infty = \rho _\infty \otimes \mu _{0, \infty }, \end{aligned}$$

where $\rho _\infty$ is the $\Phi ^{k+1}_2$-measure on ${\mathbb {R}}^2$ constructed as a limit of the $L_j$-periodic $\Phi ^{k+1}_2$-measure $\rho _{L_j}$, and $\mu _{0, \infty }$ is the white noise measure on ${\mathbb {R}}^2$.

(ii) The hyperbolic $\Phi ^{k+1}_2$-model on ${\mathbb {R}}^2$:

1.34 $$\begin{aligned} \partial _t^2 u + \partial _tu + (1 - \Delta ) u \, + :\!u^k\!: \,= \sqrt{2} \xi \end{aligned}$$

is globally well-posed almost surely with respect to the Gibbsian initial data distributed by the Gibbs measure $\vec {\rho }_\infty$ on ${\mathbb {R}}^2$, constructed in Part (i). Furthermore, the Gibbs measure $\vec {\rho }_\infty$ is invariant under the resulting hyperbolic $\Phi ^{k+1}_2$-dynamics on ${\mathbb {R}}^2$.

More precisely, the following statements hold. There exist a sequence $\{(u_{L_j}, \partial _tu_{L_j})\}_{j \in {\mathbb {N}}}$ and a non-trivial stochastic process $(u, \partial _tu)$ almost surely belonging to $C({\mathbb {R}}_+; \vec {H}_loc ^{-\varepsilon }({\mathbb {R}}^2))$ for any $\varepsilon >0$ such that

- for each $j \in {\mathbb {N}}$, $(u_{L_j}, \partial _tu_{L_j})$ is the global-in-time solution to SdNLW (1.32) on ${\mathbb {T}}^2_{L_j}$ with ${{\,\textrm{Law}\,}}\big ((u_{L_j}(0), \partial _tu_{L_j}(0))\big ) = \vec {\rho }_{L_j}$, constructed in [45],
- as $j \rightarrow \infty$, $(u_{L_j}(0), \partial _tu_{L_j}(0))$ converges almost surely to $(u(0), \partial _tu(0))$, distributed by the limiting Gibbs measure $\vec {\rho }_\infty$, in $\vec {H}_loc ^{-\varepsilon }({\mathbb {R}}^2)$,
- given any $R>0$, $(u_{L_j}, \partial _tu_{L_j})$ converges in probability to $(u, \partial _tu)$ on the cone ${\textbf{C}}_R$ (more precisely, in $L^\infty ([0, R]; \vec {H}^{-\varepsilon }(B_{R-t})); ~see (2.4))$), as $j \rightarrow \infty$.

Furthermore, the law of $(u(t), \partial _tu(t))$ for any $t \in {\mathbb {R}}_+$ is given by the renormalized Gibbs measure $\vec {\rho }_\infty$.

In Theorem 1.2 (i), we needed to take a sequence $\{L_j\}_{j \in {\mathcal {A}}}$ due to the non-uniqueness of the limiting Gibbs measure on ${\mathbb {R}}^2$; see Remark 1.4 (ii).

As in the periodic case, the limit $(u, \partial _tu)$ constructed in Theorem 1.2 (ii) is a solution to the hyperbolic $\Phi ^{k+1}_2$-model (1.34) on ${\mathbb {R}}^2$ with the Gibbsian initial data in the sense that the remainder $v = u - \Phi$ satisfies the equation (1.30) on ${\mathbb {R}}^2\times {\mathbb {R}}_+$, where $\Phi$ denotes the solution to the linear stochastic damped wave equation on ${\mathbb {R}}^2$ with the Gibbsian initial data:

$$\begin{aligned} {\left\{ \begin{array}{ll} \partial _t^2 \Phi + \partial _t\Phi +(1-\Delta )\Phi = \sqrt{2}\xi \\ (\Phi ,\partial _t\Phi )|_{t=0}=(u_0, u_1) \quad \text {with }\ {{\,\textrm{Law}\,}}(u_0, u_1) = \vec {\rho }_\infty . \end{array}\right. } \end{aligned}$$

We note that the solution $(v, \partial _tv)$ to (1.30) thus constructed is unique in $C({\mathbb {R}}_+; \vec {H}^{1-\varepsilon }_\text {loc}({\mathbb {R}}^2))$, which follows from the finite speed of propagation and a local well-posedness result presented in Sect. 4.1. Namely, the solution $(u, \partial _tu)$ to the hyperbolic $\Phi ^{k+1}_2$-model (1.34) on ${\mathbb {R}}^2$ with the Gibbsian initial data, constructed in Theorem 1.2 (ii), is unique in the class

$$\begin{aligned} (\Phi , \partial _t\Phi ) + C({\mathbb {R}}_+; \vec {H}^{1-\varepsilon }_\text {loc}({\mathbb {R}}^2)). \end{aligned}$$

As for the construction of the limiting $\Phi ^{k+1}_2$-measure $\rho _\infty$ on ${\mathbb {R}}^2$, see also [3, 88] and the references therein.^11^ Note that the assumption on the almost sure convergence of $(u_{L_j}(0), \partial _tu_{L_j}(0))$ in Theorem 1.2 (ii) indeed follows from the weak convergence of $\vec {\rho }_{L_j}$ to $\vec {\rho }_\infty$ in Theorem 1.2 (i) and the Skorokhod representation theorem (Lemma 2.19) and therefore is not an additional assumption.

Stochastic nonlinear wave equations (SNLW) have attracted extensive attention from both applied and theoretical points of view; see [26, Chapter 13] and [66] for the references therein. In particular, over the last five years, we have seen a significant progress in the well-posedness theory of SNLW in the singular setting^12^:

$$\begin{aligned} \partial _t^2 u + \partial _tu + (1 - \Delta ) u + {\mathcal {N}}(u) = \zeta , \end{aligned}$$

where the noise $\zeta$ is primarily taken to be a space-time white noise $\xi$. Here, ${\mathcal {N}}(u)$ denotes a nonlinearity which may be of a power-type [16, 18, 43–45, 68–70, 76, 82, 93] and trigonometric and exponential nonlinearities [40, 74, 75, 77, 99]. We also mention the works [67, 71, 72, 81, 83, 89] on the (deterministic) nonlinear wave equations (1.4) with rough random initial data and [28, 29, 66] on SNLW with more singular (both in space and time) noises. We point out that the only known well-posedness result up to date for singular SNLW posed on an unbounded domain is the work [93] by the second author, where he established *pathwise* global well-posedness of the cubic SNLW on ${\mathbb {R}}^2$ with an additive space-time white noise forcing:

1.35 $$\begin{aligned} \partial _t^2 u + (1- \Delta )u \, + u^k = \xi , \end{aligned}$$

where $k = 3$. Note that a slight modification of the argument yields pathwise global well-posedness for SdNLW (1.1) on ${\mathbb {R}}^2$ when $k = 3$.

Theorem 1.2 provides the second well-posedness result for singular SNLW on an unbounded domain. Our construction of the global-in-time dynamics, however, is quite different from that in [93]. In particular, it is not pathwise, but is based on a probabilistic argument, more precisely, on Bourgain’s invariant measure argument ‘in spirit’. Here, by pathwise global well-posedness, we mean global well-posedness for any *deterministic* initial data in a given function space, where only a priori control of explicitly given stochastic terms is used (namely the statistical information of a solution is not used), whereas Bourgain’s invariant measure argument crucially relies on the statistical information of solutions (to approximating equations). We point out that pathwise global well-posedness (in the sense described above) of SdNLW (1.1) or SNLW (1.35) for the (super-)quintic case $k \ge 5$ remains a challenging open question even on the torus ${\mathbb {T}}^2$,^13^ and therefore, the situation for the stochastic wave equation is completely different from SNLH (1.9) on ${\mathbb {R}}^2$, where Mourrat and Weber [60] proved pathwise global well-posedness of (1.9) on ${\mathbb {R}}^2$ for any $k \in 2{\mathbb {N}}+ 1$.

Thanks to the finite speed of propagation, the argument in [43, 45] yields local well-posedness of (1.34) on the ball $B_R \subset {\mathbb {R}}^2$ for each $R > 0$; see Proposition 4.2 below. As $R \rightarrow \infty$, however, the local existence time shrinks to 0. In order to construct a solution to (1.34) on some time interval $[0, \tau ]$, uniformly on ${\mathbb {R}}^2$, we need to make use of statistical ingredients, and thus, establishing local well-posedness of (1.34), uniformly on ${\mathbb {R}}^2$, is essentially as difficult as establishing its global well-posedness, as already observed in [93].

On the dilated torus ${\mathbb {T}}^2_L$, the Gibbs measure $\vec {\rho }_L$ and the base Gaussian measure $\vec {\mu }_L$ are equivalent for each finite $L > 0$. However, this equivalence of $\vec {\rho }_L$ and $\vec {\mu }_L$ is not uniform when $L \rightarrow \infty$, since the potential energy $R^L$, defined as the limit of $R^L_N$ in (1.18), grows like $\sim L$ as $L \rightarrow \infty$. Indeed from (1.18), (1.17), and Lemma 2.13 with (1.10), we have

$$\begin{aligned} {\mathbb {E}}_{\vec {\mu }_L}&\Big [\big (R^L(u)\big )^2\Big ] = \lim _{N \rightarrow \infty } {\mathbb {E}}_{\vec {\mu }_L} \Big [\big (R_N^L(u)\big )^2\Big ]\\&= C_k \lim _{N \rightarrow \infty } \iint _{{\mathbb {T}}^2_L \times {\mathbb {T}}^2_L} {\mathbb {E}}_{\mu _L}\Big [ H_{k+1}({\textbf{P}}_N u(x); \sigma _{N, L}) H_{k+1}({\textbf{P}}_N u(y); \sigma _{N, L}) \Big ]dx dy \\&= C_k \lim _{N \rightarrow \infty } \iint _{{\mathbb {T}}^2_L \times {\mathbb {T}}^2_L} \Big ({\mathbb {E}}_{\mu _L}\big [ {\textbf{P}}_N u(x){\textbf{P}}_N u(y) \big ]\Big )^{k+1}dx dy \\&= C_k \lim _{N \rightarrow \infty } \iint _{{\mathbb {T}}^2_L \times {\mathbb {T}}^2_L} \bigg (\sum _{\begin{array}{c} \lambda \in {\mathbb {Z}}^2_L\\ |\lambda | \le N \end{array}} \frac{e_\lambda ^L(x-y)}{\langle \lambda \rangle ^2}\frac{1}{L} \bigg )^{k+1} dx dy \\&= C_k L^2 \lim _{N \rightarrow \infty }\sum _{\begin{array}{c} n_1, \dots , n_{k+1} \in {\mathbb {Z}}^2\\ n_1 + \cdots +n_{k+1} = 0\\ |n_j| \le LN \end{array}} \bigg (\prod _{j = 1}^{k+1}\frac{1}{\langle \frac{n_j}{L} \rangle ^2}\bigg ) \frac{1}{L^{2k}} \\&\sim L^2, \end{aligned}$$

where the last step follows from a Riemann sum approximation. See also Remark 1.4 (i). This non-uniformity causes difficulty in studying the large torus limit $L \rightarrow \infty$. In the one-dimensional case, there is no need for a renormalization in constructing a Gibbs measure, and Bourgain [14] used the Brascamp-Lieb concentration inequality [15, Theorem 5.1] to reduce the relevant analysis to that for the Gaussian case, uniformly in the period $L\gg 1$. In the current two-dimensional case, due to the use of the renormalization, the log-concavity needed for the Brascamp-Lieb concentration inequality is not available, and thus we need an alternative approach.

In this work, the main statistical control comes from the study on the so-called *enhanced Gibbs measures*, namely the distributions of the enhanced data sets. This idea played a crucial role in a recent work [70] on the hyperbolic $\Phi ^3_3$-model on the three-dimensional torus ${\mathbb {T}}^3$ by the first two authors and Okamoto. Given $R>0$, our goal is to construct a solution to (1.34) on the cone ${\textbf{C}}_R$ in (1.33) as a limit of the solution $u_L$ to the *L*-periodic problem (1.32); see (4.2) below. When $L \gg R$, we see that the *L*-periodic spatial white noise $\zeta _L$ defined in (3.53) and the *L*-periodic space-time white noise $\xi _L$ defined in (4.4) agree on the cone ${\textbf{C}}_R$ with the spatial white noise $\zeta$ on ${\mathbb {R}}^2$ (see Definition 3.4) and the space-time white noise on ${\mathbb {R}}^2\times {\mathbb {R}}_+$, respectively; see (4.6) below. Therefore, by restricting our attention to the cone ${\textbf{C}}_R$, we conclude from the finite speed of propagation and the observation above that the difference of the *L*-periodic problem (4.2) for different values of $L\ge 1$ appears *only in the first component* $u_{0, L}$ of the initial data with ${{\,\textrm{Law}\,}}(u_{0, L}) = \rho _L$, where $\rho _L$ is the *L*-periodic $\Phi ^{k+1}_2$-measure in (1.21). This essentially reduces the convergence problem on the cone ${\textbf{C}}_R$ of the *L*-periodic problem (4.2) to studying convergence properties of the enhanced Gibbs measure

1.36 $$\begin{aligned} \nu _L = {{\,\textrm{Law}\,}}(\Xi _0(u_{0, L})), \end{aligned}$$

where $\Xi _0(u_{0, L})$ denotes the enhanced data set,^14^ emanating from the first component $u_L|_{t = 0} = u_{0, L}$ of the initial data with ${{\,\textrm{Law}\,}}(u_{0, L}) = \rho _L$; see (4.36) and (4.12). As mentioned above, by restricting our attention to the cone ${\textbf{C}}_R$, when $L \gg R$, the second enhanced data set $\Xi _1(u_1, \xi )$ in (4.13) (see also (4.10)), involving the second component $u_1$ of the initial data and the space-time white noise forcing $\xi$, does *not* depend on *L*, which simplifies some analysis. As for the second enhanced data set $\Xi _1(u_1, \xi )$, its *mapping properties* (viewing its elements as (random) multiplication operators) play an important role; see Definition 4.1 and Proposition 4.9.

Let us briefly describe four main steps of the proof of Theorem 1.2.

$\bullet$ **Step 1:** Coming down from infinity for the associated SNLH on ${\mathbb {R}}^2$.

In this first step, we establish coming down from infinity (namely, an estimate on a solution independent of initial data) for SNLH (3.5) in (i) weighted Lebesgue spaces $L^p_\mu ({\mathbb {R}}^2)$ (Proposition 3.1) and (ii) weighted Sobolev spaces $W^{s, p}_\mu ({\mathbb {R}}^2)$ of positive regularities (Proposition 3.3). The coming down from infinity in weighted Lebesgue spaces yields tightness of the *L*-periodic Gibbs measures $\vec {\rho }_L$, which then allows us to extract a sequence $\{\vec {\rho }_L\}_{L \in {\mathcal {A}}}$ converging weakly to a limiting Gibbs measure $\vec {\rho }_\infty$; see Sect. 3.3. On the other hand, the coming down from infinity in weighted Sobolev spaces of positive regularities plays a crucial role in Step 3.

In recent years, coming down from infinity has been studied in the context of (singular) SNLH; see, for example, [58, 59, 96]. See the introduction in [58] for a further discussion. In the case of weighted Lebesgue spaces (Proposition 3.1), our argument follows closely that in [96] and aims to establish a certain differential inequality (see Lemma 3.2). Due to the use of the weight, however, the argument is more complicated and requires a careful decomposition of the physical space into unit cubes, combined with the Littlewood-Paley decomposition; see (3.16)-(3.26). In the case of weighted Sobolev spaces of positive regularities (Proposition 3.3), our argument is based on a Gronwall-type argument with the coming down from infinity for weighted Lebesgue spaces. We present details in Sect. 3.

$\bullet$ **Step 2:** Local well-posedness and stability of SdNLW (1.34) on the cone ${\textbf{C}}_R$.

This second step is entirely deterministic, by viewing initial data $(u_0, u_1)$ and a forcing $\xi$ as given deterministic spatial / space-time distributions, and follows from a slight modification of the local well-posedness argument in [45]. Here, stability refers to that with respect to the enhanced data set $\Xi _0(u_0)$ in (4.12), emanating from the first component $u_0$ of the initial data. See Sect. 4.1 for details.

$\bullet$ **Step 3:** Convergence of the enhanced Gibbs measures.

In this step and Step 4, we restrict our attention to $L\in {\mathcal {A}}\subset {\mathbb {N}}$ constructed in Step 1. This is due to the non-uniqueness of the limiting Gibbs measure on ${\mathbb {R}}^2$; see Remark 1.4 (ii).

Our main goal in this step is to prove convergence of $\{\nu _L\}_{L \in {\mathcal {A}}}$ to a natural limit

1.37 $$\begin{aligned} \nu _\infty = {{\,\textrm{Law}\,}}(\Xi _0(u_{0})) \end{aligned}$$

with ${{\,\textrm{Law}\,}}(u_0) = \rho _\infty$ constructed in Step 1. Here, the mode of convergence is weak convergence as well as convergence in the Wasserstein-1 metric. The proof is broken into two parts, where we first establish tightness of $\{\nu _L\}_{L \in {\mathcal {A}}}$ (Proposition 4.4) and then show that the limit is indeed unique, given by $\nu _\infty$ in (1.37) (Proposition 4.6).

The (first) enhanced data set $\Xi _0(u_{0, L})$ consists of the Wick powers $: \!({\mathcal {S}}(t) u_{0, L})^\ell \!:$ , $\ell = 1, \dots , k$, of the linear solution ${\mathcal {S}}(t) u_{0, L} = (\partial _t{\mathcal {D}}(t) + {\mathcal {D}}(t))u_{0, L}$ with ${{\,\textrm{Law}\,}}(u_{0, L}) = \rho _L$, where $\rho _L$ is the *L*-periodic $\Phi ^{k+1}$-measure in (1.21). Hence, in view of the invariance of $\rho _L$ under the parabolic $\Phi ^{k+1}_2$-model, instead of studying $\Xi _0(u_{0, L})$, it suffices to study $\Xi _0(X_L(1))$, where $X_L$ is the solution to the *L*-periodic SNLH (3.42) (namely, the parabolic $\Phi ^{k+1}_2$-model) with the Gibbsian initial data ${{\,\textrm{Law}\,}}(X_{0, L}) = \rho _L$. As a result, the argument involves an intricate combination of wave and heat analysis (see, in particular, the proof of Proposition 4.6) as well as the coming down from infinity in weighted Sobolev spaces of positive regularities. See Sect. 4.2 for details.

$\bullet$ **Step 4:** Global well-posedness and invariance of the limiting Gibbs measure.

We first prove well-posedness of the hyperbolic $\Phi ^{k+1}_2$-model on the cone ${\textbf{C}}_R$ for each $R > 0$. In view of the global well-posedness of the *L*-periodic hyperbolic $\Phi ^{k+1}_2$-model (4.2), the local well-posedness and stability results established in Step 2 allow us to reduce the problem to estimating the size of the first enhanced data set $\Xi _0(u_{0, L})$, studying the mapping properties of the second enhanced data set $\Xi _1(u_2, \xi )$, and convergence in the Wasserstein-1 metric of the enhanced Gibbs measure $\nu _L$ to $\nu _\infty$ established in Step 3. Invariance of the limiting Gibbs measure $\vec {\rho }_\infty$ then follows from the weak convergence of $\{\vec {\rho }_L\}_{L\in {\mathcal {A}}}$ to $\vec {\rho }_\infty$ (Theorem 1.2 (i)), the convergence in law, as ${\mathcal {D}}'({\mathbb {R}}_2\times {\mathbb {R}}_+)$-valued random variables, of the solution $u_L$, $L \in {\mathcal {A}}$, to the *L*-periodic hyperbolic $\Phi ^{k+1}_2$-model (4.2) to the solution *u* to the hyperbolic $\Phi ^{k+1}_2$-model (4.1) on ${\mathbb {R}}^2$, which follows as a corollary of the global well-posedness (Remark 4.12).

In view of the finite speed of propagation, when we work on the cone ${\textbf{C}}_R$, the idea of breaking the enhanced data set into two groups $\Xi _0(u_{0, L})$ in (4.12) and $\Xi _1(u_{1}, \xi )$ in (4.13), where the latter is independent of $L \gg R$, seems new but is natural. Let us make a brief comparison to the work [70] on the hyperbolic $\Phi ^3_3$-model by the first two authors and Okamoto. What is common is the use of the enhanced Gibbs measures (= the laws of the enhanced data sets of the *L*-periodic (or frequency-truncated) Gibbs measures), while, in the current paper, we only consider the enhanced Gibbs measure emanating from the first component of the initial data for the reason explained above. In this work, we reduce various problems to those for the associated stochastic heat equation, which leads to an interesting mixture of wave and heat analysis. Lastly, we remark that, in a recent remarkable preprint [18] resolving a challenging open problem on well-posedness of the hyperbolic $\Phi ^4_3$-model, a mixture of wave and heat analysis also played an important role (but in a different context from our analysis).

#### Remark 1.3

(i) A slight modification of the proof of Theorem 1.2 applies to the deterministic NLW (1.4) on ${\mathbb {R}}^2$ with the Gibbsian initial data, thus yielding global well-posedness of (the renormalized version of) (1.4) with ${{\,\textrm{Law}\,}}(u(0), \partial _tu(0)) = \vec {\rho }_\infty$ and invariance of $\vec {\rho }_\infty$ under the resulting dynamics.^15^ See [56] for the one-dimensional case. We also mention [14, 17, 20–22, 27, 51, 73, 98] for works on the construction of invariant Gibbs dynamics for Hamiltonian PDEs on unbounded domains, (including those with confining potentials).

(ii) In this paper, we only consider the defocusing case $k \in 2{\mathbb {N}}+1$. If (a) $k \in 2{\mathbb {N}}+2$ or (b) $k \in 2{\mathbb {N}}+ 1$ but with the − sign on the potential energy $\frac{1}{k+1}\int u^{k+1} dx$ in (1.2), namely, the focusing case, then it is known that the associated (renormalized) Gibbs measure on the two-dimensional torus ${\mathbb {T}}^2$ is not normalizable even with a taming by a power of the Wick-ordered $L^2$-norm; see [78]. See also [19]. We refer interested readers to [53, 69, 70, 79] on the (non-)construction of focusing Gibbs measures on the general *d*-dimensional torus.

When $k = 2$, it is possible to construct the Gibbs measure on ${\mathbb {T}}^2$ with a taming by a power of the Wick-ordered $L^2$-norm (see [13, 78]) and the associated invariant Gibbs dynamics for the hyperbolic $\Phi ^3_2$-model on ${\mathbb {T}}^2$; see Remark 1.8 in [81]. See also [70] for the three-dimensional case. Due to the non-defocusing nature of the problem, however, we expect a certain triviality phenomenon to take place in taking a large torus limit of the *L*-periodic $\Phi ^3_2$-measure, just as in the one-dimensional focusing case [85, 95].^16^

(iii) In [92, 94], the second author proved ergodicity of the Gibbs measure for the hyperbolic $\Phi ^{k+1}_d$-model posed on ${\mathbb {T}}^d$ when $d = 1, 2$. See also [33]. It is of interest to study the corresponding problem on ${\mathbb {R}}^d$.

#### Remark 1.4

(i) As mentioned above, the equivalence of the *L*-periodic $\Phi ^{k+1}_2$-measure $\rho _L$ and the base Gaussian free field $\mu _L$ on ${\mathbb {T}}^2_L$ is not uniform. In fact, it is expected that the limiting $\Phi ^{k+1}_2$-measure $\rho _\infty$ on ${\mathbb {R}}^2$ constructed in Theorem 1.2 (i) and the base Gaussian measure (= the large torus limit of $\mu _L$) are mutually singular. We will address this issue in a forthcoming work.

(ii) The limiting Gibbs measure $\rho _\infty$ constructed in Theorem 1.2 (i) in principle depends on the sequence $L \in {\mathcal {A}}$ and, in general, there is no uniqueness statement for the limiting Gibbs measure. See [34–36]. See also the discussion at the end of Sect. 2 in [3].

In a recent preprint [6] that appeared after the first version of the current paper, Bauerschmidt, Dagallier, and Weber proved uniqueness of the $\Phi ^4_2$-measure on the plane in the high temperature regime. Namely, by replacing $\frac{1}{k+1}$ in (1.21) by $\frac{\beta }{k+1}$ with $k = 3$, they proved that there exists $\beta _* > 0$ such that the $\Phi ^4_2$-measure on the plane with the inverse temperature $\beta$ is unique for $0< \beta < \beta _*$. As a consequence, when $k = 3$, by replacing $\frac{1}{k+1}$ in (1.20) by $\frac{\beta }{k+1}$ and $:\!u^k\!:$ by $\beta :\!u^k\!:$ in (1.34), the convergence claims in Theorem 1.2 hold for the entire families $\{\vec {\rho }_L\}_{L \ge 1}$ and $\{(u_{L}, \partial _tu_{L})\}_{L \ge 1}$, thus giving uniqueness of the invariant hyperbolic $\Phi ^4_2$-dynamics on the plane. While we expect that the argument in [6] to hold also for a higher power (which then would imply uniqueness of the invariant hyperbolic $\Phi ^{k+1}_2$-dynamics on the plane), we do not pursue this issue here. See also a very recent preprint [31] for uniqueness of the $\Phi ^4_3$-measure on ${\mathbb {R}}^3$ in the high temperature regime.

#### Remark 1.5

Let $\rho _\infty$ be the limiting $\Phi ^{k+1}$-measure in Theorem 1.2 (i) constructed as a limit of $\{\rho _L\}_{L \in {\mathcal {A}}}$. Consider the parabolic $\Phi ^{k+1}_2$-model (3.1) on ${\mathbb {R}}^2$ with the Gibbsian initial data ${{\,\textrm{Law}\,}}(X_0) = \rho _\infty$. Then, it follows from the proof of Proposition 4.6 (see Remark 4.7) that $\rho _\infty$ is invariant under the parabolic $\Phi ^{k+1}_2$-dynamics on ${\mathbb {R}}^2$. While this fact is not difficult to prove, to the authors’ knowledge, it is not explicitly written and thus we decided to mention it here.

## Preliminary

### Notations

By $A\lesssim B$, we mean $A\le CB$ for some constant $C> 0$. We use $A \sim B$ to mean $A\lesssim B$ and $B\lesssim A$. We write $A\ll B$, if there is some small $c>0$ such that $A\le cB$. We may use subscripts to denote dependence on external parameters; for example, $A\lesssim _{\delta } B$ means $A\le C(\delta ) B$. We use $a-$ (and $a+$) to denote $a- \varepsilon$ (and $a+ \varepsilon$, respectively) for arbitrarily small $\varepsilon > 0$. If this notation appears in an estimate, then an implicit constant is allowed to depend on $\varepsilon > 0$ (and it usually diverges as $\varepsilon \rightarrow 0$). We also use the notation $p = \infty -$ to denote a sufficiently large number $p \gg 1$, depending on the context. For conciseness of notation, we set $a \vee b = \max (a, b)$.

Throughout this paper, we fix a rich enough probability space $(\Omega , {\mathcal {F}}, {\mathbb {P}})$, on which all the random objects are defined. The realization $\omega \in \Omega$ is often omitted in the writing. Given a random variable *X*, we denote by ${{\,\textrm{Law}\,}}(X)$ the law of *X*.

In the remaining part of the paper, we only work with real-valued functions / distributions. Given a time-differentiable function, we set $\vec {u} = (u, \partial _tu)$.

We define the Fourier transform of a function *f* on ${\mathbb {R}}^d$ by setting

$$\begin{aligned} \widehat{f}(\eta ) = {\mathcal {F}}(f) (\eta ) = \int _{{\mathbb {R}}^d} f(x) e^{- 2\pi i \eta \cdot x} dx\end{aligned}$$

with the inverse Fourier transform given by ${\mathcal {F}}^{-1}(f) (\eta ) = \widehat{f}(-\eta )$.

Let $s \in {\mathbb {R}}$ and $1 \le p \le \infty$. We define the $L^2$-based Sobolev space $H^s({\mathbb {R}}^d)$ by the norm:

$$\begin{aligned} \Vert f \Vert _{H^s} = \Vert \langle \nabla \rangle ^s f\Vert _{L^2} = \Vert \langle \eta \rangle ^s \widehat{f} (\eta ) \Vert _{L^2}, \end{aligned}$$

where $\langle \,\cdot \, \rangle = (1 + |\cdot |^2)^\frac{1}{2}$. We also define the $L^p$-based Sobolev space $W^{s, p}({\mathbb {R}}^d)$ by the norm:

2.1 $$\begin{aligned} \Vert f \Vert _{W^{s, p}} = \Vert \langle \nabla \rangle ^s f\Vert _{L^p} = \big \Vert {\mathcal {F}}^{-1} [\langle \eta \rangle ^s \widehat{f}(\eta )] \big \Vert _{L^p}. \end{aligned}$$

When $p = 2$, we have $H^s({\mathbb {R}}^d) = W^{s, 2}({\mathbb {R}}^d)$. We recall the following interpolation result which follows from the Littlewood-Paley characterization of Sobolev norms via the square function and Hölder’s inequality; let $s, s_1, s_2 \in {\mathbb {R}}$ and $p, p_1, p_2 \in (1,\infty )$ such that $s = \theta s_1 + (1-\theta ) s_2$ and $\frac{1}{p} = \frac{\theta }{p_1} + \frac{1-\theta }{p_2}$ for some $0< \theta < 1$. Then, we have

2.2 $$\begin{aligned} \Vert u \Vert _{W^{s, p}} \lesssim \Vert u \Vert _{W^{s_1, p_1}}^\theta \Vert u \Vert _{W^{s_2, p_2}}^{1-\theta }. \end{aligned}$$

Let $X({\mathbb {R}}^d)$ be a function space on ${\mathbb {R}}^d$ such as the Sobolev space $W^{s, p}({\mathbb {R}}^d)$ defined above or the weighted Sobolev / Besov spaces defined below. Given a (nice) subset $K \subset {\mathbb {R}}^d$, we define the localized version of a function space $X({\mathbb {R}}^d)$ by the restriction norm:

2.3 $$\begin{aligned} \Vert f\Vert _{X(K)} = \inf \big \{ \Vert g \Vert _{X({\mathbb {R}}^d)}: \, f \equiv g \text { on } K\big \}. \end{aligned}$$

In dealing with a space of space-time functions, we often use short-hand notations such as $L^q_T H^s_x$ for $L^q([0, T]; H^s({\mathbb {R}}^d))$. Given a space $X({\mathbb {R}}^d)$ of functions on ${\mathbb {R}}^d$, we set $L^\infty ([0, R]; X(B_{R-t}))$ by

2.4 $$\begin{aligned}&L^\infty ([0, R]; X(B_{R-t})) =\nonumber \\&\quad \big \{ u \in {\mathcal {D}}'(\mathring{\textbf{C}}_R): t \in [0, R] \mapsto \Vert u(t)\Vert _{X(B_{R-t})}\text { is in }L^\infty \big \}, \end{aligned}$$

where $\mathring{\textbf{C}}_R$ denotes the interior of the cone ${\textbf{C}}_R$ and $X(B_{R-t})$ is defined by the restriction norm as in (2.3). Here, $B_R \subset {\mathbb {R}}^d$ denotes the (closed) ball of radius *R* centered at the origin and ${\textbf{C}}_R$ denotes the cone as in (1.33) (but in ${\mathbb {R}}^d \times {\mathbb {R}}_+$).

Let ${\mathcal {D}}(t)$ denote the linear damped wave propagator defined in (1.23). We then set

2.5 $$\begin{aligned} {\mathcal {S}}(t) = \partial _t{\mathcal {D}}(t) + {\mathcal {D}}(t). \end{aligned}$$

Namely, $$u = {\mathcal {S}}(t) u_0$$ satisfies

$$\begin{aligned} {\left\{ \begin{array}{ll} \partial _t^2 u + \partial _tu + (1-\Delta ) = 0\\ (u, \partial _tu)|_{t= 0} = (u_0, 0). \end{array}\right. } \end{aligned}$$

Let $p_t$ denote the standard heat kernel on ${\mathbb {R}}^2$ given by $p_t(x) = \frac{1}{4\pi t} e^{- \frac{|x|^2}{4t} }$. Then, let *P*(*t*) denote the linear heat propagator given by

2.6 $$\begin{aligned} P(t)f = e^{t(\Delta - 1)} f = e^{-t} (p_t*f). \end{aligned}$$

We now introduce the Littlewood-Paley projector, which is adapted to weighted Sobolev and Besov spaces defined in the next subsection. Recall the following definition [60, Definition 1].^17^ Given $\theta _0 \ge 1$, the Gevrey class ${\mathcal {G}}^{\theta _0} \subset C^\infty ({\mathbb {R}}^d; {\mathbb {R}})$ of order $\theta _0$ consists of functions *f* satisfying the following; given any compact set $K \subset {\mathbb {R}}^d$, there exists $C_K > 0$ such that $\sup _{x \in K}|\partial ^\alpha f(x)| \le (C_K)^{|\alpha |+1} (\alpha !)^{\theta _0}$ for any multi-index $\alpha$. We use ${\mathcal {G}}^{\theta _0}_c$ to denote the set of compactly supported functions in ${\mathcal {G}}^{\theta _0}$.

Let ${\mathbb {Z}}_{\ge 0}:= {\mathbb {N}}\cup \{0\}$. Let us now introduce the Littlewood-Paley projector ${\textbf{Q}}_k$, $k \in {\mathbb {Z}}_{\ge 0}$, defined by Gevrey class multipliers. As seen in [60], such a choice is suitable for weighted spaces with a stretched exponential weight; see (2.10). As in Sect. 3 of [60], let

2.7 $$\begin{aligned} \chi _0, \chi _1 \in {\mathcal {G}}^{\theta _0}_c({\mathbb {R}}^d; [0, 1]) \end{aligned}$$

with

$$\begin{aligned} \mathop {\textrm{supp}}\limits \chi _0 \subset \big \{|\xi |\le \tfrac{4}{3}\big \} \qquad \text {and}\qquad \mathop {\textrm{supp}}\limits \chi _1 \subset \big \{\tfrac{3}{4} \le |\xi |\le \tfrac{8}{3}\big \} \end{aligned}$$

such that

$$\begin{aligned} \sum _{k = 0}^\infty \chi _k(\xi ) \equiv 1 \end{aligned}$$

on ${\mathbb {R}}^d$, where $\chi _k(\xi ) = \chi _1(2^{1-k} \xi )$ for $k \ge 2$. We then define the Littlewood-Paley projector ${\textbf{Q}}_k$ by

2.8 $$\begin{aligned} {\textbf{Q}}_k(f) = {\mathcal {F}}^{-1} (\chi _k \widehat{f}) = \eta _k * f, \end{aligned}$$

where $\eta _k$ is defined by

2.9 $$\begin{aligned} \eta _k = {\mathcal {F}}^{-1}(\chi _k) \end{aligned}$$

for $k \in {\mathbb {Z}}_{\ge 0}$. Namely, we have $\eta _k(x) = 2^{dk} \eta (2^kx)$ for $k \in {\mathbb {N}}$, where $\eta (x) = 2^{-d}\eta _1(2^{-1} x)$. We also recall Proposition 1 in [60]^18^ that

2.10 $$\begin{aligned} |\eta (x)| \lesssim e^{-c|x|^\frac{1}{\theta _0}}. \end{aligned}$$

### Weighted Sobolev and Besov spaces

In this subsection, we introduce weighted Sobolev and Besov spaces and discuss their basic properties. We first recall the definition of the stretched exponential weight introduced in [60]. Let $\theta _0$ be as in (2.7), appearing in the definition of the Littlewood-Paley projector ${\textbf{Q}}_k$ in (2.8). Given $0< \delta < \frac{1}{\theta _0}$ ($\le 1$) and $\mu > 0$, we define the weight $w_\mu (x)$ by setting

2.11 $$\begin{aligned} w_\mu (x) = e^{-\mu \langle x \rangle ^\delta }. \end{aligned}$$

It is easy to check that the weight $w_\mu$ is $w_{-\mu }$-moderate in the sense:

2.12 $$\begin{aligned} w_\mu (x+y) \le w_{-\mu }(x) w_\mu (y) \end{aligned}$$

for any $x, y \in {\mathbb {R}}^2$; see [60, (2.6)]. In particular, it follows from (2.12) that

2.13 $$\begin{aligned} w_\mu (x) \lesssim w_\mu (n) \end{aligned}$$

for any $x \in Q_n:= n + \big [-\frac{1}{2}, \frac{1}{2}\big )^2$. Let $0< \alpha < 1$. Then, recall from [60, (3.25)]^19^ that we have

2.14 $$\begin{aligned} |{\textbf{Q}}_k w_\mu (x)| \lesssim 2^{-\alpha k}w_\mu (x) \end{aligned}$$

for any $x \in {\mathbb {R}}^2$ and $k \in {\mathbb {Z}}_{\ge 0}$. In the remaining part of the paper, we fix $0< \delta < \frac{1}{\theta _0}$ in (2.11) and we often drop the dependence on $\delta$ in various estimates.

Let $1 \le p < \infty$ and $\mu > 0$. Then, the weighted Lebesgue space $L^p_\mu ({\mathbb {R}}^d)$ is defined by the norm:

2.15 $$\begin{aligned} \Vert f \Vert _{L^p_\mu } =\bigg (\int _{{\mathbb {R}}^d} |f(x)|^p w_\mu (x) dx\bigg )^\frac{1}{p}. \end{aligned}$$

While it is possible to introduce a weighted Sobolev space by the $L^p_\mu$-norm of $\langle \nabla \rangle ^s f$ (see (2.31) below), we use a slightly different definition by first introducing spatial localization.

Let $\phi :{\mathbb {R}}\rightarrow [0,1]$ be a smooth even function such that $\phi$ is non-increasing on ${\mathbb {R}}_+$, $\phi =1$ on $\big [-\frac{5}{4}, \frac{5}{4}\big ]$, and $\phi =0$ on $(-\infty , -\frac{8}{5}\big ]\cup \big [\frac{8}{5}, \infty )$. Given $j \in {\mathbb {Z}}_{\ge 0}$, we define $\phi _j$ by

2.16 $$\begin{aligned} \phi _0(x) = \phi (|x|) \qquad \textrm{and} \qquad \phi _j(x) = \phi \big (\tfrac{|x|}{2^{j}}\big ) - \phi \big (\tfrac{|x| }{2^{j-1}}\big ), \ \ j \in {\mathbb {N}}. \end{aligned}$$

Then, it is easy to check that

2.17 $$\begin{aligned} \sum _{j=0}^\infty \phi _j(x) =1 \,\,\,\, \text {for any }x\in {\mathbb {R}}^d, \end{aligned}$$

and

2.18 $$\begin{aligned} \mathop {\textrm{supp}}\limits \phi _0 \subset \big \{ |x| \le \tfrac{8}{5}\} \quad \text {and}\quad \mathop {\textrm{supp}}\limits \phi _j \subset \big \{ \tfrac{5}{8} \cdot 2^j \le |x| \le \tfrac{8}{5} \cdot 2^j \}, \ \ j \in {\mathbb {N}}. \end{aligned}$$

By convention, we set $\phi _{-1} = 0$. Given $s \ge 0$ and $1 \le p \le \infty$, it follows from (2.16) that

2.19 $$\begin{aligned} \Vert \phi _j\Vert _{W^{s, p}({\mathbb {R}}^d)} \sim 2^{\frac{d}{p} j}. \end{aligned}$$

We also define slightly fattened cutoff functions $\widetilde{\phi }_j$, $j \in {\mathbb {Z}}_{\ge 0}$, by setting

2.20 $$\begin{aligned} \widetilde{\phi }_j = \phi _{j-1} + \phi _j + \phi _{j+1}, \ \ j \in {\mathbb {N}}. \end{aligned}$$

Then, we have

2.21 $$\begin{aligned} \widetilde{\phi }_j \phi _j = \phi _j. \end{aligned}$$

for any $$j\in {\mathbb {Z}}_{\ge 0}$$.

Given $1 \le p < \infty$ and $\mu > 0$, we define the weighted Sobolev space $W^{s,p}_\mu ({\mathbb {R}}^d)$ by the norm:

2.22 $$\begin{aligned} \Vert f \Vert _{W^{s,p}_\mu } = \bigg (\sum _{j=0 }^\infty w_\mu (2^{j})\Vert \phi _j f\Vert _{W^{s,p}}^p\bigg )^\frac{1}{p} , \end{aligned}$$

where $W^{s,p} = W^{s,p}({\mathbb {R}}^d)$ denotes the usual $L^p$-based Sobolev space on ${\mathbb {R}}^d$ defined in (2.1). When $p = 2$, we set

$$\begin{aligned}H^s_\mu ({\mathbb {R}}^d) = W^{s, 2}_\mu ({\mathbb {R}}^d).\end{aligned}$$

The following embedding follows from the definition (2.22) with Lemma 2.8 and (2.19):

$$\begin{aligned}W^{s, p_1}_{\mu _1} ({\mathbb {R}}^d) \subset W^{s, p_2}_{\mu _2}({\mathbb {R}}^d) \quad \text { for }1 \le p_2 \le p_1 < \infty \text { and }\mu _2 \ge \mu _1 > 0. \end{aligned}$$

From (2.22) and (2.15) with (2.11) and (2.18), there exists $a_1 > 0$ such that

2.23 $$\begin{aligned} \begin{aligned} \Vert f \Vert _{W^{s,p}_\mu }&\le \sum _{j= 0}^\infty w_{\frac{1}{p} \mu }(2^j) \Vert \phi _j f \Vert _{W^{s,p} } \le \sum _{j= 0}^\infty e^{- \frac{\mu }{p} 2^{j\delta } } \Vert \phi _j f \Vert _{W^{s,p} }, \\ \Vert \phi _j f \Vert _{W^{s,p}}&\le e^\frac{\mu }{p} e^{ \frac{\mu }{p} 2^{j\delta } } \Vert f \Vert _{W^{s,p}_\mu } ,\\ \Vert \phi _j f \Vert _{L^p}&\le e^{ \frac{\mu }{p} a_1 2^{j\delta } } \Vert f \Vert _{L^p_\mu } \end{aligned} \end{aligned}$$

for any $j \in {\mathbb {Z}}_{\ge 0}$. Similar bounds hold when we replace $\phi _j$ by $\widetilde{\phi }_j$.

Let $1 \le q \le p < \infty$. By applying Sobolev’s inequality (with $\frac{s}{d} \ge \frac{1}{q} - \frac{1}{p}$) and the embedding $\ell ^q({\mathbb {Z}}_{\ge 0}) \subset \ell ^p({\mathbb {Z}}_{\ge 0})$, we have

2.24 $$\begin{aligned} \begin{aligned} \Vert f \Vert _{W^{0,p}_\mu }&= \big \Vert w_{\frac{\mu }{p} } (2^{j})\Vert \phi _j f\Vert _{L^{p}}\big \Vert _{\ell ^p_j({\mathbb {Z}}_{\ge 0})}\\&\lesssim \big \Vert w_{\frac{\mu }{p} } (2^{j})\Vert \phi _j f\Vert _{W^{s, q}}\big \Vert _{\ell ^q_j({\mathbb {Z}}_{\ge 0})}\\&= \Vert f \Vert _{W^{s,q}_{\frac{q}{p}\mu }} . \end{aligned} \end{aligned}$$

#### Remark 2.1

We point out that when $s = 0$, the $W^{0, p}_\mu$-norm defined in (2.22) is not equivalent to the $L^p_\mu$-norm defined in (2.15). See also Remark 2.4 below.

We also recall the definition of the weighted Besov space $B^{s, \mu }_{p, q}({\mathbb {R}}^d)$ from [60, Section 3]. Given $s \in {\mathbb {R}}$, $1 \le p, q \le \infty$, and $\mu \ge 0$, we define the weighted Besov space $B^{s, \mu }_{p, q}({\mathbb {R}}^d)$ as the completion of $C^\infty _c({\mathbb {R}}^d)$ under the norm:

2.25 $$\begin{aligned} \Vert f\Vert _{B^{s, \mu }_{p, q}} = \Big \Vert 2^{sk} \Vert {\textbf{Q}}_k f\Vert _{L^p_\mu ({\mathbb {R}}^2)}\Big \Vert _{\ell ^q_k({\mathbb {Z}}_{\ge 0})}. \end{aligned}$$

See also [41]. When $\mu = 0$, the weighted Besov space $B^{s, \mu }_{p, q}({\mathbb {R}}^d)$ reduces to the usual Besov space $B^{s}_{p, q}({\mathbb {R}}^d)$. We recall the following embeddings for (unweighted) Sobolev and Besov spaces:

2.26 $$\begin{aligned} \Vert f \Vert _{B^s_{p, \infty }} \lesssim \Vert f\Vert _{W^{s, p}} \lesssim \Vert f \Vert _{B^s_{p, 1}} \lesssim \Vert f \Vert _{B^{s+\varepsilon }_{p, \infty }} \end{aligned}$$

for any $$\varepsilon > 0$$.

We first establish the following compact embedding for weighted Besov spaces, which plays a crucial role in proving Theorem 1.2 (i).

#### Lemma 2.2

Let $1 \le p < \infty$. Then, given any $s > s'$ and $\mu < \mu '$, the embedding

$$\begin{aligned} W^{s, p}_{\mu } ({\mathbb {R}}^d) \hookrightarrow W^{s', p}_{\mu '}({\mathbb {R}}^d) \end{aligned}$$

is compact.

#### Proof

Let $\{ f_n\}_{n\in {\mathbb {N}}}$ be a bounded sequence in $W^{s,p}_\mu ({\mathbb {R}}^d)$. Then it follows from (2.22) and (2.18) that for any $j\in {\mathbb {N}}$, the sequence $\{ \phi _j f_n\}_{ n\in {\mathbb {N}}}$ is a bounded sequence in $W^{s,p}({\mathbb {R}}^2)$ with a bounded support: $\big \{ |x| \le \tfrac{8}{5} \cdot 2^j \}$, $j \in {\mathbb {Z}}_{\ge 0}$.

In the following, we implement a diagonal argument. Let $$j = 0$$. Then, by Rellich’s lemma (with $$s' < s$$), there exists a subsequence $$\{ \phi _0 f_{n_k^{(0)}}\}_{ k\in {\mathbb {N}}}$$ which is convergent in $$W^{s',p}({\mathbb {R}}^d)$$. Then, for $$j \in {\mathbb {N}}$$, by Rellich’s lemma, we can choose a subsequence $$\{n_k^{(j)}\}_{k \in {\mathbb {N}}}$$ of $$\{n_k^{(j-1)}\}_{k \in {\mathbb {N}}}$$ such that $$\{ \phi _j f_{n_k^{(j)}}\}_{ k\in {\mathbb {N}}}$$ is convergent in $$W^{s',p}({\mathbb {R}}^d)$$. Now, consider the sequence $$\{ \phi _j f_{n_k^{(k)}}\}_{ k\in {\mathbb {N}}}$$. Then, it is clear from the construction that, for each $$j \in {\mathbb {Z}}_{\ge 0}$$, $$ \phi _j f_{n_k^{(k)}}$$ converges to some $$F_j $$ in $$W^{s',p}({\mathbb {R}}^d)$$ as $$k \rightarrow \infty $$.

By setting

$$\begin{aligned} F = \sum _{j = 0}^\infty F_j, \end{aligned}$$

we claim that $F\in W^{s', p}_{\mu '}({\mathbb {R}}^d)$ for any $\mu ' > \mu$ and that $f_{n_k^{(k)}}$ converges to *F* in $W_{\mu '}^{s', p}({\mathbb {R}}^d)$ for $\mu '> 2^\delta \mu$.

First, note that we have

2.27 $$\begin{aligned} \Vert \phi _j f \Vert _{W^{s', p}} \lesssim \Vert f \Vert _{W^{s', p}}, \end{aligned}$$

uniformly in $j \in {\mathbb {Z}}_{\ge 0}$. When $s' = 0$, (2.27) follows from Hölder’s inequality. When $s' < 0$, it follows from Lemma 2.8 (ii) and (2.19) that

$$\begin{aligned} \Vert \phi _j f \Vert _{W^{s', p}} \lesssim \Vert \phi _j \Vert _{W^{- s', \infty }} \Vert f \Vert _{W^{s', p}} \lesssim \Vert f \Vert _{W^{s', p}}. \end{aligned}$$

When $s' > 0$, the fractional Leibniz rule (see Lemma 2.8 (i) below), (2.19), and Sobolev’s inequality yield

$$\begin{aligned} \Vert \phi _j f \Vert _{W^{s', p}} \lesssim \Vert \phi _j \Vert _{W^{s', \infty }} \Vert f \Vert _{W^{s', p}} \lesssim \Vert f \Vert _{W^{s', p}}. \end{aligned}$$

This proves the bound (2.27). Then, from (2.23), (2.18), (2.27), $w_{\mu '}(2^j) = w_{\mu }(2^j) w_{\mu '-\mu }(2^j)$, and the uniform bound on the $W^{s, p}_\mu$-norm of $f_{n_k^{(k)}}$, we have

$$\begin{aligned} \Vert F\Vert _{W_{\mu '}^{s', p}}&\le \sum _{j = 0}^\infty \Vert F_j\Vert _{W_{\mu '}^{s', p}} \le \sum _{j = 0}^\infty \sum _{\ell = 0}^\infty w_{\frac{1}{p} \mu '}(2^\ell ) \Vert \phi _\ell F_j \Vert _{W^{s', p}}\\&\le \sum _{j = 0}^\infty \sum _{\ell = 0}^\infty w_{\frac{1}{p} \mu '}(2^\ell ) \lim _{k \rightarrow \infty } \Vert \phi _\ell \phi _j f_{n_k^{(k)}} \Vert _{W^{s', p}}\\&= \sum _{\ell = 0}^\infty \sum _{i = -1}^1 w_{\frac{1}{p} \mu '}(2^\ell ) \lim _{k \rightarrow \infty } \Vert \phi _\ell \phi _{\ell + i} f_{n_k^{(k)}} \Vert _{W^{s', p}}\\&\lesssim \sum _{\ell = 0}^\infty w_{\frac{1}{p} \mu '}(2^\ell ) \lim _{k \rightarrow \infty } \Vert \phi _\ell f_{n_k^{(k)}} \Vert _{W^{s, p}}\\&\le \sum _{\ell = 0}^\infty w_{\frac{1}{p} (\mu '-\mu )}(2^\ell ) \cdot \sup _{k \in {\mathbb {N}}} \Big ( w_{\frac{1}{p} \mu }(2^\ell ) \Vert \phi _\ell f_{n_k^{(k)}}\Vert _{W^{s, p}}\Big )\\&< \infty , \end{aligned}$$

provided that $s' < s$ and $\mu ' > \mu$.

Next, we show convergence. First, note that, as a limit of $\phi _j f_{n_k^{(k)}}$, we have $F_j(x) = 0$ whenever $\phi _j(x) = 0$. Thus, in view of (2.18), we can write $F_j = \phi _j G_j$ with a well-defined function $G_j = {\textbf{1}}_{\phi _j \ne 0 } \cdot F_j / \phi _j$ and thus, we have

2.28 $$\begin{aligned} f_{n_k^{(k)}} - F = \sum _{j = 0}^\infty \phi _j ( f_{n_k^{(k)}} - G_j) . \end{aligned}$$

We set $$F_{-1} = G_{-1} = 0$$.

From (2.22), we have

2.29 $$\begin{aligned} \begin{aligned} \Vert f_{n_k^{(k)}} - F \Vert _{W^{s',p}_{\mu '}}^p&= \sum _{j = 0}^\infty w_{\mu '}(2^j) \Vert \phi _j ( f_{n_k^{(k)}} -F) \Vert _{W^{s', p}}^p. \end{aligned} \end{aligned}$$

Then, from (2.28) with (2.18) followed by (2.27) we have

2.30 $$\begin{aligned} \begin{aligned} w_{\mu '}(2^j) \Vert \phi _j ( f_{n_k^{(k)}} -F) \Vert _{W^{s', p}}^p&= w_{\mu '}(2^j) \bigg \Vert \phi _j \sum _{i= - 1}^1 \phi _{j+i} (f_{n_k^{(k)}} - G_{j+i}) \bigg \Vert _{W^{s', p}}^p\\&\lesssim w_{\mu '}(2^j) \bigg \Vert \sum _{i= - 1}^1 \phi _{j+i} (f_{n_k^{(k)}} - G_{j+i}) \bigg \Vert _{W^{s', p}}^p\\&\lesssim \sum _{\ell = j- 1}^{j+1} w_{\mu '}(2^{\ell -1}) \Vert \phi _{\ell } (f_{n_k^{(k)}} - G_{\ell }) \Vert _{W^{s', p}}^p\\&= \sum _{\ell = j- 1}^{j+1} w_{\mu '}(2^{\ell -1}) \Vert \phi _{\ell } f_{n_k^{(k)}} - F_{\ell } \Vert _{W^{s', p}}^p. \end{aligned} \end{aligned}$$

For each $j \in {\mathbb {Z}}_{\ge 0}$, the right-hand side of (2.30) tends to 0 as $k \rightarrow \infty$. Namely, for each $j \in {\mathbb {Z}}_{\ge 0}$, the summand on the right-hand side of (2.29) tends to 0 as $k \rightarrow \infty$. On the other hand, in view of the uniform bound on the $W^{s, p}_\mu$-norm of $f_{n_k^{(k)}}$ and the fact that $F\in W^{s', p}_{\mu '}({\mathbb {R}}^d)$, we have

$$\begin{aligned} w_{\mu '}(2^j)&\Vert \phi _j ( f_{n_k^{(k)}} -F) \Vert _{W^{s', p}}^p\\&\lesssim w_{\mu '}(2^j) \Vert \phi _j f_{n_k^{(k)}} \Vert _{W^{s', p}}^p + w_{\mu '}(2^j) \Vert \phi _j F \Vert _{W^{s', p}}^p, \end{aligned}$$

which is summable in *j*. Therefore, by the dominated convergence theorem applied to (2.29), we conclude that $f_{n_k^{(k)}}$ converges to *F* in $W^{s',p}_{\mu '}({\mathbb {R}}^d)$ as $k \rightarrow \infty$. This concludes the proof of Lemma 2.2. $\square$

There seems to be no embedding relation analogous to (2.26) for weighted Sobolev spaces $W^{s, p}_\mu ({\mathbb {R}}^d)$ in (2.22) and weighted Besov spaces. With a slight loss in *s* and $\mu$, however, we have the following embeddings for weighted Sobolev spaces and weighted Besov spaces. We present the proof in Appendix A.

#### Lemma 2.3

Let $s \in {\mathbb {R}}$, $1 \le p < \infty$, and $\mu > 0$. Then, there exist $c_1, c_2 > 0$ such that

$$\begin{aligned} \Vert f \Vert _{W^{s, p}_\mu } \lesssim \Vert f\Vert _{B^{s', \mu '}_{p, 1}} \end{aligned}$$

for any $s' > s$ and $0< \mu ' < c_1 \mu$, and

$$\begin{aligned} \Vert f\Vert _{B^{s, \mu }_{p, \infty }} \lesssim \Vert f \Vert _{W^{s, p}_{\mu '}} \end{aligned}$$

for any $$0< \mu ' < c_2 \mu $$.

#### Remark 2.4

Another way to define a weighted Sobolev space would be to define a space ${\textbf{W}}^{s, p}_\mu ({\mathbb {R}}^2)$ via the norm:

2.31 $$\begin{aligned} \Vert f \Vert _{{\textbf{W}}^{s, p}_\mu } = \Vert \langle \nabla \rangle ^s f \Vert _{L^p_\mu }. \end{aligned}$$

When $s = 0$, we have ${\textbf{W}}^{0, p}_\mu ({\mathbb {R}}^2) = L^p_\mu ({\mathbb {R}}^2)$. Furthermore, this weighted Sobolev space ${\textbf{W}}^{s, p}_\mu ({\mathbb {R}}^2)$ is compatible with the weighted Besov space defined in (2.25) in the following sense:

2.32 $$\begin{aligned} \Vert f \Vert _{B^{s, \mu }_{p, \infty }} \lesssim \Vert f\Vert _{{\textbf{W}}^{s, p}_\mu } \lesssim \Vert f \Vert _{B^{s, \mu }_{p, 1}}, \end{aligned}$$

where the first inequality follows from Young’s inequality with [60, Lemma 1]^20^ while the second inequality follows from Minkowski’s inequality. It follows from Lemma 2.3 and (2.32) that

$$\begin{aligned} \Vert f\Vert _{{\textbf{W}}^{s - \varepsilon , p}_{\mu _1}} \lesssim \Vert f \Vert _{W^{s, p}_\mu } \lesssim \Vert f\Vert _{{\textbf{W}}^{s + \varepsilon , p}_{\mu _2}}. \end{aligned}$$

for any $\varepsilon > 0$ and $\mu _1 \gg \mu \gg \mu _2> 0$.

In this paper, we use the weighted Sobolev space $W^{s, p}_\mu ({\mathbb {R}}^2)$ defined in (2.22) since it is more convenient to work with $W^{s, p}_\mu ({\mathbb {R}}^2)$ in establishing coming down from infinity in a weighted Sobolev spaces of positive regularities.

See Remark 2.7 for a discussion on the bounded property of the linear damped wave propagator on ${\textbf{W}}^{s, 2}_\mu ({\mathbb {R}}^d)$.

### Linear estimates on weighted Sobolev spaces

In this subsection, we establish linear estimates for the linear heat propagator and the linear damped wave propagator on weighted Sobolev spaces.

#### Lemma 2.5

There exist small $C_0, c > 0$ such that for any $s \ge 0$, $1 \le p < \infty$, $\mu > 0$, and $0< \mu ' < C_0 \mu$, we have

2.33 $$\begin{aligned} \Vert P(t) f \Vert _{W^{s, p}_{\mu }({\mathbb {R}}^d)} \lesssim t^{-\frac{s}{2}} e^{-c t} \Vert f \Vert _{L^p_{\mu '}({\mathbb {R}}^d)} \end{aligned}$$

for any $t>0$, where $P(t) = e^{t (\Delta -1)}$ is as in (2.6).

#### Proof

We first introduce a cutoff function $\phi _m^t$ in terms of the self-similar variable $\frac{x}{t^\frac{1}{2}}$ for the (homogeneous) heat equation $\partial _tu - \Delta u = 0$ by setting

2.34 $$\begin{aligned} \phi _m^t(x) := \phi _m\bigg ( \frac{x}{ t^{\frac{1}{2}}} \bigg ) \end{aligned}$$

for $m\in {\mathbb {Z}}_{\ge 0}$. Then, given $j \in {\mathbb {Z}}_{\ge 0}$, it follows from (2.17), (2.18), and (2.34) that

2.35 $$\begin{aligned} \begin{aligned} \phi _j (P(t) f)&= e^{-t} \sum _{m, m' = 0}^\infty \phi _j \big [ (\phi _m^t p_t)*( \phi _{m'} f) \big ] \\&= e^{-t} \sum _{(m, m') \in \Lambda _{t, j}} \phi _j \big [ (\phi _m^t p_t)*( \phi _{m'} f) \big ], \end{aligned} \end{aligned}$$

where $\Lambda _{t, j} \subset ({\mathbb {Z}}_{\ge 0})^2$ is given by

2.36 $$\begin{aligned} \begin{aligned}&\Lambda _{t, j} = \Big \{(m, m') \in ({\mathbb {Z}}_{\ge 0})^2:\ \tfrac{5}{8}\cdot 2^{m'} \cdot {\textbf{1}}_{\{ m'\ge 1 \}} \le \tfrac{8}{5} \cdot 2^j + \tfrac{8}{5} \cdot 2^{m} t^{\frac{1}{2}} \\&\text {and }\, \tfrac{5}{8}\cdot 2^{m}t^\frac{1}{2} \cdot {\textbf{1}}_{\{ m\ge 1 \}} \le \tfrac{8}{5} \cdot 2^j + \tfrac{8}{5} \cdot 2^{m'} \Big \}. \end{aligned} \end{aligned}$$

In view of (2.34), a direct computation shows that

$$\begin{aligned} \Vert \phi ^t_m p_t \Vert _{L^{1}}&\lesssim \exp ( -c_1 4^m ),\\ \Vert \phi ^t_m p_t \Vert _{W^{1,1}}&\lesssim t^{-\frac{1}{2}} \exp ( -c_1 4^m ) \end{aligned}$$

for some $c_1 > 0$. Hence, by the interpolation (2.2), we have

2.37 $$\begin{aligned} \Vert \phi ^t_m p_t \Vert _{W^{s,1}} \lesssim t^{-\frac{s}{2} } \exp ( -c_1 4^m ) \end{aligned}$$

for any $t > 0$ and $0< s < 1$. We point out that (2.37) also holds for any $s \ge 0$. Then, from (2.6), (2.23), (2.35), the fractional Leibniz rule (Lemma 2.8 (i) below), (2.16), Young’s inequality, and (2.37), we have

2.38 $$\begin{aligned} \begin{aligned} \Vert P(t) f \Vert _{W^{s, p}_{\mu }}&\le e^{-t} \sum _{j = 0 }^\infty e^{-\frac{\mu }{p}2^{j\delta }} \Vert \phi _j ( p_t*f )\Vert _{W^{s, p}} \\&\le e^{-t} \sum _{j = 0 }^\infty e^{-\frac{\mu }{p} 2^{j\delta }} \sum _{(m, m')\in \Lambda _{t, j} } \Vert \phi _j \Vert _{W^{s,\infty }} \Vert ( \phi ^t_m p_t) *(\phi _{m'} f )\Vert _{W^{s, p}} \\&\lesssim e^{-t} \sum _{j = 0 }^\infty e^{-\frac{\mu }{p} 2^{j\delta }} \sum _{(m, m')\in \Lambda _{t, j}} \Vert \phi ^t_m p_t \Vert _{W^{s,1}} \Vert \phi _{m'} f \Vert _{L^{p}} \\&\lesssim t^{-\frac{s}{2}} e^{-t} \sum _{j = 0 }^\infty e^{-\frac{\mu }{p} 2^{j\delta }} \sum _{(m, m')\in \Lambda _{t, j}} e^{-c_1 4^m} \Vert f \Vert _{L^{p}_{\mu '}} e^{\frac{\mu '}{p} a_1 2^{m'\delta } }. \end{aligned} \end{aligned}$$

Hence, it remains to estimate

$$\begin{aligned} A_t := \sum _{j = 0}^\infty e^{-\frac{\mu }{p} 2^{j\delta } } \sum _{(m, m')\in \Lambda _{t, j}} e^{-c_1 4^m} e^{\frac{\mu '}{p} a_1 2^{m'\delta } }. \end{aligned}$$

First, we consider the case $0 < t \lesssim 1$. By summing over $m'$ with (2.36), we have

2.39 $$\begin{aligned} A_t \lesssim \sum _{j, m = 0}^\infty e^{-\frac{\mu }{p}2^{j\delta } } e^{-c_1 4^m} e^{\frac{\mu '}{p} c_2 ( 2^{j \delta } + 2^{m\delta } t^\frac{\delta }{2} ) } \lesssim 1, \end{aligned}$$

provided that $\mu > c_2 \mu '$. Next, we consider the case $t \gg 1$. Note that $4^m \sim 2^{m\delta } t^\frac{\delta }{2}$ implies

2.40 $$\begin{aligned} 2^{m\delta }t^\frac{\delta }{2} \sim t^{\frac{\delta }{2-\delta }} \ll t \end{aligned}$$

for $t \gg 1$ since $\delta < 1$. By first summing over $m'$ as in (2.39) and then summing over *m*,

2.41 $$\begin{aligned} A_t \lesssim \sum _{j = 0}^\infty e^{ (-\frac{\mu }{p} + \frac{\mu '}{p} c_2 ) 2^{j \delta } } e^{c_3 t^{\frac{\delta }{2}\frac{2}{2-\delta }}}. \end{aligned}$$

Therefore, the desired bound (2.33) follows from (2.38), (2.39), and (2.41) with (2.40).

$$\square $$

Next, we establish estimates for the linear damped wave operator.

#### Lemma 2.6

Let $s \in {\mathbb {R}}$. Then, there exists $C_0 > 0$ such that

2.42 $$\begin{aligned} \Vert {\mathcal {D}}(t) f\Vert _{H^s_\mu } \lesssim e^{-\frac{t}{4}} \Vert f\Vert _{H^{s-1}_{\mu '}} \end{aligned}$$

and

2.43 $$\begin{aligned} \Vert {\mathcal {S}}(t) f\Vert _{H^s_\mu } \lesssim e^{-\frac{t}{4}} \Vert f\Vert _{H^{s}_{\mu '}} \end{aligned}$$

for $\mu> C_0 \mu ' > 0$. Here, ${\mathcal {D}}(t)$ and ${\mathcal {S}}(t)$ are as in (1.23) and (2.5).

#### Proof

In view of (2.17), write

2.44 $$\begin{aligned} \begin{aligned} \Vert \phi _j {\mathcal {D}}(t) f \Vert _{H^s} = \bigg \Vert \sum _{\ell = 0}^\infty \phi _j {\mathcal {D}}(t) (\phi _\ell f) \bigg \Vert _{H^s}. \end{aligned} \end{aligned}$$

In the following, we only consider $j, \ell \ge 1$ but a similar argument holds for the case $j = 0$ or $\ell = 0$. Thanks to the finite speed of propagation and (2.18), we have

$$\begin{aligned} \mathop {\textrm{supp}}\limits {\mathcal {D}}(t) (\phi _\ell f) \subset \big \{ \tfrac{5}{8} \cdot 2^\ell -t \le |x| \le \tfrac{8}{5} \cdot 2^\ell +t \}. \end{aligned}$$

As a result, $\phi _j {\mathcal {D}}(t) (\phi _\ell f) \ne 0$ only when

2.45 $$\begin{aligned} \tfrac{5}{8}\cdot 2^{j} \le \tfrac{8}{5} \cdot 2^\ell + t \qquad \textrm{and} \qquad \tfrac{5}{8} \cdot 2^{\ell } - t \le \tfrac{8}{5} \cdot 2^j. \end{aligned}$$

From the triangle inequality, we have $\langle \xi \rangle ^s \lesssim \langle \xi _1 \rangle ^{|s|} \langle \xi _2 \rangle ^s$ for $\xi = \xi _1 + \xi _2$. Then, from Young’s inequality on the Fourier side, we have

2.46 $$\begin{aligned} \Vert f g \Vert _{H^s} \lesssim \Vert f\Vert _{{\mathcal {F}}L ^{|s|,1}} \Vert g\Vert _{H^s}, \end{aligned}$$

where the Fourier-Lebesgue norm is defined by

2.47 $$\begin{aligned} \Vert f\Vert _{{\mathcal {F}}L ^{s,1}} = \Vert \langle \xi \rangle ^s \widehat{f}(\xi ) \Vert _{L^1_\xi }. \end{aligned}$$

Note that from (2.16), we have

2.48 $$\begin{aligned} \Vert \phi _j \Vert _{{\mathcal {F}}L^{s,1}} \lesssim _s 1, \end{aligned}$$

uniformly in $$j \in {\mathbb {Z}}_{\ge 0}$$.

Given $j \in {\mathbb {Z}}_{\ge 0}$ and $t \ge 0$, define $\Gamma _{j, t}$ by

$$\begin{aligned} \Gamma _{j, t} = \big \{\ell \in {\mathbb {Z}}_{\ge 0}: \ell \text { satisfies} (2.45)\big \}. \end{aligned}$$

Then, we deduce from (2.44), (2.46) (1.23), and (2.23), we have

2.49 $$\begin{aligned} \begin{aligned} \Vert \phi _j {\mathcal {D}}(t) f \Vert _{H^s}&\lesssim e^{-\frac{t}{2}} \sum _{ \ell \in \Gamma _{j, t} } \Vert \phi _\ell f\Vert _{H^{s-1}} \\&\lesssim e^{-\frac{t}{2}} \bigg (\sum _{ \ell \in \Gamma _{j, t} } e^{\frac{\mu '}{2} 2^{\ell \delta }}\bigg ) \Vert f\Vert _{H^{s-1}_{\mu '}}\\&\lesssim e^{-\frac{t}{2}} e^{c_0\mu '( 2^{j\delta } + t^\delta )} \Vert f\Vert _{H^{s-1}_{\mu '}}, \end{aligned} \end{aligned}$$

where the implicit constant is independent of $j \in {\mathbb {Z}}_{\ge 0}$. Then, from (2.23) and (2.49), we have

$$\begin{aligned} \Vert {\mathcal {D}}(t) f \Vert _{H^s_\mu }&\le \sum _{j = 0}^\infty e^{-\frac{\mu }{2} 2^{j \delta } } \Vert \phi _j {\mathcal {D}}(t) f \Vert _{H^s} \\&\lesssim e^{-\frac{t}{2}} \sum _{j = 0}^\infty e^{-\frac{\mu }{2} 2^{j \delta } } e^{c_0\mu '( 2^{j\delta } + t^\delta )} \Vert f\Vert _{H^{s-1}_{\mu '}} \\&\lesssim e^{-\frac{t}{2}} e^{c_0t^\delta } \Vert f\Vert _{H^{s-1}_{\mu '}} \lesssim e^{-\frac{t}{4}} \Vert f\Vert _{H^{s-1}_{\mu '}} , \end{aligned}$$

provided that $\mu > 2c_0 \mu '$. This proves (2.42). Recalling that

$$\begin{aligned} {\mathcal {S}}(t) = \frac{1}{2}{\mathcal {D}}(t) + e^{-\frac{t}{2}}\cos \Big (t \sqrt{\tfrac{3}{4} -\Delta } \Big ), \end{aligned}$$

the second bound (2.43) follows from an analogous computation. $\square$

#### Remark 2.7

(i) Let $w_\mu$ be as in (2.11). Then, for any $0 < \delta \le 2$ and $\mu > 0$, the Fourier transform of the weight $w_\mu$ is positive. Indeed, it follows from Lemma 5 in [32] on completely monotonic functions that

$$\begin{aligned} w_\mu (x) = \int _0^\infty e^{-t \langle x \rangle ^2} d\alpha _\mu (t) = e^{-t} \int _0^\infty e^{-t |x|^2} d\alpha _\mu (t), \end{aligned}$$

where $\alpha _\mu (t)$ is bounded and non-decreasing and the integral converges for any $x \in {\mathbb {R}}^2$. Hence, as a superposition of the Gaussians $e^{-t |x|^2}$, we conclude that the Fourier transform of the weight $w_\mu$ is positive.

(ii) Let ${\textbf{W}}^{s, 2}_\mu ({\mathbb {R}}^d)$ be the weighted Sobolev space defined in (2.31) with $p = 2$. Then, an analogue of Lemma 2.6 also holds for ${\textbf{W}}^{s, 2}_\mu ({\mathbb {R}}^d)$. Using the positivity of the Fourier transform of the weight $w_{\frac{\mu }{2}}$, we have

$$\begin{aligned} \Vert {\mathcal {D}}(t) f \Vert _{{\textbf{W}}^{s, 2}_\mu }&= \Vert \langle \nabla \rangle ^s {\mathcal {D}}(t) f\Vert _{L^2_\mu } = \Big \Vert \big (\langle \,\cdot \, \rangle ^s \widehat{{\mathcal {D}}(t) f}\big )*\widehat{w}_{\frac{\mu }{2}}(\xi ) \Big \Vert _{L^2_\xi }\\&\lesssim \Big \Vert \big (\langle \,\cdot \, \rangle ^{s-1} |\widehat{ f}|\big )*\widehat{w}_{\frac{\mu }{2}}(\xi ) \Big \Vert _{L^2_\xi } = \Vert f\Vert _{{\textbf{W}}^{s-1, 2}_\mu }. \end{aligned}$$

A similar computation holds for ${\mathcal {S}}(t)$. Note that, unlike Lemma 2.6, there is no loss in the coefficient $\mu$ of the weight.

### Product estimates

We first state the product estimate on ${\mathbb {R}}^d$.

#### Lemma 2.8

(i) Let $s\ge 0$. Suppose that $1<p_j,q_j \le \infty$ and $1 \le r < \infty$ such that $\frac{1}{p_j} + \frac{1}{q_j}= \frac{1}{r}$, $j = 1, 2$. Then, we have

2.50 $$\begin{aligned} \Vert fg \Vert _{W^{s, r}({\mathbb {R}}^d)} \lesssim \Big ( \Vert f \Vert _{L^{p_1}({\mathbb {R}}^d)} \Vert g \Vert _{W^{s, q_1}({\mathbb {R}}^d)} + \Vert f \Vert _{W^{s, p_2}({\mathbb {R}}^d)} \Vert g \Vert _{L^{q_2}({\mathbb {R}}^d)}\Big ). \end{aligned}$$

(ii) Let $s > 0$. Suppose that

- $$ 1< p \le \infty $$ and $$1< q,r < \infty $$,
- $$ 1< p = r \le \infty $$ and $$q = \infty $$, or
- $$1<p = q'\le \infty $$ and $$r=1$$

such that $\frac{1}{r} \le \frac{1}{p} + \frac{1}{q} \le \frac{1}{r} + \frac{s}{d}$ and $q, r' \ge p'$. Then, we have

2.51 $$\begin{aligned} \Vert fg \Vert _{W^{-s, r}({\mathbb {R}}^d) } \lesssim \Vert f\Vert _{W^{-s, p}({\mathbb {R}}^d) } \Vert g \Vert _{W^{s,q}({\mathbb {R}}^d)}. \end{aligned}$$

As for the fractional Leibniz rule (2.50) see [23, 49] for $p_j,q_j < \infty$ and [63, (2.1)] (for the one-dimensional case which can be easily extended to higher dimensions); see also [39]. As for the second estimate (2.51), see [7, 43]. The estimates (2.50) and (2.51) indeed hold for wider ranges of indices; see [7] for a further discussion.

#### Remark 2.9

Note that, given a (nice) subset $K \subset {\mathbb {R}}^d$, the estimates (2.50) and (2.51) also hold on the localized version of Sobolev spaces $W^{s, p}(K)$ defined in (2.3). Given *f* and *g* on *K*, let $\textbf{f}$ and $\textbf{g}$ be extensions onto ${\mathbb {R}}^d$. Then, from (2.3) and (2.50), we have

$$\begin{aligned} \Vert fg \Vert _{W^{s, r}(K)}&\le \Vert \textbf{f}  \textbf{g} \Vert _{W^{s, r}({\mathbb {R}}^d)} \\&\lesssim \Big ( \Vert \textbf{f} \Vert _{L^{p_1}({\mathbb {R}}^d)} \Vert \textbf{g} \Vert _{W^{s, q_1}({\mathbb {R}}^d)} + \Vert \textbf{f} \Vert _{W^{s, p_2}({\mathbb {R}}^d)} \Vert \textbf{g} \Vert _{L^{q_2}({\mathbb {R}}^d)}\Big ). \end{aligned}$$

By taking infima over extensions $\textbf{f}$ and $\textbf{g}$, we then obtain

$$\begin{aligned} \Vert fg \Vert _{W^{s, r}(K)}&\ \lesssim \Big ( \Vert f \Vert _{L^{p_1}(K)} \Vert g \Vert _{W^{s, q_1}(K)} + \Vert f \Vert _{W^{s, p_2}(K)} \Vert g \Vert _{L^{q_2}(K)}\Big ). \end{aligned}$$

A similar comment applies to all the preliminary estimates presented in this section.

Next, we state a bi-parameter version of the fractional Leibniz rule.

#### Lemma 2.10

Let $0 \le s \le 1$, $1 < p_1, p_2 \le \infty$, and $1 \le p < \infty$ with $\frac{1}{p} = \frac{1}{p_1}+ \frac{1}{p_2}$. Then, we have

2.52 $$\begin{aligned} \Vert \langle \nabla _x \rangle ^s \langle \nabla _y \rangle ^s (fg) \Vert _{L^p_{x, y}({\mathbb {R}}^{2d})} \lesssim \Vert \langle \nabla _x \rangle ^s \langle \nabla _y \rangle ^s f \Vert _{L^{p_1}_{x, y}({\mathbb {R}}^{2d})} \Vert \langle \nabla _x \rangle ^s \langle \nabla _y \rangle ^s g \Vert _{L^{p_2}_{x, y}({\mathbb {R}}^{2d})} \end{aligned}$$

for any functions $f= f(x, y)$ and $g= g(x, y)$ on ${\mathbb {R}}^d_x \times {\mathbb {R}}^d_y$.

#### Proof

Let $0 \le s \le 1$ and $1< p < \infty$. Let $f= f(x, y)$ and $g= g(x, y)$ be functions on ${\mathbb {R}}^d_x \times {\mathbb {R}}^d_y$. Then, by the Marcinkiewicz multiplier theorem (Theorem 6.2.4 in [37]), we have

2.53 $$\begin{aligned} \begin{aligned}&\Vert \langle \nabla _x \rangle ^s \langle \nabla _y \rangle ^s (fg) \Vert _{L^p_{x, y}({\mathbb {R}}^{2d})}\\&\quad \lesssim \Vert fg \Vert _{L^p_{x, y}({\mathbb {R}}^{2d})} + \big \Vert |\nabla _x|^s (fg) \big \Vert _{L^p_{x, y}({\mathbb {R}}^{2d})}\\&\qquad + \big \Vert |\nabla _y|^s (fg) \big \Vert _{L^p_{x, y}({\mathbb {R}}^{2d})} + \big \Vert |\nabla _x|^s|\nabla _y|^s (fg) \big \Vert _{L^p_{x, y}({\mathbb {R}}^{2d})}. \end{aligned} \end{aligned}$$

Indeed, noting that

$$\begin{aligned} \text {RHS of }(2.53) \sim \big \Vert (1+ |\nabla _x|^s) (1+ |\nabla _y|^s) (fg) \big \Vert _{L^p_{x, y}({\mathbb {R}}^{2d})}, \end{aligned}$$

the bound (2.53) follows since the multiplier $m(\eta _1, \eta _2)$, $(\eta _1, \eta _2) \in {\mathbb {R}}^d \times {\mathbb {R}}^d$, defined by

$$\begin{aligned} m(\eta _1, \eta _2) = \frac{\langle \eta _1 \rangle ^s\langle \eta _2 \rangle ^s}{(1 + |\eta _1|^s) (1 + |\eta _2|^s)} \end{aligned}$$

is a Marcinkiewicz multiplier.

By Hölder’s inequality and the usual fractional Leibniz rule (Lemma 2.8 (i)), the first three terms on the right-hand side of (2.53) are bounded by the right-hand side of (2.52). As for the last term on the right-hand side of (2.53), it follows from the bi-parameter fractional Leibniz rule ( [62, (61) on p. 295]; see also [63, (3.3)]) that

$$\begin{aligned} \big \Vert |\nabla _x|^s|\nabla _y|^s (fg) \big \Vert _{L^p_{x, y}({\mathbb {R}}^{2d})}&\lesssim \big \Vert |\nabla _x|^s|\nabla _y|^s f \big \Vert _{L^{p_1}_{x, y}({\mathbb {R}}^{2d})} \Vert g \Vert _{L^{p_2}_{x, y}({\mathbb {R}}^{2d})}\\&\quad + \Vert f \big \Vert _{L^{p_1}_{x, y}({\mathbb {R}}^{2d})} \big \Vert |\nabla _x|^s|\nabla _y|^s g \big \Vert _{L^{p_2}_{x, y}({\mathbb {R}}^{2d})}\\&\quad + \big \Vert |\nabla _x|^sf \big \Vert _{L^{p_1}_{x, y}({\mathbb {R}}^{2d})} \big \Vert |\nabla _y|^s g \big \Vert _{L^{p_2}_{x, y}({\mathbb {R}}^{2d})}\\&\quad + \big \Vert |\nabla _y|^sf \big \Vert _{L^{p_1}_{x, y}({\mathbb {R}}^{2d})} \big \Vert |\nabla _x|^s g \big \Vert _{L^{p_2}_{x, y}({\mathbb {R}}^{2d})}, \end{aligned}$$

which is once again bounded by the right-hand side of (2.52). This concludes the proof of Lemma 2.10. $\square$

We extend the product estimates in Lemma 2.8 to weighted Sobolev spaces.

#### Lemma 2.11

(i) Let $s \ge 0$ and $\mu >0$. Suppose that $1<p_j,q_j \le \infty$ and $1 \le r < \infty$ such that $\frac{1}{p_j} + \frac{1}{q_j}= \frac{1}{r}$, $j = 1, 2$. Then, we have

2.54 $$\begin{aligned} \Vert fg \Vert _{W^{s,r}_\mu } \lesssim \Vert f\Vert _{W^{0, p_1}_\mu } \Vert g\Vert _{W^{s,q_1}_{\frac{\mu }{2^\delta } }} + \Vert f\Vert _{W^{s, p_2}_\mu } \Vert g\Vert _{W^{0, q_2}_{ \frac{\mu }{2^\delta } }}. \end{aligned}$$

(ii) Let $s> 0$ and $\mu >0$, and let $1 \le p, q, r \le \infty$ be as in Lemma 2.8 (ii). Then, we have

2.55 $$\begin{aligned} \Vert fg \Vert _{W^{-s,r}_\mu } \lesssim \Vert f\Vert _{W^{-s, p}_\mu } \Vert g\Vert _{W^{s,q}_{\frac{\mu }{2^\delta }}}. \end{aligned}$$

#### Proof

(i) From (2.22) (2.21), the fractional Leibniz rule (Lemma 2.8 (i)), and Hölder’s inequality (in *j*) with $\frac{1}{r} = \frac{1}{p_1} + \frac{1}{q_1}$, (2.20), and (2.11), we have

$$\begin{aligned} \Vert fg \Vert _{W^{s,r}_\mu }&\lesssim \bigg (\sum _{j = 0}^\infty w_\mu (2^j) \Vert \phi _j f g \Vert _{W^{s,r}}^r \bigg )^\frac{1}{r} = \bigg (\sum _{j = 0}^\infty w_\mu (2^j) \Vert \phi _j f \cdot \widetilde{\phi }_j g \Vert _{W^{s,r}}^r \bigg )^\frac{1}{r} \\&\lesssim \bigg (\sum _{j = 0}^\infty w_\mu (2^j) \Vert \phi _j f \Vert _{L^{p_1}} ^r \Vert \widetilde{\phi }_j g \Vert _{W^{s,q_1}}^r \bigg )^\frac{1}{r} \\&\quad + \bigg (\sum _{j = 0}^\infty w_\mu (2^j) \Vert \phi _j f \Vert _{W^{s,p_2}}^r \Vert \widetilde{\phi }_j g \Vert _{L^{q_2}}^r \bigg )^\frac{1}{r} \\&\lesssim \Vert f\Vert _{W^{0, p_1}_\mu } \Vert g\Vert _{W^{s,q_1}_{\frac{\mu }{2^\delta } }} + \Vert f\Vert _{W^{s, p_2}_\mu } \Vert g\Vert _{W^{0, q_2}_{ \frac{\mu }{2^\delta } }}, \end{aligned}$$

which yields (2.54).

(ii) By Lemma 2.8 (ii), we have

$$\begin{aligned} \Vert \phi _j f \cdot \widetilde{\phi }_j g \Vert _{W^{-s,r}} \lesssim \Vert \phi _j f \Vert _{W^{-s,p}} \Vert \widetilde{\phi }_j g \Vert _{W^{s,q}}. \end{aligned}$$

Then, (2.55) follows from applying Hölder’s inequality as in Part (i). $\square$

### Tools from stochastic analysis

In the following, we review some basic facts on the Hermite polynomials and the Wiener chaos estimate. See, for example, [52, 65].

We define the *k*th Hermite polynomials $H_k(x; \sigma )$ with variance $\sigma$ via the following generating function:

$$\begin{aligned} e^{tx - \frac{1}{2}\sigma t^2} = \sum _{k = 0}^\infty \frac{t^k}{k!} H_k(x;\sigma ) \end{aligned}$$

for $t, x \in {\mathbb {R}}$ and $\sigma > 0$. When $\sigma = 1$, we set $H_k(x) = H_k(x; 1)$. Then, we have

2.56 $$\begin{aligned} H_k(x ; \sigma ) = \sigma ^{\frac{k}{2}}H_k(\sigma ^{-\frac{1}{2}}x ). \end{aligned}$$

It is well known that $\big \{ H_k /\sqrt{k!}\big \}_{k \in {\mathbb {Z}}_{\ge 0}}$ form an orthonormal basis of $L^2({\mathbb {R}}; \frac{1}{\sqrt{2\pi }} e^{-x^2/2}dx)$. The following identity:

$$\begin{aligned} H_k(x+y)&= \sum _{\ell = 0}^k \begin{pmatrix} k \\ \ell \end{pmatrix} x^{k - \ell } H_\ell (y), \end{aligned}$$

which, together with (2.56), yields

2.57 $$\begin{aligned} \begin{aligned} H_k(x+y; \sigma )&= \sigma ^\frac{k}{2}\sum _{\ell = 0}^k \begin{pmatrix} k \\ \ell \end{pmatrix} \sigma ^{-\frac{k-\ell }{2}} x^{k - \ell } H_\ell (\sigma ^{-\frac{1}{2}} y)\\&= \sum _{\ell = 0}^k \begin{pmatrix} k \\ \ell \end{pmatrix} x^{k - \ell } H_\ell (y; \sigma ). \end{aligned} \end{aligned}$$

Let $(H, B, \mu )$ be an abstract Wiener space. Namely, $\mu$ is a Gaussian measure on a separable Banach space *B* with $H \subset B$ as its Cameron-Martin space. Given a complete orthonormal system $\{e_j \}_{ j \in {\mathbb {N}}} \subset B^*$ of $H^* = H$, we define a polynomial chaos of order *k* to be an element of the form $\prod _{j = 1}^\infty H_{k_j}(\langle x, e_j \rangle )$, where $x \in B$, $k_j \ne 0$ for only finitely many *j*’s, $k= \sum _{j = 1}^\infty k_j$, $H_{k_j}$ is the Hermite polynomial of degree $k_j$, and $\langle \cdot , \cdot \rangle = \phantom {|}_B \langle \cdot , \cdot \rangle _{B^*}$ denotes the *B*–$B^*$ duality pairing. We then denote the closure of polynomial chaoses of order *k* under $L^2(B, \mu )$ by ${\mathcal {H}}_k$. The elements in ${\mathcal {H}}_k$ are called homogeneous Wiener chaoses of order *k*. Then, we have the following Ito-Wiener decomposition:

$$\begin{aligned} L^2(B, \mu ) = \bigoplus _{k = 0}^\infty {\mathcal {H}}_k. \end{aligned}$$

See Theorem 1.1.1 in [65]. See also [10, 48]. We also set

$$\begin{aligned} {\mathcal {H}}_{\le k} = \bigoplus _{j = 0}^k {\mathcal {H}}_j\end{aligned}$$

for $$k \in {\mathbb {N}}$$.

We now state the Wiener chaos estimate, which is a consequence of Nelson’s hypercontractivity [64]. See, for example, [88, Theorem I.22]. See also [90, Proposition 2.4].

#### Lemma 2.12

Let $k \in {\mathbb {Z}}_{\ge 0}$. Then, we have

$$\begin{aligned} \Big ( {\mathbb {E}}\big [|X|^p \big ]\Big )^{\frac{1}{p}} \le (p-1)^\frac{k}{2} \Big ( {\mathbb {E}}\big [|X|^2\big ] \Big )^{\frac{1}{2}} \end{aligned}$$

for any random variable $X \in {\mathcal {H}}_k$ and any $2 \le p < \infty$.

Next, we recall the following orthogonal property of Wick powers; see [65, Lemma 1.1.1].

#### Lemma 2.13

Let $Y_1, Y_2$ be two real-valued, mean-zero, and jointly Gaussian random variables with variances $\sigma _1 = {\mathbb {E}}[ Y_1^2] > 0$ and $\sigma _2 = {\mathbb {E}}[ Y_2^2] > 0$. Then, for $k, m \in {\mathbb {N}}\cup \{0\}$, we have

$$\begin{aligned} {\mathbb {E}}\big [ H_k(Y_1 ; \sigma _1) H_m(Y_2; \sigma _2)\big ] = {\textbf{1}}_{k=m} \cdot k! \big ({\mathbb {E}}[ Y_1 Y_2] \big )^k. \end{aligned}$$

The following lemma allows us to compute the regularity of a stochastic term by testing it against a test function. See [91, Proposition A.3.3] for the proof.

#### Lemma 2.14

Let $X \in {\mathcal {H}}_{\le k}$ for some $k \in {\mathbb {N}}$.^21^ Let $R \ge 1$. Suppose that there exist $\sigma \in {\mathbb {R}}$ and $2\le q < \infty$ such that

$$\begin{aligned} {\mathbb {E}}\Big [ \big | \langle X, \varphi \rangle \big |^2 \Big ] \le A^2 \Vert \varphi \Vert _{W^{\sigma , q'}}^2 \end{aligned}$$

for any test function $\varphi \in C^\infty _c$ supported on a ball *B* of radius 1 with $B \subset B_{2R}$, where $\frac{1}{q'}+ \frac{1}{q} =1$. Then, given any small $\varepsilon >0$ and any finite $p\ge \frac{4}{\varepsilon }$, we have

$$\begin{aligned} {\mathbb {E}}\Big [ \Vert X\Vert _{W^{-\frac{2}{q} -\sigma - \varepsilon ,\infty } (B_R) }^p \Big ] \lesssim _{\sigma , q, R } A^p. \end{aligned}$$

$\bullet$ **Wasserstein-1 metric and weak convergence. **

Let (*X*, *d*) be a Polish space (separable complete metric space). Then, the Wasserstein-1 metric for two probability measures $\mu , \nu$ on *X* is defined by

2.58 $$\begin{aligned} d_{\textrm{Wass}}(\mu , \nu ) = \inf _{{\mathfrak {p}}\in \Pi (\mu , \nu )} \int _{X \times X} d (x,y) d{\mathfrak {p}}(x, y). \end{aligned}$$

Here, $\Pi (\mu , \nu )$ is the set of probability measures ${\mathfrak {p}}$ on $X \times X$ whose first and second marginals are given by $\mu$ and $\nu$, namely,

2.59 $$\begin{aligned} \int _{y \in X} d{\mathfrak {p}}(x, y) = d\mu (x) \qquad \text {and} \qquad \int _{x \in X} d{\mathfrak {p}}(x, y) = d\nu (y). \end{aligned}$$

The Wasserstein-1 metric is also known as the Kantorovich-Rubinstein distance; see the Kantorovich-Rubinstein theorem ( [97, Theorem 1.14]) which provides the dual characterization of (2.58). We point out that the infimum in (2.58) is indeed attained; see [97, Theorem 1.3].

In general, convergence in the Wasserstein-1 metric is stronger than weak convergence. We recall the following characterization of convergence in the Wasserstein-1 metric; see [97, Theorem 7.12].

#### Lemma 2.15

Let (*X*, *d*) be a Polish space. Given a sequence of probability measures $\{\mu _n\}_{n \in {\mathbb {N}}}$ on *X* and a probability measure $\mu$ on *X*, the sequence $\{\mu _n\}_{n \in {\mathbb {N}}}$ converges to $\mu$ in the Wasserstein-1 metric as $n \rightarrow \infty$ if and only if

(i) $\mu _n$ converges weakly to $\mu$ as $n \rightarrow \infty$, and
(ii) for some (and thus for any) $x_0\in X$, we have
  2.60 $$\begin{aligned} \lim _{R\rightarrow \infty } \limsup _{n\rightarrow \infty } \int _{d(x, x_0) \ge R} d(x, x_0) d\mu _n(x) = 0. \end{aligned}$$

#### Remark 2.16

When the metric *d* on *X* is bounded, the condition (2.60) trivially holds, and thus convergence in the Wasserstein-1 metric coincides with weak convergence. See also Remark 7.13 (iii) in [97].

Lastly, we recall the Prokhorov theorem and the Skorokhod representation theorem. We first recall the definition of tightness.

#### Definition 2.17

Let ${\mathcal {J}}$ be a nonempty index set. A family $\{ \mu _n \}_{n \in {\mathcal {J}}}$ of probability measures on a metric space ${\mathcal {M}}$ is said to be tight if, for every $\varepsilon > 0$, there exists a compact set $K_\varepsilon \subset {\mathcal {M}}$ such that $\sup _{n\in {\mathcal {J}}}\mu _n(K_\varepsilon ^c) \le \varepsilon$. We say that $\{ \mu _n \}_{n \in {\mathcal {J}}}$ is relatively compact, if every sequence in $\{ \mu _n \}_{n \in {\mathcal {J}}}$ contains a weakly convergent subsequence.

We now recall the following Prokhorov theorem from [9].

#### Lemma 2.18

(Prokhorov theorem). If a sequence of probability measures on a metric space ${\mathcal {M}}$ is tight, then it is relatively compact. If in addition, ${\mathcal {M}}$ is separable and complete, then relative compactness is equivalent to tightness.

Lastly, we recall the following Skorokhod representation theorem from [5, Chapter 31].

#### Lemma 2.19

(Skorokhod representation theorem). Let ${\mathcal {M}}$ be a complete separable metric space (i.e. a Polish space). Suppose that a sequence $\{\mu _n\}_{n\in {\mathbb {N}}}$ of probability measures on ${\mathcal {M}}$ converges weakly to a probability measure $\mu$ as $n \rightarrow \infty$. Then, there exist a probability space $(\widetilde{\Omega }, \widetilde{\mathcal {F}}, \widetilde{\mathbb {P}})$, and random variables $X_n, X:\widetilde{\Omega }\rightarrow {\mathcal {M}}$ such that

$$\begin{aligned} {{\,\textrm{Law}\,}}( X_n) = \mu _n \qquad \text {and}\qquad {{\,\textrm{Law}\,}}(X) = \mu , \end{aligned}$$

and $X_n$ converges $\widetilde{\mathbb {P}}$-almost surely to *X* as $n\rightarrow \infty$.

## Coming Down from Infinity

In this section, we study the following stochastic nonlinear heat equation (SNLH) on ${\mathbb {R}}^2$:

3.1 $$\begin{aligned} {\left\{ \begin{array}{ll} \partial _tX +(1 -\Delta ) X + :\!X^k\!: \, = \sqrt{2}\xi \\ X|_{t=0} = X_0, \end{array}\right. } \end{aligned}$$

where $k \in 2{\mathbb {N}}+ 1$ and $\xi$ is a Gaussian space-time white noise on ${\mathbb {R}}_+\times {\mathbb {R}}^2$. Let *Z* denote the solution to the following linear equation:

3.2 $$\begin{aligned} {\left\{ \begin{array}{ll} \partial _tZ + (1-\Delta ) Z = \sqrt{2}\xi \\ Z|_{t = 0} = 0. \end{array}\right. } \end{aligned}$$

Namely, *Z* is the stochastic convolution, formally given by

$$\begin{aligned} Z(t) = \sqrt{2}\int _0^t P(t - t') \xi (dt'), \end{aligned}$$

where $P(t)= e^{t(\Delta -1)}$ is the linear heat propagator. Then, with the first order expansion

3.3 $$\begin{aligned} X = Y + Z, \end{aligned}$$

the remainder term $Y = X-Z$ satisfies

3.4 $$\begin{aligned} {\left\{ \begin{array}{ll} \partial _tY + (1-\Delta ) Y + \sum _{\ell =0}^k {k\atopwithdelims ()\ell } :\!Z^\ell \!: \,Y^{k-\ell } = 0\\ Y|_{t=0} = X_0, \end{array}\right. } \end{aligned}$$

where $:\!Z^\ell \!:$ denotes the Wick-renormalized power of *Z*. See Section 5 in [60] for a further discussion. We say that *X* is a solution to (3.1) if it satisfies (3.3) together with (3.2) and (3.4). Our main goal in this section is to establish coming down from infinity for a solution *Y* to (3.4) in weighted Sobolev spaces of positive regularities (Proposition 3.3), which will play a crucial role in Sect. 4.2. For this purpose, we consider

3.5 $$\begin{aligned} {\left\{ \begin{array}{ll} \partial _tY + (1-\Delta ) Y + \sum _{\ell =0}^k Z^{(\ell )}Y^{k-\ell } = 0\\ Y|_{t=0} = X_0, \end{array}\right. } \end{aligned}$$

where $Z^{(0)}= 1$ and $Z^{(\ell )}$, $\ell = 1, \dots , k$, are given space-time distributions. We first establish coming down from infinity for a solution *Y* to (3.5) in weighted Lebesgue spaces in Sect. 3.1. In Sect. 3.2, we then establish coming down from infinity in weighted Sobolev spaces of positive regularities (Proposition 3.3). As a corollary to the coming down from infinity in weighted Lebesgue spaces, we construct a limiting Gibbs measure $\vec {\rho }_\infty$ on the plane ${\mathbb {R}}^2$ (Theorem 1.2 (i)); see Sect. 3.3. We point out that while the construction of a limiting Gibbs measure on ${\mathbb {R}}^2$ requires only the coming down from infinity in weighted Lebesgue spaces (Proposition 3.1), the coming down from infinity in weighted Sobolev spaces of positive regularity (Proposition 3.3) plays an essential role in the construction of the enhanced Gibbs measure as well as global-in-time dynamics on ${\mathbb {R}}^2$ presented in Sect 4.

In the following discussion, the regularity of the initial data $X_0$ in (3.5) does not play an important role (which is precisely the point of coming down from infinity) as long as a solution $X = Y+Z$ exists. For this reason, we do not precisely state the regularity of the initial data $X_0$ in Propositions 3.1 and 3.3. For our application to (3.1) (and its *L*-periodic counterpart (3.42)), it suffices to take $X_0 \in B^{-\varepsilon , \mu }_{p_0, \infty }({\mathbb {R}}^2)$ for some $\varepsilon > 0$, $p_0 \gg 1$, and $\mu > 0$ so that a global solution is guaranteed to exist; see [60]. In particular, the initial data distributed by the *L*-periodic $\Phi ^{k+1}_2$-measure $\rho _L$ in (1.21) almost surely satisfies this regularity condition.

### Coming down from infinity on weighted lebesgue spaces

In this subsection, our main goal is to prove the following coming down from infinity for a solution *Y* to (3.5) in the weighted Lebesgue space $L^p_\mu ({\mathbb {R}}^2)$. As compared to the previous work [96] on ${\mathbb {T}}^2$, we need to proceed with more care, using a decomposition of the physical space into unit cubes and the Littlewood-Paley decomposition; see (3.16)-(3.26).

#### Proposition 3.1

Let $k \in 2{\mathbb {N}}+ 1$ and $T> 0$. Let *Y* be a solution to (3.5) on the time interval [0, *T*]. Then, given any finite $p\ge 1$ and $\mu > 0$, there exist $\lambda > 1$, small $\varepsilon > 0$, finite $p_0 \gg 1$, and $0< \mu _0 < \mu$ such that

3.6 $$\begin{aligned} \Vert Y(t) \Vert _{L^p_\mu } \le C \bigg \{ t^{-\frac{1}{(\lambda -1)p}} \vee \sum _{\ell = 0}^k \Vert Z^{(\ell )}(t)\Vert _{B^{-\varepsilon , \mu _0}_{p_0, p_0}}^{\frac{p_0}{\lambda p}} \bigg \} \end{aligned}$$

for $0 < t \le T$, where the constant *C* is independent of the initial condition $X_0$ in (3.5) and $T > 0$.

For the later use, we set

3.7 $$\begin{aligned} C_{{\bar{Z}}, p, \mu }(t) = \sup _{0 \le t' \le t} \sum _{\ell = 0}^k \Vert Z^{(\ell )}(t')\Vert _{B^{-\varepsilon , \mu _0}_{p_0, p_0}}^{\frac{p_0}{\lambda p}}. \end{aligned}$$

Before proceeding to the proof of Proposition 3.1, we first recall the following key lemma; see Lemma 3.8 in [96].

#### Lemma 3.2

Let $F: [0, T] \rightarrow [0, \infty )$ be a differentiable function. Suppose that there exist $\lambda > 1$ and $c_1, c_2 > 0$ such that

3.8 $$\begin{aligned} \partial _tF(t) + c_1 F^\lambda (t) \le c_2 \end{aligned}$$

for any $0 \le t \le T$. Then, we have

$$\begin{aligned} F(t)&\le \frac{F(0)}{\big (1 + t F^{\lambda -1}(0)(\lambda -1)\frac{c_1}{2}\big )^\frac{1}{\lambda -1}} \vee \bigg (\frac{2c_2}{c_1}\bigg )^\frac{1}{\lambda }\\&\le \Bigg \{t^{-\frac{1}{\lambda -1}}\bigg ((\lambda -1) \frac{c_1}{2}\bigg )^{-\frac{1}{\lambda -1}}\Bigg \} \vee \bigg (\frac{2c_2}{c_1}\bigg )^\frac{1}{\lambda }\end{aligned}$$

for any $$0 < t \le T$$.

Lemma 3.2 shows that in order to establish coming down from infinity, it suffices to establish a bound of the form (3.8).

We now present the proof of Proposition 3.1.

#### Proof of Proposition 3.1

By Hölder’s inequality, it suffices to consider $p \in 2{\mathbb {N}}$. Fix $p \in 2{\mathbb {N}}$. Then, using the equation (3.5), we have

3.9 $$\begin{aligned} \begin{aligned} \frac{1}{p} \partial _t\Vert Y(t) \Vert _{L^p_\mu }^p&= \langle \partial _tY(t), Y^{p-1}(t) w_\mu \rangle _{L^2_x} \\&= \bigg \langle \Delta Y(t) - Y(t) - \sum _{\ell =0}^k Z^{(\ell )}(t) Y^{k-\ell }(t), Y^{p-1}(t) w_\mu \bigg \rangle _{L^2_x}. \end{aligned} \end{aligned}$$

For simplicity of notation, we will drop the time dependence in the following computations.

From (2.11) with $0< \delta < 1$, we have

3.10 $$\begin{aligned} \begin{aligned} \langle \Delta Y, Y^{p-1} w_\mu \rangle _{L^2}&= -\int _{{\mathbb {R}}^2} \nabla Y \cdot \nabla ( Y^{p-1} w_\mu ) dx \\&=- (p-1)\int _{{\mathbb {R}}^2} |\nabla Y|^2 Y^{p-2} w_\mu dx \\&+ \mu \delta \int _{{\mathbb {R}}^2} (\nabla Y \cdot x) Y^{p-1} \langle x \rangle ^{\delta -2} w_\mu dx \\&=: -(p-1) K_t + B_0. \end{aligned} \end{aligned}$$

Since *p* is even, we have

3.11 $$\begin{aligned} \begin{aligned} \langle -Y, Y^{p-1} w_\mu \rangle _{L^2}&= - \int _{{\mathbb {R}}^2} Y^p w_\mu dx \le 0. \end{aligned} \end{aligned}$$

As for the contribution from the last term in (3.9), recalling that $Z^{(0)}= 1$, we write

3.12 $$\begin{aligned} \begin{aligned} \bigg \langle&- \sum _{\ell =0}^k Z^{(\ell )} Y^{k-\ell }, Y^{p-1} w_\mu \bigg \rangle _{L^2} \\&= -\int _{{\mathbb {R}}^2} Y^{p+k-1} w_\mu dx - \sum _{\ell = 1}^{k} \int _{{\mathbb {R}}^2} Y^{p + k - \ell -1 } Z^{(\ell )} w_\mu dx\\&=: - L_t - \sum _{\ell = 1}^{k} B_\ell . \end{aligned} \end{aligned}$$

Since $p \in 2{\mathbb {N}}$ and $k \in 2{\mathbb {N}}+1$, we have $K_t$ and $L_t$ are non-negative. In the following, we control the terms $B_\ell$, $\ell = 0, \dots , k$, by these non-negative terms. For this reason, we set

3.13 $$\begin{aligned} M_t = K_t + L_t. \end{aligned}$$

We first treat $B_0$ in (3.10). In view of the fast decay of the weight $w_\mu$, it follows from Hölder’s inequality that

3.14 $$\begin{aligned} \Vert Y\Vert _{L^p_\mu }^p \le C_{\mu , \delta } \Vert Y\Vert _{L^{p+k-1}_\mu }^p = C_{\mu , \delta } L_t^\frac{p}{p+k-1} \le C_{\mu , \delta } M_t^\frac{p}{p+k-1}. \end{aligned}$$

Then, by recalling $0< \delta < 1$ and applying Cauchy-Schwarz’s inequality and Young’s inequality with (3.14) and (3.13), we have

3.15 $$\begin{aligned} \begin{aligned} |B_0|&\le \mu \delta \int _{{\mathbb {R}}^2} |\nabla Y| \cdot |Y|^{p-1} \langle x \rangle ^{\delta -1} w_\mu dx \\&\le C_{\mu , \delta } \int _{{\mathbb {R}}^2} |\nabla Y| |Y|^{\frac{p-2}{2}} \cdot |Y|^{\frac{p}{2}} w_\mu dx \\&\le C_{\mu , \delta } K_t^{\frac{1}{2}} \Vert Y\Vert _{L^p_\mu }^{\frac{p}{2}} \le \tfrac{1}{100} K_t + C'_{\mu , \delta } \Vert Y\Vert _{L^p_\mu }^{p} \\&\le \tfrac{1}{100} M_t + C''_{ \mu , \delta }. \end{aligned} \end{aligned}$$

Next, we consider $$B_\ell $$, $$\ell = 1, \dots , k$$. Let $$Q= \big [-\frac{1}{2}, \frac{1}{2}\big )^2$$ be the unit cube in $${\mathbb {R}}^2$$ and set $$Q_n = n + Q$$. Fix small $$\varepsilon > 0$$ and $$ r > 1$$ sufficiently close to 1. Then, we have

3.16 $$\begin{aligned} \begin{aligned} |B_\ell |&= \bigg |\int _{{\mathbb {R}}^2} Y^{p + k - \ell -1 } Z^{(\ell )} w_\mu dx\bigg | \le \sum _{n \in {\mathbb {Z}}^2} \bigg | \int _{Q_n} Y^{p + k - \ell -1 } Z^{(\ell )} w_\mu dx\bigg |\\&\le \sum _{n \in {\mathbb {Z}}^2} \Vert Y^{p + k - \ell -1 }w_\mu \Vert _{W^{\varepsilon , r}(Q_n)} \Vert Z^{(\ell )}\Vert _{W^{-\varepsilon , r'}(Q_n)}, \end{aligned} \end{aligned}$$

where $$\frac{1}{r} + \frac{1}{r'} = 1$$.

From (2.26) and the Littlewood-Paley decomposition with the uniform boundedness on $L^r({\mathbb {R}}^2)$ of the Littlewood-Paley projector ${\textbf{Q}}_j$, we have

3.17 $$\begin{aligned} \begin{aligned}&\Vert Y^{p + k - \ell -1 } w_\mu \Vert _{W^{\varepsilon , r}(Q_n)} \lesssim \Vert Y^{p + k - \ell -1 }w_\mu \Vert _{B^{2\varepsilon }_{r, \infty }(Q_n)} \\&= \sup _{j \in {\mathbb {Z}}_{\ge 0}}2^{2\varepsilon j} \Vert {\textbf{Q}}_j (Y^{p + k - \ell -1 }w_\mu )\Vert _{L^r(Q_n)} \\&\le \sup _{j \in {\mathbb {Z}}_{\ge 0}} 2^{2\varepsilon j} \sum _{j_1, j_2 = 0}^\infty \big \Vert {\textbf{Q}}_j \big ({\textbf{Q}}_{j_1}(Y^{p + k - \ell -1 }){\textbf{Q}}_{j_2}w_\mu \big )\big \Vert _{L^r(Q_n)} \\&\lesssim \sum _{j_1 \ge j_2 +2} 2^{2\varepsilon j_1} \Vert {\textbf{Q}}_{j_1}(Y^{p + k - \ell -1 }){\textbf{Q}}_{j_2}w_\mu \Vert _{L^r(Q_n)} \\&+ \sum _{j_1 < j_2 +2} 2^{2\varepsilon j_2} \Vert {\textbf{Q}}_{j_1}(Y^{p + k - \ell -1 }){\textbf{Q}}_{j_2}w_\mu \Vert _{L^r(Q_n)} \\&=: \text{ I } + \text {II} \end{aligned} \end{aligned}$$

As for the first term $\hspace{0.5mm}\text {I}\hspace{0.5mm}$, it follows from (2.14) and (2.13) that

3.18 $$\begin{aligned} \begin{aligned} \hspace{0.5mm}\text {I}\hspace{0.5mm}&\lesssim \sum _{j_1 \ge j_2 +2} 2^{2\varepsilon j_1} 2^{-\frac{1}{2} j_2}w_\mu (n) \Vert {\textbf{Q}}_{j_1}(Y^{p + k - \ell -1 })\Vert _{L^r(Q_n)} \\&\lesssim w_\mu (n) \Vert Y^{p + k - \ell -1 }\Vert _{W^{3\varepsilon , r}(Q_n)}. \end{aligned} \end{aligned}$$

Similarly, we have

3.19 $$\begin{aligned} \begin{aligned} \text {II}&\lesssim \sum _{j_1 < j_2 +2} 2^{(2\varepsilon -\frac{1}{2}) j_2}w_\mu (n) \Vert {\textbf{Q}}_{j_1}(Y^{p + k - \ell -1 })\Vert _{L^r(Q_n)} \\&\lesssim w_\mu (n) \Vert Y^{p + k - \ell -1 }\Vert _{W^{3 \varepsilon , r}(Q_n)}. \end{aligned} \end{aligned}$$

In the following, we estimate $\Vert Y^{p + k - \ell -1 }\Vert _{W^{3\varepsilon , r}(Q_n)}$. Define $M_t(Q_n)$ by

3.20 $$\begin{aligned} M_t(Q_n) = \int _{Q_n} |\nabla Y|^2 Y^{p-2} + Y^{p+k-1} dx . \end{aligned}$$

By the interpolation (2.2), we have

3.21 $$\begin{aligned} \begin{aligned}&\Vert Y^{p + k - \ell -1 } \Vert _{W^{3\varepsilon , r}(Q_n)} \lesssim \Vert Y^{p + k - \ell -1 }\Vert _{L^r(Q_n)}^{1-3\varepsilon } \Vert Y^{p + k - \ell -1 }\Vert _{W^{1, r}(Q_n)}^{3\varepsilon }\\&\quad \sim \Vert Y^{p + k - \ell -1 }\Vert _{L^r(Q_n)}^{1-3\varepsilon } \Vert \nabla ( Y^{p + k - \ell -1 })\Vert _{L^r(Q_n)}^{3\varepsilon } + \Vert Y^{p + k - \ell -1 }\Vert _{L^r(Q_n)}. \end{aligned} \end{aligned}$$

By Hölder’s inequality and by choosing $r > 1$ sufficiently close to 1, we have

3.22 $$\begin{aligned} \Vert Y^{p + k - \ell -1 }\Vert _{L^r(Q_n)} = \Vert Y\Vert _{L^{(p+k-\ell -1)r}(Q_n)}^{p+k-\ell -1} \le M_t(Q_n)^\frac{p+k-\ell -1}{p+k-1} \end{aligned}$$

for any $\ell = 1, \dots , k$. On the other hand, by Hölder’s inequality, we have

3.23 $$\begin{aligned} \begin{aligned} \Vert \nabla ( Y^{p + k - \ell -1 })\Vert _{L^r(Q_n)}&\sim \Vert \nabla Y \cdot |Y|^\frac{p-2}{2} Y^{\frac{p}{2} + k - \ell -1 }\Vert _{L^r(Q_n)}\\&\le M(Q_n)^\frac{1}{2} \Vert Y^{\frac{p}{2} + k - \ell -1 }\Vert _{L^\frac{2r}{2-r}(Q_n)}\\&\le M(Q_n)^\frac{1}{2} \big \Vert |Y|^\frac{p}{2} \big \Vert _{L^q(Q_n)}^{1 + \frac{2}{p} (k - \ell - 1)}, \end{aligned} \end{aligned}$$

where $q = \frac{2}{p} \cdot \frac{2r}{2-r} \big (\frac{p}{2} + k - \ell - 1\big )$. By Sobolev’s inequality,^22^ the interpolation (2.2), and Young’s inequality followed by Hölder’s inequality, we have

3.24 $$\begin{aligned} \begin{aligned} \big \Vert |Y|^\frac{p}{2} \big \Vert _{L^q(Q_n)}&\lesssim \big \Vert |Y|^\frac{p}{2} \big \Vert _{L^2(Q_n)} + \big \Vert |Y|^\frac{p-2}{2} \nabla Y\big \Vert _{L^2(Q_n)}\\&\le M_t(Q_n)^{\frac{1}{2} \cdot \frac{p}{p+k-1}}+ M_t(Q_n)^\frac{1}{2}. \end{aligned} \end{aligned}$$

Hence, from (3.21), (3.22), (3.23), and (3.24), we obtain

3.25 $$\begin{aligned}&\Vert Y^{p + k - \ell -1 } \Vert _{W^{3\varepsilon , r}(Q_n)} \lesssim M_t(Q_n)^{1-\theta } + 1 \end{aligned}$$

for some $\theta > 0$, provided that $\varepsilon > 0$ is sufficiently small, where the implicit constant is independent of $\ell = 1, \dots , k$.

Hence, putting (3.16), (3.17), (3.18), (3.19), and (3.25) together and applying Young’s inequality with (2.26), we have

3.26 $$\begin{aligned} \begin{aligned} |B_\ell |&\lesssim \sum _{n \in {\mathbb {Z}}^2} w_\mu (n) \Big (M_t(Q_n)^{1-\theta } + 1\Big ) \Vert Z^{(\ell )}\Vert _{W^{-\varepsilon , r'}(Q_n)}\\&\le \varepsilon _0 \sum _{n \in {\mathbb {Z}}^2} w_\mu (n) M_t(Q_n) + C_{\varepsilon _0} \sum _{n \in {\mathbb {Z}}^2} w_\mu (n) \Vert Z^{(\ell )}\Vert _{W^{-\varepsilon , r'}(Q_n)}^\frac{1}{\theta }+C_1\\&\lesssim \varepsilon _0 M_t + C_{\varepsilon _0} \Vert Z^{(\ell )}\Vert _{B^{-\frac{\varepsilon }{2}, \mu _0}_{p_0, p_0}}^{p_0} +C_2 \end{aligned} \end{aligned}$$

for some small $\varepsilon _0>0$, finite $p_0 \gg 1$, and $\mu _0 < \mu$. Here, the last step follows from (3.13), (3.20), and (2.13) for the first term, while it follows from (2.25) and (2.13) for the second term.

Therefore, from (3.9), (3.10), (3.11), (3.12), (3.15), and (3.26), we obtain

$$\begin{aligned} \frac{1}{p} \partial _t\Vert Y(t) \Vert _{L^p_\mu }^p + c_0 M_t \lesssim \sum _{\ell = 0}^k \Vert Z^{(\ell )}(t)\Vert _{B^{-\frac{\varepsilon }{2}, \mu _0}_{p_0, p_0}}^{p_0}. \end{aligned}$$

Finally, in view of (3.14), there exists $\lambda > 1$ such that

3.27 $$\begin{aligned} \begin{aligned} \frac{1}{p} \partial _t\Vert Y(t) \Vert _{L^p_\mu }^p + c_1 \Vert Y(t) \Vert _{L^p_\mu }^{\lambda p } \lesssim \sum _{\ell = 0}^k \Vert Z^{(\ell )}(t)\Vert _{B^{-\frac{\varepsilon }{2}, \mu _0}_{p_0, p_0}}^{p_0} \end{aligned} \end{aligned}$$

for any $0 \le t \le T$. Finally, the desired bound (3.6) follows from (3.27) and Lemma 3.2. This concludes the proof of Proposition 3.1. $\square$

### Coming down from infinity on weighted Sobolev spaces of positive regularities

In this subsection, we establish the following coming down from infinity for a solution *Y* to (3.5) in a weighted Sobolev space of positive regularity.

#### Proposition 3.3

Let $k \in 2{\mathbb {N}}+ 1$ and $T> 0$. Let *Y* be a solution to (3.5) on the time interval [0, *T*]. Then, given any $0< s < 1$, finite $p\ge 1$, and $\mu > 0$, there exist $\lambda , \lambda ' > 1$, small $\theta > 0$, finite $q_0, q \gg 1$, and $0< \mu ', \mu _1 < \mu$ such that

3.28 $$\begin{aligned} \begin{aligned} \Vert Y(t)\Vert _{W^{s, p}_\mu }&\le C(T) \bigg \{ t^{-\frac{s}{2}} \Big ( t^{-\frac{1}{( \lambda ' - 1) p} } \vee C_{{\bar{Z}}, p, \mu '}(t)\Big )\\&+ t^{-\frac{1}{\lambda - 1} }\vee \big (C_{{\bar{Z}}, q_0, \mu }(t)\big )^{q_0} + Q_{{\bar{Z}}, \theta , q, \mu _1} (t) + 1 \bigg \} \end{aligned} \end{aligned}$$

for $0 < t \le T$, where the constant *C*(*T*) is independent of the initial condition $X_0$ in (3.5). Here, $C_{{\bar{Z}}, p, \mu }$ is as in (3.7) and

3.29 $$\begin{aligned} Q_{{\bar{Z}}, \theta , q, \mu _1} (t) = \sup _{0 \le t' \le t} \sum _{\ell =0}^k \Vert Z^{(\ell )} (t') \Vert _{B^{-\frac{\theta }{2}, \mu _1}_{q, q}}^q. \end{aligned}$$

The proof of Proposition 3.3 is based on a Gronwall-type argument, utilizing the already established coming down from infinity in weighted Lebesgue spaces (Proposition 3.1). While the idea is straightforward, an actual implementation requires careful analysis via a spatial decomposition (analogous to the proof of Lemma 2.5), for which we find our definition (2.22) of the weighted Sobolev spaces $W^{s, p}_\mu ({\mathbb {R}}^2)$ more convenient (than ${\textbf{W}}^{s, p}_\mu ({\mathbb {R}}^2)$ defined in (2.31)).

#### Proof

Fix $0< r < t$. Then, from (3.5) and (2.23), we have

3.30 $$\begin{aligned} \begin{aligned}&\Vert Y (t) \Vert _{W^{s,p }_\mu } \le \Vert P(t-r) Y(r) \Vert _{W^{s, p }_\mu } + \sum _{\ell =0}^k \int _{r}^t\Vert P(t -t')( Z^{(\ell )} Y^{k-\ell } )(t') \Vert _{W^{s, p }_\mu } dt' \\&\le \Vert P(t-r) Y(r) \Vert _{W^{s,p }_\mu } + \sum _{\ell =0}^k \sum _{j = 0}^\infty e^{-\frac{\mu }{p} 2^{j \delta }} \int _r^t \Vert \phi _j P(t -t')( Z^{(\ell )}Y^{k-\ell } )(t') \Vert _{W^{s,p}} dt' . \end{aligned} \end{aligned}$$

Let $F_{k. \ell } = Z^{(\ell )}Y^{k-\ell }$. We proceed as in the proof of Lemma 2.5 and estimate

$$\begin{aligned}\Vert \phi _j P(t -t')F_{k, \ell } \Vert _{W^{s,p}}.\end{aligned}$$

With (2.35), the fractional Leibniz rule (Lemma 2.8 (i)), Young’s inequality, and (2.37), we have

3.31 $$\begin{aligned} \begin{aligned} \Vert \phi _j P(t) F_{k, \ell }\Vert _{W^{s,p}}&\lesssim e^{-t} \sum _{(m, m') \in \Lambda _{t, j}} \big \Vert \phi _j \big [ (\phi ^t_m p_t)*( \phi _{m'}F_{k, \ell }) \big ] \big \Vert _{W^{s, p}} \\&\lesssim e^{-t} \sum _{(m, m') \in \Lambda _{t, j}} \Vert \phi _j \Vert _{W^{s,\infty }} \big \Vert (\phi ^t_m p_t)*( \phi _{m'}F_{k, \ell }) \big \Vert _{W^{s, p}} \\&\lesssim e^{-t} \sum _{(m, m') \in \Lambda _{t, j}} \Vert \phi ^t_m p_t \Vert _{W^{s+\theta , 1}} \Vert \phi _{m'}F_{k, \ell } \Vert _{W^{-\theta , p}}\\&\lesssim t^{-\frac{s+\theta }{2}} e^{-t} \sum _{(m, m') \in \Lambda _{t, j}} e^{ -c_1 4^m } \Vert \phi _{m'}F_{k, \ell } \Vert _{W^{-\theta , p}} \end{aligned} \end{aligned}$$

for any $\theta > 0$, where $\phi ^t_m$ and $\Lambda _{t, j}$ are as in (2.34) and (2.36). In the following, we choose $\theta > 0$ sufficiently small such that $s + \theta < 1$.

From (2.21), Lemma 2.8 (ii) (with finite $q \gg 1$), the fractional Leibniz rule (Lemma 2.8 (i) with $q_0 \gg 1$), the interpolation (2.2), and Hölder’s inequality with (2.18), we have

3.32 $$\begin{aligned} \begin{aligned} \Vert \phi _{m'}F_{k, \ell } \Vert _{W^{-\theta , p}}&= \Vert (\widetilde{\phi }_{m'} Y)^{k-\ell } \cdot \phi _{m'} Z^{(\ell )} \Vert _{W^{-\theta , p}} \\&\lesssim \Vert (\widetilde{\phi }_{m'} Y)^{k - \ell } \Vert _{W^{\theta , p-}} \Vert \phi _{m'} Z^{(\ell )} \Vert _{W^{-\theta ,q}} \\&\lesssim \Vert \widetilde{\phi }_{m'}Y \Vert _{L^{q_0}}^{k - \ell -1} \Vert \widetilde{\phi }_{m'}Y \Vert _{W^{\theta , p}} \Vert \phi _{m'} Z^{(\ell )} \Vert _{W^{-\theta ,q}} \\&\lesssim \Vert \widetilde{\phi }_{m'}Y \Vert _{L^{q_0}}^{k-\ell -1} \Vert \widetilde{\phi }_{m'}Y \Vert _{W^{s, p}}^{\frac{\theta }{s}} \Vert \widetilde{\phi }_{m'}Y \Vert _{L^{p}}^{1- \frac{\theta }{s}} \Vert \phi _{m'} Z^{(\ell )} \Vert _{W^{-\theta , q}}\\&\lesssim \Vert \widetilde{\phi }_{m'}Y \Vert _{L^{q_0}}^{k-\ell -1} \Vert \widetilde{\phi }_{m'}Y \Vert _{W^{s, p}}^{\frac{\theta }{s}} \\&\quad \times 2^{(\frac{1}{p}- \frac{1}{q_0})(1- \frac{\theta }{s}) 2m'} \Vert \widetilde{\phi }_{m'}Y \Vert _{L^{q_0}}^{1- \frac{\theta }{s}} \Vert \phi _{m'} Z^{(\ell )} \Vert _{W^{-\theta , q}}, \end{aligned} \end{aligned}$$

provided that $0 < \theta \le s$. Then, it follows from (3.32) with (2.23) (for $\widetilde{\phi }_{m'}$ defined in (2.20)) that

3.33 $$\begin{aligned} \begin{aligned} \Vert \phi _{m'}F_{k, \ell } \Vert _{W^{-\theta , p}}&\lesssim 2^{(\frac{1}{p}- \frac{1}{q_0})(1- \frac{\theta }{s}) 2m'} e^{\frac{\mu }{q_0} a_1 (k-\ell -\frac{\theta }{s})2^{(m'+1)\delta }} \Vert Y \Vert _{L^{q_0}_\mu }^{k-\ell -\frac{\theta }{s}} \\&\quad \times e^{\frac{\mu }{p}\frac{\theta }{s} 2^{(m'+1)\delta }} \Vert Y \Vert _{W^{s, p}_\mu }^{\frac{\theta }{s}} \, e^{\frac{\mu }{q} 2^{m'\delta }} \Vert Z^{(\ell )} \Vert _{W^{-\theta , q}_\mu }. \end{aligned} \end{aligned}$$

Note that, given any small $\kappa _0>0$, there exists $C(p, q_0, s, \theta , \kappa _0) > 0$ such that

3.34 $$\begin{aligned} 2^{(\frac{1}{p}- \frac{1}{q_0})(1- \frac{\theta }{s}) 2m'} \le C(p, q_0, s, \theta , \kappa _0) e^{\kappa _0 2^{m'\delta }} \end{aligned}$$

for any $m' \in {\mathbb {Z}}_{\ge 0}$. Hence, from (3.31) and (3.33) with (3.34), we have

$$\begin{aligned}&\sum _{j = 0}^\infty e^{-\frac{\mu }{p} 2^{j \delta }} \Vert \phi _j P(t)F_{k, \ell } \Vert _{W^{s,p}} \\&\quad \lesssim t^{-\frac{s+\theta }{2}} e^{-t} \sum _{j = 0}^\infty e^{-\frac{\mu }{p} 2^{j \delta }} \sum _{(m, m') \in \Lambda _{t, j}} e^{ -c_1 4^m } e^{\widetilde{\mu }2^{m'\delta }}\\&\quad \quad \times \Vert Y \Vert _{L^{q_0}_\mu }^{k-\ell -\frac{\theta }{s}} \Vert Y \Vert _{W^{s, p}_\mu }^{\frac{\theta }{s}} \Vert Z^{(\ell )} \Vert _{W^{-\theta , q}_\mu }, \end{aligned}$$

where $\widetilde{\mu }$ is given by

$$\begin{aligned} \widetilde{\mu }= \frac{\mu }{q_0} a_1 \bigg (k-\ell -\frac{\theta }{s}\bigg )2^{\delta } + \frac{\mu }{p} \frac{\theta }{s} 2^{\delta } + \frac{\mu }{q} + \kappa _0. \end{aligned}$$

By choosing sufficiently large $q_0, q \gg 1$, $0 < \theta \ll s$, and sufficiently small $\kappa _0 > 0$, we have $p \widetilde{\mu }\ll \mu$. Then, by summing over $m'$ with (2.36) (as in the proof of Lemma 2.5), then summing over *m* (with (2.40); see also (2.41)), and finally summing over *j* with $p \widetilde{\mu }\ll \mu$, we obtain

3.35 $$\begin{aligned} \begin{aligned}&\sum _{j = 0}^\infty e^{-\frac{\mu }{p} 2^{j \delta }} \Vert \phi _j P(t)F_{k, \ell } \Vert _{W^{s,p}} \\&\quad \lesssim t^{-\frac{s+\theta }{2}} e^{-t} \sum _{j, m = 0}^\infty e^{-\frac{\mu }{p} 2^{j \delta }} e^{ -c_1 4^m } e^{c_0 \widetilde{\mu }(2^{j\delta } + 2^{m\delta } t^\frac{\delta }{2})}\\&\quad \quad \times \Vert Y \Vert _{L^{q_0}_\mu }^{k-\ell -\frac{\theta }{s}} \Vert Y \Vert _{W^{s, p}_\mu }^{\frac{\theta }{s}} \Vert Z^{(\ell )} \Vert _{W^{-\theta , q}_\mu }\\&\quad \lesssim t^{-\frac{s+\theta }{2}} e^{-c t} \sum _{j = 0}^\infty e^{-(\frac{\mu }{p} -c_0 \widetilde{\mu }) 2^{j \delta }} \Vert Y \Vert _{L^{q_0}_\mu }^{k-\ell -\frac{\theta }{s}} \Vert Y \Vert _{W^{s, p}_\mu }^{\frac{\theta }{s}} \Vert Z^{(\ell )} \Vert _{W^{-\theta , q}_\mu } \\&\quad \lesssim t^{-\frac{s+\theta }{2}} e^{-c t} \Vert Y \Vert _{L^{q_0}_\mu }^{k-\ell -\frac{\theta }{s}} \Vert Y \Vert _{W^{s, p}_\mu }^{\frac{\theta }{s}} \Vert Z^{(\ell )} \Vert _{W^{-\theta , q}_\mu } \end{aligned} \end{aligned}$$

for any $$t > 0$$.

Therefore, from (3.30), (3.35), Young’s inequality, and Lemma 2.3 (with some $0< \mu _1 < \mu$), we have

3.36 $$\begin{aligned} \begin{aligned} \Vert Y(t)\Vert _{W^{s, p}_\mu }&\lesssim \Vert P(t-r) Y(r)\Vert _{W^{s, p}_\mu }\\&\quad + \sum _{\ell =0}^k \int _r^t (t-t')^{-\frac{s+\theta }{2}} \Vert Y(t') \Vert _{L^{q_0}_\mu }^{k-\ell -\frac{\theta }{s}} \Vert Y (t') \Vert _{W^{s, p}_\mu }^{\frac{\theta }{s}} \Vert Z^{(\ell )} (t') \Vert _{W^{-\theta , q}_\mu } dt'\\&\lesssim \Vert P(t-r) Y(r)\Vert _{W^{s, p}_\mu } + \int _r^t (t-t')^{-\frac{s+\theta }{2}} \Vert Y (t') \Vert _{W^{s, p}_\mu } dt'\\&\quad + \int _r^t (t-t')^{-\frac{s+\theta }{2}} \bigg ( \Vert Y(t') \Vert _{L^{q_0}_\mu }^{q_0} +\sum _{\ell =0}^k \Vert Z^{(\ell )} (t') \Vert _{B^{-\frac{\theta }{2}, \mu _1}_{q, q}}^q + 1 \bigg ) dt'. \end{aligned} \end{aligned}$$

From Proposition 3.1 with (3.7), there exists $\lambda = \lambda (q_0, \mu ) > 1$ such that

3.37 $$\begin{aligned} \begin{aligned} \Vert Y(t') \Vert _{L^{q_0}_\mu }&\lesssim (t')^{-\frac{1}{(\lambda - 1) q_0} }\vee C_{{\bar{Z}}, q_0, \mu }(t')\\&\le r^{-\frac{1}{(\lambda - 1) q_0} } \vee C_{{\bar{Z}}, q_0, \mu }(t) \end{aligned} \end{aligned}$$

for $r \le t' \le t$. Then, from (3.36), Lemma 2.5, (3.37), and (3.29), we have

3.38 $$\begin{aligned} \begin{aligned} \Vert Y(t)\Vert _{W^{s, p}_\mu }&\lesssim (t-r)^{-\frac{s}{2}}\Vert Y(r)\Vert _{L^{p}_{\mu '}}\\&\quad +C(T) \Big ( r^{-\frac{1}{\lambda - 1} }\vee \big (C_{{\bar{Z}}, q_0, \mu }(t)\big )^{q_0} + Q_{{\bar{Z}}, \theta , q, \mu _1} (t) + 1\Big )\\&\quad + \int _r^t (t-t')^{-\frac{s+\theta }{2}} \Vert Y (t') \Vert _{W^{s, p}_\mu } dt'. \end{aligned} \end{aligned}$$

Let

3.39 $$\begin{aligned} \begin{aligned} B(t, r)&= (t-r)^{-\frac{s}{2} -}\Vert Y(r)\Vert _{L^{p}_{\mu '}}\\&\quad +C(T) \Big ( r^{-\frac{1}{\lambda - 1} }\vee \big (C_{{\bar{Z}}, q_0, \mu }(t)\big )^{q_0} + Q_{{\bar{Z}}, \theta , q, \mu _1} (t) + 1\Big ). \end{aligned} \end{aligned}$$

Then, recalling that $s + \theta < 1$, it follows from (3.38) and Cauchy-Schwarz’s inequality that

$$\begin{aligned} \Vert Y(t)\Vert _{W^{s, p}_\mu }^2&\le C B(t, r)^2 + C(T) \int _r^t \Vert Y (t') \Vert _{W^{s, p}_\mu }^2 dt'. \end{aligned}$$

Hence, by Gronwall’s inequality, we obtain

3.40 $$\begin{aligned} \Vert Y(t)\Vert _{W^{s, p}_\mu } \le C(T) B(t, r) \end{aligned}$$

for any $r \le t \le T$. Finally, by choosing $r = \frac{t}{2}$, it follows from (3.40), (3.39) and Proposition 3.1 that

$$\begin{aligned} \Vert Y(t)\Vert _{W^{s, p}_\mu }&\le C(T) \bigg \{ t^{-\frac{s}{2}} \Big ( t^{-\frac{1}{( \lambda ' - 1) p} } \vee C_{{\bar{Z}}, p, \mu '}(t)\Big )\\&+ t^{-\frac{1}{\lambda - 1} }\vee \big (C_{{\bar{Z}}, q_0, \mu }(t)\big )^{q_0} + Q_{{\bar{Z}}, \theta , q, \mu _1} (t) + 1 \bigg \} \end{aligned}$$

for some $\lambda ' = \lambda (p, \mu ') > 1$. This concludes the proof of Proposition 3.3. $\square$

### Construction of the Gibbs measure $\vec {\rho }_\infty$ on the plane

We conclude this section by presenting the proof of Theorem 1.2 (i).

Let *X* be a global-in-time solution to (3.1) on ${\mathbb {R}}^2$ constructed in [60], satisfying the decomposition $X = Y + Z$ in (3.3) with *Y* and *Z* satisfying (3.4) and (3.2), respectively. Fix $s < 0$, finite $p \ge 1$, and $\mu > 0$. Then, by Lemma 2.3, (2.32) with $s = 0$, and Proposition 3.1, we have

3.41 $$\begin{aligned} \begin{aligned} \Vert X(t) \Vert _{W^{s, p}_\mu }&\lesssim \Vert Z(t) \Vert _{W^{s, p}_\mu } + \Vert Y(t) \Vert _{L^p_{\mu '}} \\&\lesssim \Vert Z(t) \Vert _{B^{s+ \varepsilon , \mu ''}_{p, p}} + \bigg \{ t^{-\frac{1}{(\lambda -1)p}} \vee \sum _{\ell = 0}^k \Vert :\!Z^\ell (t)\!:\Vert _{B^{-\varepsilon , \mu _0}_{p_0, p_0}}^{\frac{p_0}{\lambda p}} \bigg \} \end{aligned} \end{aligned}$$

for a suitable choice of parameters $\mu '$, $\mu ''$, $\lambda$, $p_0$, $\varepsilon$, and $\mu _0$, depending on *p* and $\mu$, where the implicit constant is independent of the initial data $X_0$.

Given $L \ge 1$, let $\xi _L$ denote the (spatially) *L*-periodic space-time white noise on ${\mathbb {R}}^2\times {\mathbb {R}}_+$ obtained by first restricting the space-time white noise $\xi$ on ${\mathbb {R}}^2\times {\mathbb {R}}_+$, appearing in (3.1), onto $\big [-\frac{L}{2}, \frac{L}{2}\big ]^2 \times {\mathbb {R}}_+$ and then extending it periodically (with the spatial period *L*) onto ${\mathbb {R}}^2 \times {\mathbb {R}}_+$. See Section 5 in [60] for a further discussion. See also (4.4) and (4.5) below. We now consider the following SNLH with the *L*-periodic space-time white noise:

3.42 $$\begin{aligned} {\left\{ \begin{array}{ll} \partial _tX_L +(1 -\Delta ) X_L + :\!X_L^k\!: \, = \sqrt{2}\xi _L \\ X_L|_{t=0} = X_{0, L}, \end{array}\right. } \end{aligned}$$

where the initial data $X_{0, L}$ is assumed to be *L*-periodic. This is the parabolic $\Phi ^{k+1}_2$-model on ${\mathbb {T}}^2_L$ studied in [24] (with $L = 1$). Write $X_L$ as

3.43 $$\begin{aligned} X_L = Y_L + Z_L, \end{aligned}$$

where $$Z_L$$ satisfies

3.44 $$\begin{aligned} {\left\{ \begin{array}{ll} \partial _tZ_L + (1-\Delta ) Z_L = \sqrt{2}\xi _L \\ Z_L|_{t = 0} = 0 \end{array}\right. } \end{aligned}$$

and $$Y_L$$ satisfies

3.45 $$\begin{aligned} {\left\{ \begin{array}{ll} \partial _tY_L + (1-\Delta ) Y_L + \sum _{\ell =0}^k {k\atopwithdelims ()\ell } :\!Z_L^\ell \!: \,Y_L^{k-\ell } = 0\\ Y_L|_{t=0} = X_{0, L}. \end{array}\right. } \end{aligned}$$

Then, by applying Lemma 2.3 and Proposition 3.1 once again we have

3.46 $$\begin{aligned} \begin{aligned} \Vert X_L(t) \Vert _{W^{s, p}_\mu }&\lesssim \Vert Z_L(t) \Vert _{W^{s, p}_\mu } + \Vert Y_L(t) \Vert _{L^p_{\mu '}} \\&\lesssim \Vert Z_L(t) \Vert _{B^{s+ \varepsilon , \mu ''}_{p, p}} + \bigg \{ t^{-\frac{1}{(\lambda -1)p}} \vee \sum _{\ell = 0}^k \Vert :\! Z_L^\ell (t)\!: \Vert _{B^{-\varepsilon , \mu _0}_{p_0, p_0}}^{\frac{p_0}{\lambda p}} \bigg \} \end{aligned} \end{aligned}$$

where the implicit constant is independent of the initial data $X_{0, L}$ and of the period $L \ge 1$. Moreover, it follows from [60, Theorem 5.1]^23^ that the *p*th moment of the right-hand side of (3.46) (and of (3.41)) is bounded, uniformly in $L \ge 1$.

Let $\rho _L$ denote the *L*-periodic $\Phi ^{k+1}_2$-measure on ${\mathbb {T}}^2_L$, appearing in (1.21). By taking ${{\,\textrm{Law}\,}}(X_{0, L}) = \rho _L$ in (3.42) (and we assume that $X_{0, L}$ is independent of the noise $\xi _L$), it is known [24] that

3.47 $$\begin{aligned} {{\,\textrm{Law}\,}}(X_L(t)) = \rho _L \end{aligned}$$

for any $t \ge 0$. Moreover, from (3.46) and the observation mentioned right after (3.46), we have

3.48 $$\begin{aligned} \sup _{L\ge 1} {\mathbb {E}}\Big [ \Vert X_L(1) \Vert _{W^{s, p}_\mu }^p \Big ] \lesssim 1. \end{aligned}$$

Now, given $s < 0$ and $\mu > 0$, let $s< s' < 0$ and $0 < \mu ' \ll \mu$. Then, given $M > 0$, set

$$\begin{aligned} K_M = \big \{ f\in W^{s, p}_{\mu } ({\mathbb {R}}^2) : \Vert f\Vert _{W^{s', p}_{\mu '}} \le M \big \}. \end{aligned}$$

Then, it follows from Chebyshev’s inequality and (3.48) that, given $\varepsilon > 0$, there exists $M > 0$ such that

$$\begin{aligned} \rho _L\big ( K_M^c \big )&= {\mathbb {P}}\Big ( \Vert X_L(1; \omega )\Vert _{W^{s', p}_{\mu '}} > M \Big ) \lesssim M^{-p} < \varepsilon , \end{aligned}$$

uniformly in $L\ge 1$. On the other hand, it follows from Lemma 2.2 that $K_M$ is compact in $W^{s, p}_{\mu }({\mathbb {R}}^2)$. Therefore, from the Prokhorov theorem (Lemma 2.18), we conclude that $\{\rho _L\}_{L \in {\mathbb {N}}}$ is tight and hence admits a subsequence $\{\rho _{L_j}\}_{j \in {\mathbb {N}}}$ which converges weakly to a limiting $\Phi ^{k+1}_2$-measure $\rho _\infty$ on ${\mathbb {R}}^2$ as $j \rightarrow \infty$.

Next, we prove weak convergence of the *L*-periodic white noise measure $\mu _{0, L}$ defined in (1.11) (with $s = 0$) to the limiting white noise measure on ${\mathbb {R}}^2$. Formally, a (spatial) white noise $\zeta$ on ${\mathbb {R}}^2$ is a centered Gaussian distribution on ${\mathbb {R}}^2$ with covariance

3.49 $$\begin{aligned} {\mathbb {E}}\big [\zeta (x_1) \zeta (x_2)\big ] = \delta (x_1 - x_2). \end{aligned}$$

The expression (3.49) is merely formal but we can make it rigorous by testing it against a test function.

#### Definition 3.4

A (spatial) white noise $\zeta$ on ${\mathbb {R}}^2$ is a family of centered Gaussian random variables $\{ \zeta (\varphi ): \varphi \in L^2({\mathbb {R}}^2)\}$ such that

$$\begin{aligned} {\mathbb {E}}\big [ \zeta (\varphi )^2 \big ] = \Vert \varphi \Vert _{L^2({\mathbb {R}}^2)}^2 \quad \text {and} \quad {\mathbb {E}}\big [ \zeta (\varphi _1) \zeta ( \varphi _2)\big ] = \langle \varphi _1, \varphi _2 \rangle _{L^2({\mathbb {R}}^2)}. \end{aligned}$$

Given $k \in {\mathbb {N}}$, let $\eta _k$ be as in (2.9). Then, a direct computation with Lemma 2.12 shows

3.50 $$\begin{aligned} {\mathbb {E}}\big [ |\zeta (\eta _k)|^p\big ] \lesssim _p \Big ({\mathbb {E}}\big [ \zeta (\eta _k)^2\big ]\Big )^\frac{p}{2} = \Vert \eta _k\Vert _{L^2}^p \sim 2^{kp} \end{aligned}$$

for any finite $p \ge 1$. Hence, we conclude from (the time-independent version of) Lemma 9 in [60]^24^ that the white noise $\zeta$ on ${\mathbb {R}}^2$ belongs almost surely to $B^{s, \mu }_{p, q}({\mathbb {R}}^2)$ for any $s < -1$, $\mu > 0$, $1\le p < \infty$, and $1\le q\le \infty$, with the following bound:

3.51 $$\begin{aligned} {\mathbb {E}}\big [\Vert \zeta \Vert _{B^{s, \mu }_{p, q}}^r\big ] \le C(r, s, \mu , p, q) < \infty \end{aligned}$$

for any finite $r \ge 1$. Then, by Lemma 2.3, we see that the white noise $\zeta$ on ${\mathbb {R}}^2$ also belongs almost surely to the weighted Sobolev space $W^{s, p}_\mu ({\mathbb {R}}^2)$ for any $s < -1$, $1\le p < \infty$, and $\mu > 0$, with a bound:

3.52 $$\begin{aligned} {\mathbb {E}}\big [\Vert \zeta \Vert _{W^{s, p}_\mu }^r\big ] \le C(r, s, \mu , p) < \infty \end{aligned}$$

for any finite $r \ge 1$.

#### Lemma 3.5

Let $\zeta$ be a white noise on ${\mathbb {R}}^2$ as in Definition 3.4 and set $\mu _{0, \infty } = {{\,\textrm{Law}\,}}(\zeta )$. Then, given any $s < -1$, finite $p \ge 1$, and $\mu > 0$, by viewing the *L*-periodic white noise measure $\mu _{0, L}$ defined in (1.11) with $s =0$ as a measure on $W^{s, p}_\mu ({\mathbb {R}}^2)$, the sequence $\{\mu _{0, L}\}_{L \in {\mathbb {N}}}$ converges weakly to $\mu _{0, \infty }$ as $L \rightarrow \infty$.

By putting the weak convergence of a subsequence $\{\rho _{L_j}\}_{j \in {\mathbb {N}}}$ with Lemma 3.5, we conclude that as probability measures on $\vec {W}^{s, p}_\mu ({\mathbb {R}}^2) = W^{s, p}_\mu ({\mathbb {R}}^2)\times W^{s-1, p}_\mu ({\mathbb {R}}^2)$ with $s < 0$, finite $p \ge 1$, and $\mu > 0$, the $L_j$-periodic Gibbs measure $\vec {\rho }_{L_j} = \rho _{L_j} \otimes \mu _{0, L_j}$ in (1.20) converges weakly to a limiting Gibbs measure $\vec {\rho }_\infty = \rho _\infty \otimes \mu _{0, \infty }$ on ${\mathbb {R}}^2$ as $j \rightarrow \infty$. This proves Theorem 1.2 (i).

#### Remark 3.6

In view of the embedding between weighted Sobolev spaces and weighted Besov spaces stated in Lemma 2.3, the weak convergence of the $L_j$-periodic Gibbs measure $\vec {\rho }_{L_j}$ to the limiting Gibbs measure $\vec {\rho }_\infty$ also holds as probability measures on the weighted Besov space $B^{s, \mu }_{p, q}({\mathbb {R}}^2) \times B^{s-1, \mu }_{p, q}({\mathbb {R}}^2)$ for any $s <0$, finite $p \ge 1$, $1 \le q \le \infty$, and $\mu > 0$.

We conclude this section by presenting the proof of Lemma 3.5.

#### Proof of Lemma 3.5

As we have seen in Sect. 1, an *L*-periodic white noise on the dilated torus ${\mathbb {T}}_L^2$ is given by the second Gaussian Fourier series for $v_L$ in (1.14) such that ${{\,\textrm{Law}\,}}(v_L) = \mu _{0, L}$. While it is possible to work with $v_L$ in (1.14), by viewing it as an *L*-periodic distribution on ${\mathbb {R}}^2$, and show that the *L*-periodic white noise measure $\mu _{0, L} = {{\,\textrm{Law}\,}}(v_L)$ converges weakly to the white noise measure $\mu _{0, \infty } = {{\,\textrm{Law}\,}}(\zeta )$ on ${\mathbb {R}}^2$, it is more convenient to work with the *L*-periodized version $\zeta _L$ of the white noise $\zeta$. Define $\zeta _L$ on ${\mathbb {T}}^2_L$ by setting

3.53 $$\begin{aligned} \zeta _L= \sum _{\lambda \in {\mathbb {Z}}^2_L} \widehat{\zeta }_L (\lambda ) e_\lambda ^L, \end{aligned}$$

where $e_\lambda ^L$ is as in (1.10) and $\widehat{\zeta }_L(\lambda )$ is given by

3.54 $$\begin{aligned} \begin{aligned} \widehat{\zeta }_L(\lambda )&= \int _{[-\frac{L}{2}, \frac{L}{2})^2} \zeta (x) \overline{e_\lambda ^L(x)} dx = \zeta \Big ( {\textbf{1}}_{[-\frac{L}{2}, \frac{L}{2})^2} \overline{e_\lambda ^L}\Big )\\ : \!&= \zeta \Big ( {\textbf{1}}_{[-\frac{L}{2}, \frac{L}{2})^2} \mathop {\textrm{Re}}\limits (e_\lambda ^L)\Big ) - i \zeta \Big ( {\textbf{1}}_{[-\frac{L}{2}, \frac{L}{2})^2} \mathop {\textrm{Im}}\limits (e_\lambda ^L)\Big ). \end{aligned} \end{aligned}$$

Then, by viewing $\zeta _L$ as an *L*-periodic distribution on ${\mathbb {R}}^2$, we see that ${{\,\textrm{Law}\,}}(\zeta _L) = \mu _{0, L}$. Indeed, it follows from Definition 3.4 and the orthonormality of $\{e_\lambda ^L\}_{\lambda \in {\mathbb {Z}}^2_L}$ on ${\mathbb {T}}^2_L = \big [-\frac{L}{2}, \frac{L}{2}\big )^2$ that $\{ \widehat{\zeta }_L(\lambda )\}_{\lambda \in {\mathbb {Z}}^2_L}$ forms a family of independent standard complex-valued Gaussian random variables conditioned that $\widehat{\zeta }_L(-\lambda ) = \overline{\widehat{\zeta }_L(\lambda )}$, $\lambda \in {\mathbb {Z}}^2_L$. Hence, from (1.14) and (3.53), we conclude that ${{\,\textrm{Law}\,}}(\zeta _L) = {{\,\textrm{Law}\,}}(v_L) = \mu _{0, L}$. Before proceeding further, we point out that by repeating the computation (3.50) for $\zeta _L$, we see that the bounds (3.51) and (3.52) also hold for $\zeta _L$, uniformly in $L \ge 1$.

By definition, $\zeta _L$ agrees with $\zeta$ on ${\mathbb {T}}^2_L = \big [-\frac{L}{2}, \frac{L}{2}\big )^2$. Now, fix $s < -1$, $1 \le p < \infty$, and $\mu > 0$. Then, given finite $r \ge 1$, it follows from (3.52) for $\zeta$ and $\zeta _L$ that

3.55 $$\begin{aligned} \begin{aligned} {\mathbb {E}}\Big [ \Vert \zeta - \zeta _L \Vert _{W^{s, p}_\mu ({\mathbb {R}}^2)}^p\big ]&= {\mathbb {E}}\Big [\Vert \zeta - \zeta _L \Vert _{W^{s, p}_\mu (({\mathbb {T}}^2_L)^c)}^p\big ]\\&\le C(r, s, \mu ', p) \cdot w_{\mu - \mu '}(c_0L) \end{aligned} \end{aligned}$$

for any $0< \mu ' < \mu$, where $c_0>0$ is an absolute constant independent of $L \ge 1$ (and the other parameters). Then, summing over $L \in {\mathbb {N}}$, we obtain

$$\begin{aligned} {\mathbb {E}}\bigg ( \sum _{L= 1}^\infty \Vert \zeta - \zeta _L \Vert ^p_{W^{s,p}_\mu ({\mathbb {R}}^2)} \bigg )&= \sum _{L = 1}^\infty {\mathbb {E}}\Big [ \Vert \zeta - \zeta _L \Vert ^p_{W^{s,p}_\mu ({\mathbb {R}}^2)} \Big ] \\&\lesssim \sum _{L= 1}^\infty w_{\mu - \mu '} (c_0 L) < \infty , \end{aligned}$$

since $\mu > \mu '$. This implies immediately that $\sum _{L = 1}^\infty \Vert \zeta - \zeta _L \Vert ^p_{W^{s,p}_\mu ({\mathbb {R}}^2)}$ is finite almost surely, which in particular implies that $\zeta _L$ converges almost surely to $\zeta$ in $W^{s,p}_\mu ({\mathbb {R}}^2)$ as $L\rightarrow \infty$ (with $L \in {\mathbb {N}}$). Therefore, we conclude that $\mu _{0, L} = {{\,\textrm{Law}\,}}(\zeta _L)$ converges weakly to $\mu _{0, \infty } = {{\,\textrm{Law}\,}}(\zeta )$ as $L \rightarrow \infty$ (with $L \in {\mathbb {N}}$). This conclude the proof of Lemma 3.5. $\square$

## Global Well-Posedness and Invariance of the Gibbs Measure

In this section, we present the proof of Theorem 1.2 (ii). Our main goal is to construct global-in-time dynamics for the hyperbolic $\Phi ^{k+1}_2$-model on ${\mathbb {R}}^2$ with the Gibbsian initial data:

4.1 $$\begin{aligned} {\left\{ \begin{array}{ll} \partial _t^2 u + \partial _tu + (1- \Delta )u \, + :\!u^k\!: \, = \sqrt{2}\xi \\ (u, \partial _tu)|_{t = 0} = (u_0, u_1)\quad \text {with } {{\,\textrm{Law}\,}}(u_0, u_1) = \vec {\rho }_\infty , \end{array}\right. } \end{aligned}$$

where $\vec {\rho }_\infty = \rho _\infty \otimes \mu _{0, \infty }$ is the Gibbs measure on ${\mathbb {R}}^2$, constructed as a limit of the $L_j$-periodic Gibbs measure $\vec {\rho }_{L_j} = \rho _{L_j} \otimes \mu _{0, L_j}$ in the previous section. For simplicity of notation, we set

$$\begin{aligned} {\mathcal {A}}= \{ L_j: j \in {\mathbb {N}}\} \subset {\mathbb {N}}\end{aligned}$$

and only consider the values of $L \in {\mathcal {A}}$ in the following, unless otherwise specified.

Consider the *L*-periodic hyperbolic $\Phi ^{k+1}_2$-model on ${\mathbb {R}}^2$:

4.2 $$\begin{aligned} {\left\{ \begin{array}{ll} \partial _t^2 u_L + \partial _tu_L + (1- \Delta )u_L \, + :\!u_L^k\!: \, = \sqrt{2}\xi _L\\ (u_L, \partial _tu_L)|_{t = 0} = (u_{0, L}, u_{1, L})\quad \text {with } {{\,\textrm{Law}\,}}(u_{0, L}, u_{1, L}) = \vec {\rho }_L, \end{array}\right. } \end{aligned}$$

where $\xi _L$ is an *L*-periodic space-time white noise (see (4.4) and (4.5) below) and $\vec {\rho }_L = \rho _L \otimes \mu _{0, L}$ is the *L*-periodic Gibbs measure in (1.20); see also Remark 1.1. As mentioned in Sect. 1, the Cauchy problem (4.2) is globally well-posed and the Gibbs measure $\vec {\rho }_L$ is invariant under the resulting dynamics. In the following, we construct a solution to (4.1) as a limit of the solutions $u_L$ to (4.2), $L \in {\mathcal {A}}$.

Let us first introduce some notations. Let $\Psi$ be the solution to the following linear stochastic damped wave equation:

4.3 $$\begin{aligned} {\left\{ \begin{array}{ll} \partial _t^2 \Psi + \partial _t\Psi + (1- \Delta )\Psi = \sqrt{2}\xi \\ (\Psi , \partial _t\Psi )|_{t=0} = ( 0, 0). \end{array}\right. } \end{aligned}$$

With ${\mathcal {D}}(t)$ as in (1.23), we formally have

$$\begin{aligned} \Psi (t) =&\sqrt{2} \int _0^t {\mathcal {D}}(t-t') \xi (dt'). \end{aligned}$$

Let $L \ge 1$. Given a spatial white noise $\zeta$ on ${\mathbb {R}}^2$, we let $\zeta _L$ denote the *L*-periodized version of $\zeta$ defined in (3.53) and (3.54). Similarly, let $\xi _L$ be the *L*-periodized version (in space) of the space-time white noise $\xi$. Namely, we define $\xi _L$ on ${\mathbb {T}}^2_L\times {\mathbb {R}}_+ \cong \big [-\frac{L}{2}, \frac{L}{2}\big )^2 \times {\mathbb {R}}_+$ by setting

4.4 $$\begin{aligned} \xi _L= \sum _{\lambda \in {\mathbb {Z}}^2_L} \widehat{\xi }_L (\lambda ) e_\lambda ^L, \end{aligned}$$

where $e_\lambda ^L$ is as in (1.10) and $\widehat{\xi }_L(\lambda )$ is given by

4.5 $$\begin{aligned} \widehat{\xi }_L(\lambda ) = \int _{[-\frac{L}{2}, \frac{L}{2})^2} \xi (x) \overline{e_\lambda ^L(x)} dx = \xi \Big ( {\textbf{1}}_{[-\frac{L}{2}, \frac{L}{2})^2} \, \overline{e_\lambda ^L}\Big ) \end{aligned}$$

with the equality understood in the sense of temporal distributions. Alternatively, $\widehat{\xi }_L(\lambda )$ is given as the (distributional) time derivative of

$$\begin{aligned} \widehat{B}_L(\lambda , t) = \xi \Big ( {\textbf{1}}_{[0, t]} \cdot {\textbf{1}}_{[-\frac{L}{2}, \frac{L}{2})^2}(x) \, \overline{e_\lambda ^L(x)}\Big ), \end{aligned}$$

where the right-hand side is the action of $\xi$ on a function in $L^2({\mathbb {R}}^2 \times {\mathbb {R}}_+)$. It is then easy to check that $\{\widehat{\xi }_L(\lambda ) \}_{\lambda \in {\mathbb {Z}}^2_L}$ forms a family of independent temporal white noises conditioned that $\widehat{\xi }_L(- \lambda ) = \overline{\widehat{\xi }_L(\lambda )}$, $\lambda \in {\mathbb {Z}}^2_L$. Compare this with (1.24). By the *L*-periodic extension in space, we then view $\xi _L$ as a space-time distribution on ${\mathbb {R}}^2\times {\mathbb {R}}_+$.

In the following, we construct a solution *u* to (4.1) on the cone ${\textbf{C}}_R$ defined in (1.33) for each $R >0$ as a limit of the solution $u_L$ to (4.2). Fix $R >0$. Then, thanks to the finite speed of propagation, we have

4.6 $$\begin{aligned} \zeta _L = \zeta \quad \text {and} \quad \xi _L = \xi \quad \text {on the cone }{\textbf{C}}_R, \end{aligned}$$

provided that $L \ge 2R$. In particular, on the cone ${\textbf{C}}_R$, the equation (4.2) agrees (in law) with

4.7 $$\begin{aligned} {\left\{ \begin{array}{ll} \partial _t^2 u_L + \partial _tu_L + (1- \Delta )u_L \, + :\!u_L^k\!: \, = \sqrt{2}\xi \\ (u_L, \partial _tu_L)|_{t = 0} = (u_{0, L}, u_{1})\quad \text {with } {{\,\textrm{Law}\,}}(u_{0, L}) = \rho _L, \end{array}\right. } \end{aligned}$$

where $\xi$ and $u_1$ with ${{\,\textrm{Law}\,}}(u_1) = \mu _{0, \infty }$ are as in (4.1). Namely, the difference between (4.1) and (4.7) appears only in the first component of the initial data. This motivates the following decomposition of a solution.

We write a solution *u* to (4.1) as

4.8 $$\begin{aligned} \begin{aligned} u(t)&= \Phi (t) + v (t)\\ : \!&= {\mathcal {S}}(t)u_0 + {\mathcal {D}}(t) u_1 + \Psi (t) + v (t), \end{aligned} \end{aligned}$$

where ${\mathcal {S}}(t)$, ${\mathcal {D}}(t)$, and $\Psi (t)$ are as in (2.5), (1.23), and (4.3), and *v* satisfies the following equation:

4.9 $$\begin{aligned} \begin{aligned} v(t)&= - \int _0^t {\mathcal {D}}(t-t') : \!u^k(t')\! : dt' \\&= - \sum _{\ell = 0}^k {k\atopwithdelims ()\ell } \int _0^t {\mathcal {D}}(t - t')\big ( \!:\! \Phi ^\ell (t')\!: v^{k- \ell }(t')\big ) dt'. \end{aligned} \end{aligned}$$

We also set

4.10 $$\begin{aligned} \Phi _0(t) = \Phi _0(u_0)(t) = {\mathcal {S}}(t) u_0 \quad \text {and} \quad \Phi _1(t) = \Phi _1(u_1, \xi ) (t) = {\mathcal {D}}(t)u_1 + \Psi (t) \end{aligned}$$

such that

$$\begin{aligned}\Phi (u_0, u_1, \xi ) = \Phi _0(u_0) + \Phi _1(u_1, \xi ).\end{aligned}$$

Then, by the independence of $u_0$ and $(u_1, \xi )$ in (4.1),^25^ we have

$$\begin{aligned} :\! \Phi ^\ell (t)\!: \,\, = \,\, :\! \Phi ^\ell (u_0, u_1, \xi )(t)\!: \,\, = \sum _{m = 0}^\ell {\ell \atopwithdelims ()m} :\! \Phi _0^m(u_0) (t)\!: \, :\! \Phi _1^{\ell -m}(u_1, \xi ) (t)\!:\, , \end{aligned}$$

and thus we can rewrite (4.9) as

4.11 $$\begin{aligned} \begin{aligned} v(t)&= - \sum _{\ell = 0}^k \sum _{m = 0}^\ell {k\atopwithdelims ()\ell } {\ell \atopwithdelims ()m}\\&\times \int _0^t {\mathcal {D}}(t - t')\big ( \! :\! \Phi _0^m(u_0) (t')\!: \, :\! \Phi _1^{\ell -m}(u_1, \xi ) (t')\!: v^{k- \ell }(t')\big ) dt'. \end{aligned} \end{aligned}$$

In view of (4.11), we define enhanced data sets $\Xi _0$ and $\Xi _1$ by setting

4.12 $$\begin{aligned} \begin{aligned} \Xi _0(\phi )&= \big ( \Phi _0(\phi ), : \!\Phi _0^2(\phi )\! :\,, \dots , \, : \!\Phi _0^k(\phi )\!: \big )\\&= \big ( {\mathcal {S}}(t)\phi , : \!({\mathcal {S}}(t) \phi )^2\! :\,, \dots , \, : \!({\mathcal {S}}(t) \phi )^k\!: \big ) \end{aligned} \end{aligned}$$

and

4.13 $$\begin{aligned} \Xi _1(u_1, \xi ) = \big ( \Phi _1, : \!\Phi _1^2\!:\,, \dots , \, : \!\Phi _1^k\!: \big ), \end{aligned}$$

where $\Phi _1 = \Phi _1(u_1, \xi )$ is as in (4.10).

We now introduce distances / sizes by which we measure these enhanced data sets.

### Definition 4.1

Let $k \in 2{\mathbb {N}}+ 1$. Let $0 < \varepsilon \ll 1$, finite $p \gg 1$, and $\mu > 0$ (to be chosen later).

(i) We first introduce a space ${\mathbb {W}}$ for the first enhanced data set $\Xi _0(\phi )$ in (4.12) by setting

$$\begin{aligned} {\mathbb {W}}= \big (C({\mathbb {R}}_+; W^{-\varepsilon , p}_\mu ({\mathbb {R}}^2))\big )^{\otimes k}. \end{aligned}$$

We endow ${\mathbb {W}}$ with the following bounded metric:

4.14 $$\begin{aligned} \begin{aligned} d_{\mathbb {W}}(f, g)&= d_{\mathbb {W}}\big ((f_1, \dots , f_k), (g_1, \dots , g_k)\big ) \\&= \sum _{\ell =1}^\infty \frac{1}{2^\ell } \frac{ \sum _{j =1} ^k\Vert f_j-g_j\Vert _{C ([ 0, 2^\ell ]; W^{-\varepsilon , p}_\mu ({\mathbb {R}}^2))}}{ 1 + \sum _{j =1} ^k \Vert f_j-g_j\Vert _{C ([ 0, 2^\ell ]; W^{-\varepsilon , p}_\mu ({\mathbb {R}}^2))}}, \end{aligned} \end{aligned}$$

which induces the compact-open topology (in time) on ${\mathbb {W}}$. Then, the metric space $( {\mathbb {W}}, d_{\mathbb {W}})$ is a bounded Polish space.^26^

Given $R > 0$, we also set

4.15 $$\begin{aligned} {\mathbb {W}}(R) = \big (L^\infty ([0; R]; W^{-\varepsilon , p}(B_R))\big )^{\otimes k} \end{aligned}$$

with the norm given by

$$\begin{aligned} \Vert f \Vert _{{\mathbb {W}}(R)} = \Vert (f_1, \dots , f_k) \Vert _{{\mathbb {W}}(R)} = \sum _{j = 1}^k \Vert f_j\Vert _{L^\infty ([0; R]; W^{-\varepsilon , p}(B_R))}. \end{aligned}$$

(ii) Let $\Xi _0 = (\Xi _{01}, \dots , \Xi _{0k}) \in {\mathbb {W}}(R)$. Given $R > 0$, let $\Vert \Xi _1 \Vert _{\Xi _0, R}$ denote the smallest constant $K_1 \ge 1$ such that the following inequality holds for $1\le j,\ell \le k$ with $j + \ell \le k$:

4.16 $$\begin{aligned} \begin{aligned} \int _0^T \Vert :\!\Phi _1^j(t)\!: \Xi _{0\ell }(t) \Vert _{W^{-2(k+1)\varepsilon , p}(B_{R})}^p dt \le K_1\int _0^T \Vert \Xi _{0\ell }(t) \Vert _{W^{-\varepsilon , p}(B_{R})}^p dt \end{aligned} \end{aligned}$$

for any $$0 < T \le R$$.

(iii) Let $\widetilde{\Xi }_0 = (\widetilde{\Xi }_{01}, \dots , \widetilde{\Xi }_{0k}) \in {\mathbb {W}}(R)$. Given $R > 0$, let $K_2, K_3 \ge 1$ be the smallest constants such that the following inequalities hold for $1\le j,\ell \le k$ with $j + \ell \le k$:

4.17 $$\begin{aligned} \begin{aligned} \int _0^T \Vert :\!\Phi _1^j(t)\! : \widetilde{\Xi }_{0\ell }(t) \Vert _{W^{-2(k+1)\varepsilon , p}(B_{R})}^p dt \le K_2\int _0^T \Vert \widetilde{\Xi }_{0\ell }(t) \Vert _{W^{-\varepsilon , p}(B_{R})}^p dt \end{aligned} \end{aligned}$$

and

4.18 $$\begin{aligned} \begin{aligned}&\int _0^T \Vert :\!\Phi _1^j(t)\! : (\, \Xi _{0\ell }(t) - \widetilde{\Xi }_{0\ell }(t) ) \Vert _{W^{-2(k+1)\varepsilon , p}(B_{R})}^p dt \\&\qquad \qquad \qquad \le K_3\int _0^T \Vert \Xi _{0\ell }(t) - \widetilde{\Xi }_{0\ell }(t) \Vert _{W^{-\varepsilon , p}(B_{R})}^p dt \end{aligned} \end{aligned}$$

for any $0 < T \le R$. Then, we set $\Vert \Xi _1 \Vert _{\Xi _0, \widetilde{\Xi }_0, R} = \max (K_1, K_2, K_3)$, where $K_1$ is as in Part (ii).

In the remaining part of this paper, we fix small $\varepsilon > 0$, finite $p = p(\varepsilon ) \gg 1$,^27^ and $\mu > 0$ (to be chosen later), and often drop dependence on these parameters. For simplicity of notation, we set

4.19 $$\begin{aligned} \varepsilon _k = 2(k+1) \varepsilon \end{aligned}$$

in the remaining part of this section. This $\varepsilon _k$ comes from the condition appearing in the proof of Proposition 4.9 (i).

### Local well-posedness and stability of SdNLW

In this subsection, we study local well-posedness and stability of SdNLW (4.1), where we view $u_0$, $u_1$, and $\xi$ in (4.1) as given deterministic spatial / space-time distributions^28^ such that the enhanced data sets $\Xi _0(u_0)$ and $\Xi _1(u_1, \xi )$ in (4.12) and (4.13) make sense. In order to emphasize the dependence on $u_0$, $u_1$, and $\xi$, we may write $u = u(u_0, u_1, \xi )$. By writing *u* as in (4.8), we study the equation (4.11) for the remainder term

4.20 $$\begin{aligned} \begin{aligned} v&=u - {\mathcal {S}}(t)u_0 - {\mathcal {D}}(t) u_1 - \Psi \\&= u - \Phi _0(u_0) - \Phi _1(u_1, \xi ). \end{aligned} \end{aligned}$$

Given $\Xi _0 = (\Xi _{01}, \dots , \Xi _{0k})$, we consider (4.11), starting from $t = t_0$ with $(v, \partial _tv)|_{t = t_0} = (v_0, v_1)$, where we replace $:\! \Phi _0^m(u_0) \!:$ by $\Xi _{0m}$:

4.21 $$\begin{aligned} \begin{aligned} v(t)&= {\mathcal {S}}(t-t_0) v_0 + {\mathcal {D}}(t- t_0) v_1\\&\quad - \sum _{\ell = 0}^k \sum _{m = 0}^\ell {k\atopwithdelims ()\ell } {\ell \atopwithdelims ()m}\\&\qquad \quad \times \int _{t_0}^t {\mathcal {D}}(t - t')\big ( \Xi _{0m} (t') :\! \Phi _1^\ell (u_1, \xi ) (t')\!: v^{k- \ell }(t')\big ) dt'. \end{aligned} \end{aligned}$$

Given $s\in {\mathbb {R}}$ and $\tau > 0$, we set

$$\begin{aligned} X_{t_0, R}^s(\tau )&= L^\infty ([t_0, \max (t_0+ \tau , R)]; H^{s}(B_{R - t}) ),\\ \vec {X}_{t_0, R}^s(\tau )&= X_{t_0, R}^s(\tau ) \times X_{t_0, R}^{s-1}(\tau ). \end{aligned}$$

#### Proposition 4.2

(local well-posedness). Let $R > 0$, $0 \le t_0 < R$, and $(v_0, v_1) \in \vec {H}^{1-\varepsilon _k}(B_{R- t_0})$, where $\varepsilon _k = 2(k+1)\varepsilon$ is as in (4.19). Suppose that there exist $M_0, M_1 \ge 1$ such that

4.22 $$\begin{aligned} \begin{aligned} \Vert \Xi _0 \Vert _{{\mathbb {W}}(R) } \le M_0<\infty ,\\ \Vert \Xi _1(u_1, \xi ) \Vert _{\Xi _0, R} \le M_1 < \infty , \end{aligned} \end{aligned}$$

where ${\mathbb {W}}(R)$ is as in (4.15) and $\Vert \Xi _1(u_1, \xi ) \Vert _{\Xi _0, R}$ is as in Definition 4.1 (ii). Then, there exist small $\tau = \tau \big (\Vert (v_0, v_1)\Vert _{\vec {H}^{1-\varepsilon _k}(B_{R- t_0})}, M_0, M_1\big )>0$ and a unique solution *v* to (4.21) on ${\textbf{C}}_R\cap \big \{ t_0\le t \le \min (t_0 + \tau , R) \big \}$ such that

4.23 $$\begin{aligned} \Vert (v, \partial _tv) \Vert _{ \vec {X}_{t_0, R}^{1-\varepsilon _k}(\tau )} \le C_0 \Vert (v_0, v_1)\Vert _{\vec {H}^{1-\varepsilon _k}(B_{R- t_0})} \end{aligned}$$

for some absolute constant $C_0 > 0$.

#### Proof

The proof of Proposition 4.2 follows from a slight modification of Proposition 4.1 in [45] together with Definition 4.1 (ii). Denote the right-hand side of (4.21) by $\Gamma (v) = \Gamma _{\Xi _0, \Xi _1(u_1, \xi )}(v)$, and set $\vec {\Gamma }(v) = ( \Gamma (v), \partial _t\Gamma (v))$. Then, by the finite speed of propagation, we have

4.24 $$\begin{aligned} \begin{aligned} \Gamma (v)(t)&={\mathcal {S}}(t-t_0) v_0 + {\mathcal {D}}(t- t_0) v_1 - \sum _{\ell = 0}^k \sum _{m = 0}^\ell {k\atopwithdelims ()\ell } {\ell \atopwithdelims ()m}\\&\times \int _{t_0}^t {\mathcal {D}}(t - t')\big ({\textbf{1}}_{{\textbf{C}}_R} \cdot \Xi _{0m} (t') :\! \Phi _1^{\ell - m}(u_1, \xi ) (t')\!: v^{k- \ell }(t')\big ) dt' \end{aligned} \end{aligned}$$

on the cone ${\textbf{C}}_R$.

Let $(\textbf{v}_0, \textbf{v}_1)$ be an extension of $(v_0, v_1)$ onto ${\mathbb {R}}^2$. Given $\vec {v} = (v, \partial _tv)$ on $\big \{(x, t) \in {\textbf{C}}_R: t \in [t_0, \max (t_0+ \tau , R)]\big \}$, let $\vec {\textbf{v}} = (\textbf{v}, \textbf{v}')$ be an extension onto ${\mathbb {R}}^2\times {\mathbb {R}}_+$. Similarly, let $\Phi ^{m, \ell -m}_{0, 1}$ be an extension of $\Xi _{0\,m} :\! \Phi _1^{\ell - m} \!:$ from $B_R \times [0, R]$ to ${\mathbb {R}}^2\times [0, R]$. Then, proceeding as in the proof of Proposition 4.1 in [45], it follows from (4.24), Lemma 2.8, and Sobolev’s inequality that

$$\begin{aligned} \Vert \vec {\Gamma }(v)\Vert _{\vec {X}_{t_0, R}^{1-\varepsilon _k}(\tau )}&\lesssim \Vert (\textbf{v}_0, \textbf{v}_1)\Vert _{\vec {H}^{1-\varepsilon _k}({\mathbb {R}}^2)} \\&\quad + \tau ^\theta \sum _{\ell = 0}^k \sum _{m = 0}^\ell c_{k, \ell , m} \Vert \Phi ^{m, \ell -m}_{0, 1}\Vert _{L^p_{[t_0, t_0+\tau ]} W^{-\varepsilon _k, p}_x({\mathbb {R}}^2)} \Vert \textbf{v}\Vert _{L^\infty _{[t_0, t_0+\tau ]} H^{1-\varepsilon _k}_x({\mathbb {R}}^2) }^{k-\ell } \end{aligned}$$

for some $\theta > 0$ and large $p \gg 1$, where $\varepsilon _k = 2(k+1)\varepsilon$ is as in (4.19) with sufficiently small $\varepsilon > 0$. Then, by taking infima over all the extensions $(\textbf{v}_0, \textbf{v}_1)$, $\vec {\textbf{v}}$, and $\Phi ^{m, \ell -m}_{0, 1}$ and then using Definition 4.1 (ii) with (4.22), we obtain

4.25 $$\begin{aligned} \begin{aligned} \Vert \vec {\Gamma }(v)\Vert _{\vec {X}_{t_0, R}^{1-\varepsilon _k}(\tau )}&\lesssim \Vert (v_0,v_1)\Vert _{\vec {H}^{1-\varepsilon _k}(B_{R- t_0})}\\&\quad + \tau ^\theta M_1 \sum _{\ell = 0}^k \sum _{m = 0}^\ell c_{k, \ell , m} \Vert \Xi _{0m} \Vert _{L^p_\tau W^{-\varepsilon , p}_x(B_{R })} \Vert \vec {v}\Vert _{\vec {X}_{t_0, R}^{1-\varepsilon _k}(\tau ) }^{k-\ell }\\&\lesssim \Vert (v_0,v_1)\Vert _{\vec {H}^{1-\varepsilon _k}(B_{R- t_0})} + \tau ^\theta M_0 M_1 \Big (1 + \Vert \vec {v}\Vert _{X_{t_0, R}^{1-\varepsilon _k}(\tau )}\Big )^{k}. \end{aligned} \end{aligned}$$

A similar argument yields the following difference estimate:

4.26 $$\begin{aligned} \begin{aligned}&\Vert \vec {\Gamma }(v_1) - \vec {\Gamma }(v_2)\Vert _{\vec {X}^{1-\varepsilon _k}_{t_0, R}(\tau )}\\&\quad \lesssim \tau ^\theta M_0 M_1 \Big (1 + \Vert \vec {v}_1\Vert _{X_{t_0, R}^{1-\varepsilon _k}(\tau ) }^{k-1}+ \Vert \vec {v}_2\Vert _{\vec {X}_{t_0, R}^{1-\varepsilon _k}(\tau )}^{k-1}\Big ) \Vert \vec {v}_1 - \vec {v}_2\Vert _{\vec {X}_{t_0, R}^{1-\varepsilon _k}(\tau )}, \end{aligned} \end{aligned}$$

where $\vec {v}_j = (v_j, \partial _tv_j)$, $j = 1, 2$. Hence, by taking $\tau = \tau \big (\Vert (v_0, v_1)\Vert _{\vec {H}^{1-\varepsilon _k}(B_{R- t_0})}, M_0, M_1\big )> 0$ sufficiently small, the desired claim follows from a standard contraction argument with (4.25) and (4.26). $\square$

#### Proposition 4.3

(stability). Suppose that $\Xi _0$, $u_1$, and $\xi$ satisfy (4.22) and that $v = v(\Xi _0, u_1, \xi )$ is a solution to (4.21) (with $t_0 = 0$) on the cone ${\textbf{C}}_R$ such that

4.27 $$\begin{aligned} \Vert \vec {v}\Vert _{L^\infty ([0, R]; \vec {H}^{1-\varepsilon _k}(B_{R - t}))}&\le M_2 \end{aligned}$$

for some $M_2 \ge 1$. Then, there exists $\delta _* = \delta _*(R, M_0, M_1, M_2) > 0$ such that for any $\widetilde{\Xi }_0$ satisfying

4.28 $$\begin{aligned} \begin{aligned} \Vert \Xi _0 - \widetilde{\Xi }_0 \Vert _{{\mathbb {W}}(R)}&<\delta _*, \\ \Vert \Xi _1(u_1, \xi )\Vert _{\Xi _0, \widetilde{\Xi }_0, R} \le M_1&< \infty , \end{aligned} \end{aligned}$$

where $\widetilde{\Xi }_0 = (\widetilde{\Xi }_{01}, \dots , \widetilde{\Xi }_{0k})$ and ${\mathbb {W}}(R)$ is as in (4.15), there exists $\widetilde{v} = \widetilde{v}(\widetilde{\Xi }_0, u_1, \xi ) \in L^\infty ([0, R]; H^{s}(B_{R - t}) )$, satisfying

4.29 $$\begin{aligned} \begin{aligned} \widetilde{v}(t)&= - \sum _{\ell = 0}^k \sum _{m = 0}^\ell {k\atopwithdelims ()\ell } {\ell \atopwithdelims ()m}\\&\qquad \times \int _0^t {\mathcal {D}}(t - t')\big ( \widetilde{\Xi }_{0m}(t') :\! \Phi _1^{\ell -m} (t')\!: \widetilde{v}^{k- \ell }(t')\big ) dt' \end{aligned} \end{aligned}$$

such that

4.30 $$\begin{aligned} \begin{aligned} \sup _{0 \le t \le R}&\Vert \vec {v}(t) - \vec {\widetilde{v}}(t) \Vert _{\vec {H}^{1-\varepsilon _k}(B_{R-t})}\\&\le C_0(R, M_0, M_1, M_2) \Vert \Xi _0 - \widetilde{\Xi }_0\Vert _{{\mathbb {W}}(R)}, \end{aligned} \end{aligned}$$

where $\vec {v} = (v, \partial _tv)$, $\vec {\widetilde{v}} = (\widetilde{v}, \partial _t\widetilde{v})$, and $\varepsilon _k = 2(k+1) \varepsilon$ is as in (4.19).

#### Proof

Note that Proposition 4.2 with (4.22) and (4.28) guarantees existence of a solution $\widetilde{v} = \widetilde{v}(\widetilde{\Xi }_0, u_1, \xi )$ to (4.29) on a short time interval. Namely, it satisfies

4.31 $$\begin{aligned} \begin{aligned} \widetilde{v}(t)&= - \sum _{\ell = 0}^k \sum _{m = 0}^\ell {k\atopwithdelims ()\ell } {\ell \atopwithdelims ()m}\\&\qquad \times \int _0^t {\mathcal {D}}(t - t')\big ( {\textbf{1}}_{{\textbf{C}}_R} \cdot \widetilde{\Xi }_{0m}(t') :\! \Phi _1^{\ell -m} (t')\!: \widetilde{v}^{k- \ell }(t')\big ) dt' \end{aligned} \end{aligned}$$

on ${\textbf{C}}_R\cap \{0 \le t \le \tau \}$ for some $\tau > 0$, where $\Phi _1 = \Phi _1(u_1, \xi )$ is as in (4.10). From (4.23), we have

4.32 $$\begin{aligned} \Vert \vec {\widetilde{v}}\Vert _{L^\infty _\tau \vec {H}^{1-\varepsilon _k}(B_{R-t})}\lesssim 1. \end{aligned}$$

As for *v*, it satisfies (4.21) (with $t_0 = 0$) on ${\textbf{C}}_R$ (with an additional cutoff function ${\textbf{1}}_{{\textbf{C}}_R}$ as in (4.31)).

Given an interval $I \subset {\mathbb {R}}_+$, let

4.33 $$\begin{aligned} B_I\big (\vec {v}, \vec {\widetilde{v}}\,\big ) = \Vert \vec {v} \Vert _{L^\infty (I; \vec {H}^{1-\varepsilon _k}(B_{R-t}))} + \Vert \vec {\widetilde{v}} \Vert _{L^\infty (I; \vec {H}^{1-\varepsilon _k}(B_{R-t}))} \end{aligned}$$

Then, it follows from (4.27) and (4.32) that

4.34 $$\begin{aligned} B_{[0, \tau ]}\big (\vec {v}, \vec {\widetilde{v}}\,\big ) \le M_2 + C_0' \lesssim M_2 \end{aligned}$$

for $I = [0, \tau ]$. Then, proceeding as in the proof of Proposition 4.2 with (4.21), (4.31), Lemma 2.8, and Sobolev’s inequality with (4.22), (4.28), and (4.34) we have

$$\begin{aligned}&\Vert \vec {v} - \vec {\widetilde{v}} \Vert _{L^\infty _{\tau _0} \vec {H}^{1-\varepsilon _k}(B_{R-t})}\\&\quad \lesssim \tau _0^\theta M_1 \sum _{\ell = 0}^k \sum _{m = 0}^\ell c_{k, \ell , m} B_{[0, \tau ]}\big (\vec {v}, \vec {\widetilde{v}}\, \big )^{k - \ell -1} \\&\qquad \qquad \times \Big \{ B_{[0, \tau ]}\big (\vec {v}, \vec {\widetilde{v}}\,\big ) \Vert \Xi _0 - \widetilde{\Xi }_0 \Vert _{{\mathbb {W}}(R)} + M_0 \Vert \vec {v} - \vec {\widetilde{v}} \Vert _{L^\infty _{\tau _0} \vec {H}^{1-\varepsilon _k}(B_{R-t})}\Big \}\\&\quad \lesssim \tau _0^\theta M_1 M_2^{k-1} \Big \{M_2 \Vert \Xi _0 - \widetilde{\Xi }_0 \Vert _{{\mathbb {W}}(R)} + M_0 \Vert \vec {v} - \vec {\widetilde{v}} \Vert _{L^\infty _{\tau _0} \vec {H}^{1-\varepsilon _k}(B_{R-t})}\Big \} \end{aligned}$$

for any $0 < \tau _0 \le \tau$. Hence, by taking $\tau _0 = \tau _0(M_0, M_1, M_2) > 0$ sufficiently small, we obtain

$$\begin{aligned} \Vert \vec {v} - \vec {\widetilde{v}} \Vert _{L^\infty _{\tau _0} \vec {H}^{1-\varepsilon _k}(B_{R-t})} \le C_1 \Vert \Xi _0 - \widetilde{\Xi }_0 \Vert _{{\mathbb {W}}(R)}. \end{aligned}$$

By repeating the computation on the second interval $I_2 = [\tau _0, 2\tau _0)$, using (4.21) with $t_0 = \tau _0$ and an analogous formulation for $\widetilde{v}$, we have

$$\begin{aligned}&\Vert \vec {v} - \vec {\widetilde{v}} \Vert _{L^\infty (I_2; \vec {H}^{1-\varepsilon _k}(B_{R-t }))} \le C_1 (1+ C_2) \Vert \Xi _0 - \widetilde{\Xi }_0 \Vert _{{\mathbb {W}}(R)}, \end{aligned}$$

provided that $\delta _* = \delta _*(M_2) > 0$ in (4.28) is sufficiently small that

4.35 $$\begin{aligned} B_{I}\big (\vec {v}, \vec {\widetilde{v}}\,\big ) \lesssim M_2 \end{aligned}$$

holds for $I = I_j$, $j = 1, 2$, where $B_I\big (\vec {v}, \vec {\widetilde{v}}\,\big )$ is as in (4.33). Then, by iterating the computation on the *j*th interval $I_j = [(j-1) \tau _0, j\tau _0)$, we obtain

$$\begin{aligned} \Vert \vec {v} - \vec {\widetilde{v}} \Vert _{L^\infty (I_j ; \vec {H}^{1-\varepsilon _k}(B_{R-t}))} \le C_1 \sum _{i = 0}^{j - 1}C_2^i \, \Vert \Xi _0 - \widetilde{\Xi }_0 \Vert _{{\mathbb {W}}(R)}, \end{aligned}$$

provided that $\delta _* = \delta _*(j, M_2) > 0$ in (4.28) is sufficiently small that (4.35) holds for $I = I_i$, $i = 1, \dots , j$.

Note that our choice of $\tau _0 = \tau _0(M_0, M_1, M_2)$ does not depend on $\delta _*$ in (4.28). Thus, by iterating this argument $\sim \frac{R}{\tau _0}$ many times (which requires us to choose $\delta _* = \delta _*\big (\frac{R}{\tau _0}, M_2\big ) = \delta _*(R, M_0, M_1, M_2) > 0$ sufficiently small), we obtain (4.30). This concludes the proof of Proposition 4.3. $\square$

### Convergence of the enhanced Gibbs measures

Let $\Xi _0(\phi )$ be the first enhanced data set defined in (4.12). By viewing $\Xi _0$ as a map sending $\phi$ to $\Xi _0(\phi )$, we consider the pushforward measure $\nu _L$ of the *L*-periodic $\Phi ^{k+1}_2$-measure $\rho _L$ under the map $\Xi _0$. Namely, $\nu _L$ is given by

4.36 $$\begin{aligned} \nu _L = (\Xi _0)_\# \rho _L. \end{aligned}$$

See also (1.36). In the following, we refer to $\nu _L$ as the *enhanced Gibbs measure*. In this subsection, we study convergence properties of the enhanced Gibbs measure $\nu _L$, which plays an essential role in establishing global well-posedness of the hyperbolic $\Phi ^{k+1}_2$-model (4.1) on ${\mathbb {R}}^2$ (as a limit of the *L*-periodic hyperbolic $\Phi ^{k+1}_2$-model (4.2)) and invariance of the Gibbs measure $\vec {\rho }_\infty$ constructed in Theorem 1.2 (i).

#### Proposition 4.4

As probability measures on ${\mathbb {W}}= \big (C({\mathbb {R}}_+; W^{-\varepsilon , p}_\mu ({\mathbb {R}}^2))\big )^{\otimes k}$, the family $\{\nu _L\}_{L \ge 1}$ is tight, and thus there exists a sequence $\{\nu _{L_j}\}_{j \in {\mathbb {N}}}$ of the $L_j$-periodic enhanced Gibbs measures that converges weakly to some limit $\nu _\infty$. Moreover, $\nu _{L_j}$ also converges to the same limit $\nu _\infty$ in the Wasserstein-1 metric.

#### Proof

By the definition (4.36), we have $\nu _L = {{\,\textrm{Law}\,}}(\Xi _0(\phi ))$ with ${{\,\textrm{Law}\,}}(\phi ) = \rho _L$. Let $X_L$ be the solution to the *L*-periodic SNLH (3.42) with ${{\,\textrm{Law}\,}}(X_{0, L}) = \rho _L$. Then, by the invariance (3.47) of $\rho _L$ under (3.42), we have ${{\,\textrm{Law}\,}}(X_L(1)) = \rho _L$, and thus $\nu _L = {{\,\textrm{Law}\,}}(\Xi _0(X_L(1))$, where

4.37 $$\begin{aligned} \Xi _0(X_L(1) ) = \big ( {\mathcal {S}}(t) X_L(1), \, :\!({\mathcal {S}}(t) X_L(1) )^2\!: \, , \dots , \, :\!({\mathcal {S}}(t) X_L(1))^k\!: \big ) . \end{aligned}$$

Hence, tightness of $\{ \nu _L\}_{L \ge 1}$ follows once we prove tightness of $\big \{ {{\,\textrm{Law}\,}}(\Xi _0(X_L(1))\big \}_{L\ge 1}$. Once we have tightness of $\{ \nu _L\}_{L \ge 1}$, the Prokhorov theorem (Lemma 2.18) implies that there exists a subsequence of $\{\nu _L\}_{L \ge 1}$ converging weakly to some limit $\nu _\infty$. Moreover, noting that the metric $d_{\mathbb {W}}$ on ${\mathbb {W}}$ defined in (4.14) is bounded, it follows from Lemma 2.15 and Remark 2.16 that the same subsequence of $\{ \nu _L\}_{L \ge 1}$ converges to the same limit $\nu _\infty$ in the Wasserstein-1 metric, which shows the second claim in Proposition 4.4. Therefore, we focus on proving tightness of $\big \{ {{\,\textrm{Law}\,}}(\Xi _0(X_L(1))\big \}_{L\ge 1}$ in the following.

By the decomposition (3.43), we have

4.38 $$\begin{aligned} {\mathcal {S}}(t) X_L(1) = {\mathcal {S}}(t) Y_L(1) + {\mathcal {S}}(t) Z_L(1). \end{aligned}$$

Then, from (2.57), we have

4.39 $$\begin{aligned} :\!({\mathcal {S}}(t) X_L(1))^\ell \!: \, = \sum _{m=0}^\ell \left( {\begin{array}{c}\ell \\ m\end{array}}\right) ({\mathcal {S}}(t)Y_L(1) )^{\ell -m}:\!({\mathcal {S}}(t)Z_L(1))^m\!: \end{aligned}$$

for $\ell = 1, \dots , k$. When $m\ne 0$, Lemma 2.11 (ii) yields

4.40 $$\begin{aligned} \begin{aligned}&\Vert ({\mathcal {S}}(t)Y_L(1) )^{\ell -m}:\!({\mathcal {S}}(t)Z_L(1))^m\!: \Vert _{W^{-\varepsilon , p}_\mu }\\&\quad \lesssim \Vert :\!({\mathcal {S}}(t)Z_L(1))^m\!: \Vert _{W^{-\varepsilon , p_1}_\mu } \Vert ({\mathcal {S}}(t)Y_L(1) )^{\ell -m}\Vert _{W^{\varepsilon , p_2}_{\frac{\mu }{2^\delta }}} \end{aligned} \end{aligned}$$

for $1< p_1, p_2 < \infty$ with $p_1 \gg 1$, satisfying $\frac{1}{p} \le \frac{1}{p_1} + \frac{1}{p_2} \le \frac{1}{p} + \frac{\varepsilon }{2}$. When $m = 0$, we simply use the bound:

$$\begin{aligned} \Vert ({\mathcal {S}}(t)Y_L(1) )^{\ell } \Vert _{W^{-\varepsilon , p}_\mu } \lesssim \Vert ({\mathcal {S}}(t)Y_L(1) )^{\ell }\Vert _{W^{\varepsilon , p_2}_{\frac{\mu }{2^\delta }}} \end{aligned}$$

for $p_2 \ge p$ and apply the fractional Leibniz rule (Lemma 2.11 (i)); see (4.58) below.

$\bullet$ **Part 1:** We first estimate $:\!({\mathcal {S}}(t)Z_L(1))^m\!: \,$, $m = 1, \dots , k$. Recall from (2.5) with (1.23) that

$$\begin{aligned} {\mathcal {S}}(t)&= \partial _t{\mathcal {D}}(t) + {\mathcal {D}}(t) = e^{-\frac{t}{2}} \cos ( t [\hspace{-0.6mm}[ \nabla ]\hspace{-0.6mm}] ) + \frac{1}{2} e^{-\frac{t}{2}} \frac{\sin ( t[\hspace{-0.6mm}[ \nabla ]\hspace{-0.6mm}] )}{[\hspace{-0.6mm}[ \nabla ]\hspace{-0.6mm}]}, \end{aligned}$$

where

4.41 $$\begin{aligned} [\hspace{-0.6mm}[ \nabla ]\hspace{-0.6mm}] = \sqrt{\tfrac{3}{4} - \Delta }. \end{aligned}$$

Given $\lambda \in {\mathbb {Z}}^2_L$, we set

4.42 $$\begin{aligned} \widehat{{\mathcal {S}}(t)}(\lambda ) = e^{-\frac{t}{2}} \cos ( t [\hspace{-0.6mm}[ 2\pi \lambda ]\hspace{-0.6mm}] ) + \frac{1}{2} e^{-\frac{t}{2}} \frac{\sin ( t[\hspace{-0.6mm}[ 2\pi \lambda ]\hspace{-0.6mm}] )}{[\hspace{-0.6mm}[ 2\pi \lambda ]\hspace{-0.6mm}]}, \end{aligned}$$

where $[\hspace{-0.6mm}[ x ]\hspace{-0.6mm}] = \sqrt{\frac{3}{4} + |x|^2}$. From (3.44), we have

4.43 $$\begin{aligned} {\mathcal {S}}(t) Z_L(1)&= \sqrt{2}\int _{0}^1 {\mathcal {S}}(t) P(1-t') \xi _L(dt'), \end{aligned}$$

where $\xi _L$ is as in (4.4) and (4.5).

We first consider the case $m = 1$. From (4.43) with (4.4) and (4.5), we have

4.44 $$\begin{aligned} \begin{aligned} {\mathbb {E}}&\Big [ \big | \langle \nabla \rangle ^{-\varepsilon }\big ( \phi _j {\mathcal {S}}(t) Z_L(1) \big ) (x) \big |^2 \Big ] \\&=\frac{2}{L^2} {\mathbb {E}}\Bigg [ \bigg | \sum _{n\in {\mathbb {Z}}^2} \int _{{\mathbb {R}}^2} \frac{1}{\langle \eta \rangle ^\varepsilon } \widehat{ {\mathcal {S}}(t)}\big (\tfrac{n}{L}\big )\\&\qquad \times \int _{0}^1 e^{-(1-t') \langle \frac{2\pi n}{L} \rangle ^2 } \widehat{\xi }_L\big ( \tfrac{n}{L}, dt' \big ) \widehat{\phi }_j \big (\eta - \tfrac{n}{L}\big ) e^{2 \pi i\eta \cdot x} d\eta \bigg |^2 \Bigg ]. \end{aligned} \end{aligned}$$

Recall that $\big \{ \widehat{\xi }_L\big (\cdot , \tfrac{n}{L} \big ) \big \}_{n \in {\mathbb {Z}}^2}$ is a family of independent temporal white noises conditioned that $\widehat{\xi }_L\big (- \tfrac{n}{L}\big ) = \overline{\widehat{\xi }_L\big ( \tfrac{n}{L}\big )}$, $n \in {\mathbb {Z}}^2$, and thus the temporal stochastic integrals in (4.44) are standard Wiener integrals. Hence, we have

4.45 $$\begin{aligned} (4.44)&\lesssim \frac{e^{-t} }{L^2} \sum _{n\in {\mathbb {Z}}^2} \frac{1}{ \langle \frac{n}{L} \rangle ^2 } \bigg | \int _{{\mathbb {R}}^2} \frac{1}{\langle \eta \rangle ^\varepsilon } \widehat{\phi }_j \big (\eta - \tfrac{n}{L}\big ) e^{2\pi i\eta \cdot x} d\eta \bigg |^2. \end{aligned}$$

From the triangle inequality $\langle \frac{n}{L} \rangle ^\varepsilon \lesssim \langle \eta \rangle ^\varepsilon \langle \eta - \tfrac{n}{L} \rangle ^\varepsilon$ with (2.47) and (2.48), we have

4.46 $$\begin{aligned} \begin{aligned} \bigg | \int \frac{1}{\langle \eta \rangle ^\varepsilon } \widehat{\phi }_j \big (\eta - \tfrac{n}{L}\big ) e^{2\pi i\eta \cdot x} d\eta \bigg |&\lesssim \frac{1}{\langle \frac{n}{L} \rangle ^\varepsilon } \int _{{\mathbb {R}}^2} \big |\langle \eta - \tfrac{n}{L} \rangle ^\varepsilon \widehat{\phi }_j \big (\eta - \tfrac{n}{L}\big )\big | d\eta \\&\lesssim \frac{1}{\langle \frac{n}{L} \rangle ^\varepsilon } . \end{aligned} \end{aligned}$$

On the other hand, we have

4.47 $$\begin{aligned} \int _{{\mathbb {R}}^2} \frac{1}{\langle \eta \rangle ^\varepsilon } \widehat{\phi }_j \big (\eta - \tfrac{n}{L}\big ) e^{2\pi i\eta \cdot x} d\eta = \int _{{\mathbb {R}}^2} G_\varepsilon (x-y) \phi _j (y) e^{2\pi i \frac{n}{L}\cdot y} dy, \end{aligned}$$

where $G_\varepsilon$ is the kernel of the Bessel potential $\langle \nabla \rangle ^{-\varepsilon }$ of order $\varepsilon$. Recall the following bound on the kernel $G_\varepsilon (x)$ (see [38, Proposition 1.2.5]):

4.48 $$\begin{aligned} |G_\varepsilon (x)| \lesssim {\left\{ \begin{array}{ll} e^{-\frac{|x|}{2}}, &  \text {for } |x| \ge 2,\\ |x|^{\varepsilon - 2}, &  \text {for } |x| < 2, \end{array}\right. } \end{aligned}$$

since $0< \varepsilon < 2$. Thus, it follows from (4.47) and (4.48) with (2.16) that

4.49 $$\begin{aligned} \begin{aligned} \bigg |&\int _{{\mathbb {R}}^2} \frac{1}{\langle \eta \rangle ^\varepsilon } \widehat{\phi }_j \big (\eta - \tfrac{n}{L}\big ) e^{2\pi i\eta \cdot x} d\eta \bigg | \le \int _{{\mathbb {R}}^2} |G_\varepsilon (x-y)| |\phi _j (y)| dy\\&= \int _{|x-y|\ge 2} |G_\varepsilon (x-y)| |\phi _j (y)| dy + \int _{|x-y|< 2} |G_\varepsilon (x-y)| |\phi _j (y)| dy\\&\lesssim \int _{|x-y|\ge 2} \frac{1}{|x- y|^{M+2}} \frac{1}{\langle \frac{y}{2^j} \rangle ^M} dy + \int _{|x-y|< 2}\frac{1}{|x- y|^{2-\varepsilon }} \frac{1}{\langle \frac{x-y}{2^j} \rangle ^M} \frac{1}{\langle \frac{y}{2^j} \rangle ^M} dy\\&\lesssim \frac{1}{\langle \frac{x}{2^j} \rangle ^M} \end{aligned} \end{aligned}$$

for any $M \gg 1$, where, in the third step, we used the fact that $\langle \frac{x-y}{2^j} \rangle \sim 1$ for $| x - y| \le 2$. Hence, by interpolating (4.46) and (4.49), we obtain from (4.44), (4.45), and a Riemann sum approximation that

4.50 $$\begin{aligned} \begin{aligned} {\mathbb {E}}\Big [ \big |&\langle \nabla \rangle ^{-\varepsilon }\big ( \phi _j {\mathcal {S}}(t) Z_L(1) \big ) (x) \big |^2 \Big ] \lesssim \frac{e^{-t} }{L^2} \sum _{n\in {\mathbb {Z}}^2} \frac{1}{ \langle \frac{n}{L} \rangle ^{2+2\varepsilon -} } \frac{1}{\langle \frac{x}{2^j} \rangle ^M} \\&\lesssim \frac{e^{-t} }{\langle \frac{x}{2^j} \rangle ^M} \int _{{\mathbb {R}}^2} \frac{1}{ \langle y \rangle ^{2+2\varepsilon -} } \lesssim \frac{e^{-t} }{\langle \frac{x}{2^j} \rangle ^M} \end{aligned} \end{aligned}$$

for any $M \gg 1$, uniformly in $L \ge 1$.

Given any finite $q \ge p \ge 2$, it follows from (2.22), Minkowski’s integral inequality, the Wiener chaos estimate (Lemma 2.12), and (4.50) that

4.51 $$\begin{aligned} \begin{aligned} \big \Vert \Vert {\mathcal {S}}(t)&Z_L(1) \Vert _{W^{-\varepsilon , p}_\mu } \big \Vert _{L^q(\Omega )} \le \Big \Vert w_{\frac{\mu }{p}} (2^{j}) \big \Vert \langle \nabla \rangle ^{-\varepsilon }\big (\phi _j {\mathcal {S}}(t) Z_L(1)\big )(x)\big \Vert _{L^q(\Omega )} \Big \Vert _{\ell ^p_jL^p_x }\\&\lesssim q^\frac{1}{2} \Big \Vert w_{\frac{\mu }{p}} (2^{j}) \big \Vert \langle \nabla \rangle ^{-\varepsilon }\big (\phi _j {\mathcal {S}}(t) Z_L(1)\big )(x)\big \Vert _{L^2(\Omega )} \Big \Vert _{\ell ^p_jL^p_x }\\&\lesssim q^\frac{1}{2} e^{-\frac{t}{2}} \bigg \Vert w_{\frac{\mu }{p}} (2^{j}) \frac{1}{\langle \frac{x}{2^j} \rangle ^\frac{M}{2}} \bigg \Vert _{\ell ^p_jL^p_x } \lesssim q^\frac{1}{2} e^{-\frac{t}{2}} \Vert w_{\frac{\mu }{p}} (2^{j}) 2^{\frac{2}{p} j} \Vert _{\ell ^p_j }\\&\lesssim q^\frac{1}{2} e^{-\frac{t}{2}}, \end{aligned} \end{aligned}$$

uniformly in $$L \ge 1$$.

From (4.42) and the mean value theorem, we have

4.52 $$\begin{aligned} |\widehat{{\mathcal {S}}(t + h)}(\lambda ) - \widehat{{\mathcal {S}}(t)}(\lambda )| \lesssim e^{-\frac{t}{2}} \min \big (1, |h| \langle \lambda \rangle \big )^\kappa \end{aligned}$$

for any $\lambda \in {\mathbb {Z}}^2_L$ and $0 \le \kappa \le 1$. Then, by repeating an analogous computation with (4.52), we obtain

4.53 $$\begin{aligned} \begin{aligned} \big \Vert \Vert {\mathcal {S}}(t+h) Z_L(1) - {\mathcal {S}}(t) Z_L(1) \Vert _{W^{-\varepsilon , p}_\mu } \big \Vert _{L^q(\Omega )}&\lesssim q^\frac{1}{2} e^{-\frac{t}{2}}|h|^{\kappa } \end{aligned} \end{aligned}$$

for any $q \ge 1$ and $0< \kappa < \varepsilon$, uniformly in $L \ge 1$.

Let $T > 0$. In view of (4.51) and (4.52), by applying Kolmogorov’s continuity criterion ( [5, Theorem 8.2]), we see that ${\mathcal {S}}(t)Z_L(1) \in C^\alpha ([0, T]; W^{-\varepsilon , p}_\mu ({\mathbb {R}}^2))$ for some small $\alpha >0$. Moreover, the $\alpha$-Hölder semi-norms of $\{ {\mathcal {S}}(t)Z_L(1): t\in [0,T] \}$ are uniformly (in *L*) bounded in $L^q(\Omega )$ for any finite $q\ge 1$. Therefore, by noting that the discussion above holds for any small $\varepsilon > 0$ and any $\mu > 0$, it follows from the Arzelà-Ascoli theorem with Lemma 2.2 that the family $\{ {\mathcal {S}}(t)Z_L(1) \}_{L\ge 1}$ in $C([0, T]; W^{-\varepsilon , p}_\mu ({\mathbb {R}}^2))$ is tight^29^ for any finite *T*, and hence $\{ {\mathcal {S}}(t)Z_L(1) \}_{L\ge 1}$ in $C({\mathbb {R}}_+; W^{-\varepsilon , p}_\mu ({\mathbb {R}}^2))$ is tight.

#### Remark 4.5

By repeating the argument above for $e^{\frac{t}{2}}{\mathcal {S}}(t)Z_L(1)$, we see that $e^{\frac{t}{2}}{\mathcal {S}}(t)Z_L(1)$ is almost surely $\alpha$-Hölder continuous in time. In particular, it grows at most polynomially in time. From this observation, we conclude that ${\mathcal {S}}(t)Z_L(1)$ is almost surely bounded on ${\mathbb {R}}_+$ with values in $W^{-\varepsilon , p}_\mu ({\mathbb {R}}^2)$.

We now study the higher powers $:\!({\mathcal {S}}(t)Z_L(1))^m\!: \,$, $m = 2, \dots , k$. From Lemma 2.13 and (4.43) with (4.4) and (4.5), we have^30^

4.54 $$\begin{aligned} \begin{aligned}&{\mathbb {E}}\big [ :\!({\mathcal {S}}(t) Z_L(1))^m(y)\!: \, :\!({\mathcal {S}}(t) Z_L(1))^m(y')\!:\big ]\\&\quad = m! \Big \{{\mathbb {E}}\big [ ({\mathcal {S}}(t) Z_L(1))(y) \cdot ({\mathcal {S}}(t) Z_L(1))(y')\big ]\Big \}^m\\&\quad \sim \frac{1}{L^{2m}} \sum _{n \in {\mathbb {Z}}^2} e^{2\pi i \frac{n}{L} \cdot (y - y')} \sum _{\begin{array}{c} n_1, \dots , n_m \in {\mathbb {Z}}^2\\ n = n_1 + \cdots + n_m \end{array}} \prod _{j = 1}^m\bigg \{\big (\widehat{ {\mathcal {S}}(t)}\big (\tfrac{n_j}{L}\big )\big )^2 \frac{1 - e^{-2\langle \frac{2\pi n_j}{L} \rangle ^2}}{2\langle \frac{2\pi n_j}{L} \rangle ^2}\bigg \}. \end{aligned} \end{aligned}$$

Then, by (4.54) and interpolating (4.46) and (4.49), we have

$$\begin{aligned} {\mathbb {E}}&\Big [ \big | \langle \nabla \rangle ^{-\varepsilon }\big ( \phi _j :\!({\mathcal {S}}(t) Z_L(1))^m\!: \big ) (x) \big |^2 \Big ] \\&= \int _{{\mathbb {R}}^2}\int _{{\mathbb {R}}^2}\frac{1}{\langle \eta \rangle ^\varepsilon }\frac{1}{\langle \eta ' \rangle ^\varepsilon } \int _{{\mathbb {R}}^2}\int _{{\mathbb {R}}^2}\phi _j(y)\phi _j(y')\\&\quad \times {\mathbb {E}}\big [ :\!({\mathcal {S}}(t) Z_L(1))^m(y)\!: \, :\!({\mathcal {S}}(t) Z_L(1))^m(y')\!:\big ]\\&\quad \times e^{-2\pi i \eta \cdot y}e^{-2\pi i \eta '\cdot y'}dy dy' e^{2\pi i \eta \cdot x}e^{2\pi i \eta '\cdot x} d\eta d\eta '\\&\sim \frac{1}{L^{2m}} \sum _{n \in {\mathbb {Z}}^2} \bigg (\int _{{\mathbb {R}}^2}\frac{1}{\langle \eta \rangle ^\varepsilon } \widehat{\phi }_j\big (\eta - \tfrac{n}{L}\big ) e^{2\pi i \eta \cdot x}d\eta \bigg ) \bigg ( \int _{{\mathbb {R}}^2} \frac{1}{\langle \eta ' \rangle ^\varepsilon }\widehat{\phi }_j\big (\eta ' + \tfrac{n}{L}\big ) e^{2\pi i \eta '\cdot x} d\eta '\bigg )\\&\quad \times \sum _{\begin{array}{c} n_1, \dots , n_m \in {\mathbb {Z}}^2\\ n = n_1 + \cdots + n_m \end{array}} \prod _{j = 1}^m\bigg \{\big (\widehat{ {\mathcal {S}}(t)}\big (\tfrac{n_j}{L}\big )\big )^2 \frac{1 - e^{-2\langle \frac{2\pi n_j}{L} \rangle ^2}}{2\langle \frac{2\pi n_j}{L} \rangle ^2}\bigg \}\\&\lesssim \frac{e^{-mt}}{\langle \frac{x}{2^j} \rangle ^M} \frac{1}{L^{2m}} \sum _{n \in {\mathbb {Z}}^2} \frac{1}{ \langle \frac{n}{L} \rangle ^{2\varepsilon -} } \sum _{\begin{array}{c} n_1, \dots , n_m \in {\mathbb {Z}}^2\\ n = n_1 + \cdots + n_m \end{array}} \prod _{j = 1}^m \frac{1}{\langle \frac{ n_j}{L} \rangle ^2}. \end{aligned}$$

By a Riemann sum approximation, we then obtain

4.55 $$\begin{aligned} \begin{aligned} {\mathbb {E}}&\Big [ \big | \langle \nabla \rangle ^{-\varepsilon }\big ( \phi _j :\!({\mathcal {S}}(t) Z_L(1))^m\!: \big ) (x) \big |^2 \Big ] \\&\lesssim \frac{e^{-mt}}{\langle \frac{x}{2^j} \rangle ^M} \int _{({\mathbb {R}}^2)^m} \frac{1}{ \langle y_1 + \cdots + y_m \rangle ^{2\varepsilon -} } \prod _{j = 1}^m \frac{1}{\langle y_j \rangle ^2} dy_1 \cdots dy_m\\&\lesssim \frac{e^{-mt}}{\langle \frac{x}{2^j} \rangle ^M} \end{aligned} \end{aligned}$$

for any $M \gg 1$, uniformly in $L \ge 1$.

We now proceed as in (4.51). Namely, given any finite $q \ge p \ge 2$, it follows from (2.22), Minkowski’s integral inequality, the Wiener chaos estimate (Lemma 2.12), and (4.55) that

4.56 $$\begin{aligned} \begin{aligned} \big \Vert \Vert :\!(S(t)&Z_L(1))^m\!: \Vert _{W^{-\varepsilon , p}_\mu } \big \Vert _{L^q(\Omega )}\\&\lesssim q^\frac{m}{2} \Big \Vert w_{\frac{\mu }{p}} (2^{j}) \big \Vert \langle \nabla \rangle ^{-\varepsilon }\big (\phi _j :\!(S(t) Z_L(1))^m\!: \big )(x)\big \Vert _{L^2(\Omega )} \Big \Vert _{\ell ^p_jL^p_x }\\&\lesssim q^\frac{m}{2} e^{-\frac{m}{2}t} \bigg \Vert w_{\frac{\mu }{p}} (2^{j}) \frac{1}{\langle \frac{x}{2^j} \rangle ^\frac{M}{2}} \bigg \Vert _{\ell ^p_jL^p_x } \lesssim q^\frac{m}{2} e^{-\frac{m}{2}t} \Vert w_{\frac{\mu }{p}} (2^{j}) 2^{\frac{2}{p} j} \Vert _{\ell ^p_j }\\&\lesssim q^\frac{m}{2} e^{-\frac{m}{2}t}, \end{aligned} \end{aligned}$$

uniformly in $$L \ge 1$$.

We briefly discuss how to handle the time increment $:\!({\mathcal {S}}(t)Z_L(1))^m\!: - :\!({\mathcal {S}}(t)Z_L(1))^m\!: \,$. The main idea is to proceed as in the second half of the proof of Proposition 2.1 in [43]. Given $h \in {\mathbb {R}}$, define the difference operator $\delta _h$ by setting^31^

$$\begin{aligned} \delta _h F(t) = F(t+h) - F(t).\end{aligned}$$

Then, by Lemma 2.13, we have

$$\begin{aligned} \frac{1}{m!} {\mathbb {E}}&\big [ \delta _h:\!({\mathcal {S}}(t) Z_L(1))^m(y)\!: \, \delta _h :\!({\mathcal {S}}(t) Z_L(1))^m(y')\!:\big ]\\&= \Big \{{\mathbb {E}}\big [ ({\mathcal {S}}(t+h) Z_L(1))(y) \cdot ({\mathcal {S}}(t+h) Z_L(1))(y')\big ]\Big \}^m\\&\quad - \Big \{{\mathbb {E}}\big [ ({\mathcal {S}}(t) Z_L(1))(y) \cdot ({\mathcal {S}}(t+h) Z_L(1))(y')\big ]\Big \}^m\\&\quad - \Big \{{\mathbb {E}}\big [ ({\mathcal {S}}(t+h) Z_L(1))(y) \cdot ({\mathcal {S}}(t) Z_L(1))(y')\big ]\Big \}^m\\&\quad + \Big \{{\mathbb {E}}\big [ ({\mathcal {S}}(t) Z_L(1))(y) \cdot ({\mathcal {S}}(t) Z_L(1))(y')\big ]\Big \}^m\\&= {\mathbb {E}}\big [ \delta _h ({\mathcal {S}}(t) Z_L(1))(y) \cdot ({\mathcal {S}}(t+h) Z_L(1))(y')\big ]\\&\quad \quad \times \sum _{j = 0}^{m-1} \Big \{{\mathbb {E}}\big [ ({\mathcal {S}}(t+h) Z_L(1))(y) \cdot ({\mathcal {S}}(t+h) Z_L(1))(y')\big ]\Big \}^{m-j-1}\\&\quad \quad \quad \quad \times \Big \{{\mathbb {E}}\big [ ({\mathcal {S}}(t) Z_L(1))(y) \cdot ({\mathcal {S}}(t+h) Z_L(1))(y')\big ]\Big \}^{j}\\&\quad - {\mathbb {E}}\big [ \delta _h ({\mathcal {S}}(t) Z_L(1))(y) \cdot ({\mathcal {S}}(t) Z_L(1))(y')\big ]\\&\quad \quad \times \sum _{j = 0}^{m-1} \Big \{{\mathbb {E}}\big [ ({\mathcal {S}}(t+h) Z_L(1))(y) \cdot ({\mathcal {S}}(t) Z_L(1))(y')\big ]\Big \}^{m-j-1}\\&\quad \quad \quad \quad \times \Big \{{\mathbb {E}}\big [ ({\mathcal {S}}(t) Z_L(1))(y) \cdot ({\mathcal {S}}(t) Z_L(1))(y')\big ]\Big \}^{j} \end{aligned}$$

Note that in each term on the right-hand side above, the difference operator $\delta _h$ appears exactly once. Then, with (4.52), we can repeat the computation above for $:\!({\mathcal {S}}(t)Z_L(1))^m\!: \,$ and obtain

4.57 $$\begin{aligned} \begin{aligned} \big \Vert \Vert \delta _h :\!({\mathcal {S}}(t+h) Z_L(1))^m\!: \Vert _{W^{-\varepsilon , p}_\mu } \big \Vert _{L^q(\Omega )}&\lesssim q^\frac{m}{2} e^{-\frac{m}{2}t}|h|^{\kappa } \end{aligned} \end{aligned}$$

for any $q \ge 1$ and $0< \kappa < \varepsilon$, uniformly in $L \ge 1$.

Therefore, applying Kolmogorov’s continuity criterion with (4.56) and (4.57) and arguing as in the $m = 1$ case with Lemma 2.2 and the Arzelà-Ascoli theorem, we conclude that the family $\{:\!({\mathcal {S}}(t)Z_L(1))^m\!:\}_{L\ge 1}$ in $C({\mathbb {R}}_+; W^{-\varepsilon , p}_\mu ({\mathbb {R}}^2))$ is tight.

$\bullet$ **Part 2:** Next, we consider $({\mathcal {S}}(t)Y_L(1) )^m$, $m = 1, \dots , k$. By the fractional Leibniz rule (Lemma 2.11 (i)), (2.24), and Lemma 2.6, we have

4.58 $$\begin{aligned} \begin{aligned} \Vert ({\mathcal {S}}(t)Y_L(1) )^m\Vert _{L^\infty _t W^{\varepsilon , p}_{\frac{\mu }{2^\delta }}}&\lesssim \Vert {\mathcal {S}}(t)Y_L(1) \Vert ^m_{L^\infty _t W^{\varepsilon , mp }_{\mu _1}} \lesssim \Vert {\mathcal {S}}(t)Y_L(1) \Vert ^m_{L^\infty _t H^{1 - \frac{2}{mp} + \varepsilon }_{\mu _2}} \\&\lesssim \sup _{t \in {\mathbb {R}}_+} e^{-\frac{m}{4} t} \Vert Y_L(1) \Vert ^m _{H^{1 - \frac{2}{mp} + \varepsilon }_{\mu _3}} \le \Vert Y_L(1) \Vert ^m _{H^{1 - \frac{2}{mp} + \varepsilon }_{\mu _3}} \end{aligned} \end{aligned}$$

for some $0< \mu _3< \mu _2< \mu _1 < \mu$. Here, we take $\varepsilon > 0$ sufficiently small such that $1 - \frac{2}{mp} + \varepsilon < 1$. It follows from Proposition 3.3 with (3.7) and (3.29), Lemma 2.12, and [60, Theorem 5.1]^32^ that, given any finite $q\ge 1$, the *q*th moment of the right-hand side of (4.58) is bounded, uniformly in $L \ge 1$.

A similar computation with (4.52) (but with $\lambda \in {\mathbb {R}}^2$) yields

4.59 $$\begin{aligned} \begin{aligned} \Vert \delta _h ({\mathcal {S}}(t)Y_L(1) )^m\Vert _{L^\infty _t W^{\varepsilon , p}_{\frac{\mu }{2^\delta }}} \lesssim |h|^\kappa \Vert Y_L(1) \Vert ^m _{H^{1 - \frac{2}{mp} + \varepsilon +\kappa }_{\mu _3}} . \end{aligned} \end{aligned}$$

Then, as long as $\varepsilon , \kappa > 0$ are sufficiently small such that $1 - \frac{2}{mp} + \varepsilon + \kappa < 1$, Proposition 3.3 with (3.7) and (3.29), Lemma 2.12, and [60, Theorem 5.1]^33^ that, given any finite $q\ge 1$, the *q*th moment of the right-hand side of (4.59) is bounded, uniformly in $L \ge 1$. Therefore, applying Kolmogorov’s continuity criterion with (4.58) and (4.59) and arguing as in the $m = 1$ case with Lemma 2.2 and the Arzelà-Ascoli theorem, we conclude that the family $\{ ({\mathcal {S}}(t)Y_L(1) )^m\}_{L\ge 1}$ in $C({\mathbb {R}}_+; W^{\varepsilon , p}_{\frac{\mu }{2^\delta }}({\mathbb {R}}^2))$ is tight.

$\bullet$ **Part 3:** Finally, from (4.39), we have

4.60 $$\begin{aligned} \begin{aligned} \delta _h :\!({\mathcal {S}}(t) X_L(1))^\ell \!: \,&= \sum _{m=0}^\ell \left( {\begin{array}{c}\ell \\ m\end{array}}\right) \delta _h ({\mathcal {S}}(t)Y_L(1) )^{\ell -m}\cdot :\!({\mathcal {S}}(t+h)Z_L(1))^m\!: \\&\quad + \sum _{m=0}^\ell \left( {\begin{array}{c}\ell \\ m\end{array}}\right) ({\mathcal {S}}(t)Y_L(1) )^{\ell -m}\cdot \delta _h :\!({\mathcal {S}}(t)Z_L(1))^m\!: . \end{aligned} \end{aligned}$$

By applying Lemma 2.11 (ii) as in (4.40), we conclude from (4.51), (4.53), (4.56), (4.57). (4.58), and (4.59) with Kolmogorov’s continuity criterion ( [5, Theorem 8.2]), Lemma 2.2, and the Arzelà-Ascoli theorem that the family $\{:\!({\mathcal {S}}(t)X_L(1))^m\!:\}_{L\ge 1}$ in $C({\mathbb {R}}_+; W^{-\varepsilon , p}_\mu ({\mathbb {R}}^2))$ is tight. Namely, as probability measures on ${\mathbb {W}}= C({\mathbb {R}}_+; W^{-\varepsilon , p}_\mu ({\mathbb {R}}^2))^{\otimes k}$, the family $\{\nu _L\}_{L\in {\mathbb {N}}}$ is tight. $\square$

Let ${\mathcal {A}}$ be the index set such that Theorem 1.2 (i) holds. Namely, that the *L*-periodic $\Phi ^{k+1}_2$-measures $\rho _{L}$, $L \in {\mathcal {A}}$ converges weakly to a limiting $\Phi ^{k+1}_2$-measure $\rho _\infty$ on ${\mathbb {R}}^2$. Note that Proposition 4.4 also applies to the family $\{ \nu _L\}_{L \in {\mathcal {A}}}$, showing that there exists a subsequence which converges, weakly and also in the Wasserstein-1 metric, to *some* limit $\nu _\infty$. The next proposition identifies this limiting probability measure $\nu _\infty$.

#### Proposition 4.6

Let the index set ${\mathcal {A}}\subset {\mathbb {N}}$ be as in Theorem 1.2 (i). Then, as probability measures on ${\mathbb {W}}= \big (C({\mathbb {R}}_+; W^{-\varepsilon , p}_\mu ({\mathbb {R}}^2))\big )^{\otimes k}$, the entire sequence $\{\nu _L\}_{L\in {\mathcal {A}}}$ of the *L*-periodic enhanced Gibbs measures converges, weakly and also in the Wasserstein-1 metric, to the unique limit

4.61 $$\begin{aligned} \nu _\infty = (\Xi _0)_\# \rho _\infty . \end{aligned}$$

#### Proof

In the following, we only consider the values of *L* belonging to ${\mathcal {A}}= \{ L_j: j \in {\mathbb {N}}\} \subset {\mathbb {N}}$. For simplicity of notation, we drop the subscript and simply write $L \rightarrow \infty$ with the understanding that each *L* belongs to ${\mathcal {A}}$.

In view of the tightness of $\{\nu _L\}_{L\in {\mathcal {A}}}$ (Proposition 4.4) and Lemma 2.15, we only need to show that the limit in law of $\{\nu _L\}_{L\in {\mathcal {A}}}$ is unique.

Let $X_{L} = Y_{L} + Z_{L}$ be the solution to the *L*-periodic SNLH (3.42), where $Y_{L}$ and $Z_{L}$ satisfy (3.45) and (3.44) with the Gibbsian initial data $X_{0, L}$ with ${{\,\textrm{Law}\,}}(X_{0, L}) = \rho _{L}$. Here, we assume that $X_{0, L}$ is independent of the noise $\xi _L$. In view of the weak convergence of $\rho _L$ to $\rho _\infty$ as $L \rightarrow \infty$ (with *L*’s belonging to ${\mathcal {A}}$) established in Sect. 3.3, it follows from the Skorokhod representation theorem (Lemma 2.19) that there exist a probability measure $\widetilde{\mathbb {P}}$ (on a new probability space) and random variables $\widetilde{X}_{0, L}$ with ${{\,\textrm{Law}\,}}(\widetilde{X}_{0, L}) = \rho _{L}$, $L \in {\mathcal {A}}$, such that $\widetilde{X}_{0, L}$ converges $\widetilde{\mathbb {P}}$-almost surely to some $\widetilde{X}_{0, \infty }$, with ${{\,\textrm{Law}\,}}(\widetilde{X}_{0, \infty }) = \rho _{\infty }$, in $B^{-\varepsilon , \mu }_{p_0, p_0}({\mathbb {R}}^2)$ for any $\varepsilon > 0$, finite $p_0 \ge 1$, and $\mu > 0$; see also Remark 3.6. Note that by the independence of $X_{0, L}$ and $\xi _L$, the change of the probability spaces due to the use of the Skorokhod representation theorem (Lemma 2.19) does not affect the noise $\xi _L$ (and hence $Z_L$).

Let $\widetilde{X}_L = \widetilde{Y}_L + Z_L$ be the solution to (3.42) with the new initial data $\widetilde{X}_{0, L}$, where $\widetilde{Y}_L$ satisfies (3.45) with the initial data $X_{0, L}$ replaced by $\widetilde{X}_{0, L}$. By the invariance of $\rho _{L}$ under (3.42), we have ${{\,\textrm{Law}\,}}(\widetilde{X}_{L}(1))= \rho _{L}$. Thus, it suffices to show that

(i) $\Xi _0( \widetilde{X}_{L}(1) )$ converges in law to $\Xi _0( \widetilde{X}_\infty (1) )$ as $L \rightarrow \infty$ (with $L \in {\mathcal {A}}$), where $\widetilde{X}_\infty$ is the solution to (3.1) with the initial data $\widetilde{X}_{0, \infty }$ satisfying ${{\,\textrm{Law}\,}}(\widetilde{X}_{0, \infty }) = \rho _{\infty }$, and $\Xi _0(\phi )$ is the enhanced data set defined in (4.12), and
(ii) ${{\,\textrm{Law}\,}}(\widetilde{X}_\infty (1)) = \rho _\infty$. See Remark 4.7 below.

Before proceeding further, we recall the decomposition $\widetilde{X}_\infty = \widetilde{Y}_\infty + Z_\infty$, where $\widetilde{Y}_\infty$ is the solution to (3.4) with the initial data $\widetilde{X}_{0, \infty }$ and $Z_\infty$ is the solution to (3.2). For simplicity of notation, we denote $\widetilde{X}_L$, $\widetilde{Y}_L$, etc. by $X_L$, $Y_L$, etc. in the remaining part of the proof.

Given $R > 0$, let $B_R \subset {\mathbb {R}}^2$ denotes the ball of radius *R* centered at the origin. In view of the tightness of $\{ \nu _L\}_{L \in {\mathcal {A}}}$ (Proposition 4.4), it suffices to show that, given any $t \in {\mathbb {R}}_+$ and $R > 0$, $: \!( {\mathcal {S}}(t)X_{L}(1))^\ell \!:$ converges in probability to $: \!( {\mathcal {S}}(t)X_\infty (1))^\ell \!:$ in $W^{-\varepsilon , p}(B_R)$ as $L \rightarrow \infty$ (with $L \in {\mathcal {A}}$). Then, for each fixed $t \in {\mathbb {R}}_+$, this allows us to identify $: \!( {\mathcal {S}}(t)X_\infty (1))^\ell \!:$ with the limit in law of $: \!( {\mathcal {S}}(t)X_{L}(1))^\ell \!:$ as a ${\mathcal {D}}'({\mathbb {R}}^2)$-valued random variable by testing against a test function $\varphi \in {\mathcal {D}}({\mathbb {R}}^2) = C^\infty _c({\mathbb {R}}^2)$ with $\mathop {\textrm{supp}}\limits \varphi \subset B_R$. From this observation and Proposition 4.4 with the uniqueness of a limit, we then conclude that the entire sequence $\{\Xi _0(X_L(1))\}_{L\in {\mathcal {A}}}$ converges in law to $\Xi _0(X_\infty (1))$ as $L \rightarrow \infty$ (with $L \in {\mathcal {A}}$).

#### Remark 4.7

Once we prove (i) in the sense explained above, the claim (ii) above follows immediately. On the one hand, as probability measures on $B^{-\varepsilon , \mu }_{p_0, p_0}({\mathbb {R}}^2)$, $\rho _L = {{\,\textrm{Law}\,}}(X_L(1))$ converges weakly to $\rho _\infty$ as $L \rightarrow \infty$ (with $L \in {\mathcal {A}})$. On the other hand, we will show that, given $t \in {\mathbb {R}}_+$, ${\mathcal {S}}(t)X_{L}(1)$ converges in law to ${\mathcal {S}}(t)X_\infty (1)$ as $L \rightarrow \infty$ (with $L \in {\mathcal {A}})$. With $t = 0$, this implies that, as probability measures on ${\mathcal {D}}'({\mathbb {R}}^2)$, $\rho _L = {{\,\textrm{Law}\,}}(X_L(1))$ converges weakly to ${{\,\textrm{Law}\,}}(X_\infty (1))$. Therefore, from the uniqueness of a limit, we conclude that ${{\,\textrm{Law}\,}}( X_\infty (1)) = \rho _\infty$.

Note that a similar argument shows that ${{\,\textrm{Law}\,}}( X_\infty (t)) = \rho _\infty$ for any $t > 0$, thus yielding invariance of the limiting $\Phi ^{k+1}_2$-measure $\rho _\infty$ on ${\mathbb {R}}^2$ under the dynamics of the parabolic $\Phi ^{k+1}_2$-model (3.1) claimed in Remark 1.5.

Fix $t \in {\mathbb {R}}_+$ and $R> 0$ in the remaining part of the proof. From Lemma 2.8 (ii) (see also Remark 2.9) with (4.38) and (4.39), we have

4.62 $$\begin{aligned} \Vert&:\! ({\mathcal {S}}(t) X_L(1))^\ell \!: \, - :\!({\mathcal {S}}(t) X_{L'}(1))^\ell \!: \, \Vert _{W^{-\varepsilon , p}(B_R)} \nonumber \\&\lesssim \sum _{m=0}^\ell \left( {\begin{array}{c}\ell \\ m\end{array}}\right) \Big \{\Vert :\!({\mathcal {S}}(t)Z_{L}(1))^m\!: - :\!({\mathcal {S}}(t)Z_{L'}(1))^m\!: \Vert _{W^{-\varepsilon , p_1}(B_R)}\nonumber \\&\qquad \qquad \times \Vert ({\mathcal {S}}(t)Y_{L}(1) )^{\ell -m}\Vert _{W^{\varepsilon , p_2}(B_R)} \\&\qquad + \Vert :\!({\mathcal {S}}(t)Z_{L'}(1))^m\!: \Vert _{W^{-\varepsilon , p_1}(B_R)}\nonumber \\&\qquad \qquad \times \Vert ({\mathcal {S}}(t)Y_{L}(1) )^{\ell -m} - ({\mathcal {S}}(t)Y_{L'}(1) )^{\ell -m}\Vert _{W^{\varepsilon , p_2}(B_R)} \Big \}\nonumber \end{aligned}$$

for any $L, L' \in {\mathcal {A}}$, where $1< p_1, p_2 < \infty$ with $p_1 \gg 1$, satisfying $\frac{1}{p} \le \frac{1}{p_1} + \frac{1}{p_2} \le \frac{1}{p} + \frac{\varepsilon }{2}$. In the following, we separately study convergence of $({\mathcal {S}}(t)Y_{L}(1) )^{\ell -m}$ and $:\!({\mathcal {S}}(t)Z_{L'}(1))^m\!:$ .

$\bullet$ **Part 1:** Let us first study the terms involving $Y_L$ and $Y_{L'}$. Proceeding as in (4.58) with the fractional Leibniz rule (Lemma 2.8 (i)), Sobolev’s inequality, and the boundedness of ${\mathcal {S}}(t)$ on $H^s({\mathbb {R}}^2)$, we have

4.63 $$\begin{aligned} \begin{aligned} \Vert ({\mathcal {S}}(t)Y_L(1) )^{\ell - m}\Vert _{L^\infty _t W_x^{\varepsilon , p_2}(B_R)}&\lesssim \Vert {\mathcal {S}}(t)Y_L(1) \Vert ^{\ell - m}_{L^\infty _t W_x^{\varepsilon , (\ell - m)p_2 }(B_R)}\\&\lesssim \Vert {\mathcal {S}}(t)Y_L(1) \Vert ^{\ell - m}_{L^\infty _t H_x^{1 - \frac{2}{(\ell - m)p_2} + \varepsilon }(B_R)}\\&\lesssim \Vert Y_L(1) \Vert ^{\ell - m} _{H^{1 - \frac{2}{(\ell - m)p_2} + \varepsilon }(B_R)}. \end{aligned} \end{aligned}$$

Then, by Hölder’s inequality and (2.26), we have

4.64 $$\begin{aligned} \begin{aligned} \Vert ({\mathcal {S}}(t)Y_L(1) )^{\ell - m}\Vert _{L^\infty _t W_x^{\varepsilon , p_2}(B_R)}&\le C(R) \Vert Y_L(1) \Vert ^{\ell - m} _{B^{1 - \frac{2}{(\ell - m)p_2} + 2\varepsilon }_{p_3, \infty }(B_R)} \end{aligned} \end{aligned}$$

for any $p_3 \ge 2$. Proposition 3.3 with Theorem 5.1 in [60],^34^ controlling the right-hand side of (3.28), shows that, given any finite $q\ge 1$, the *q*th moment of the right-hand side of (4.64) is bounded, uniformly in $L \ge 1$.

A slight modification of (4.63) and (4.64) yields

4.65 $$\begin{aligned} \begin{aligned} \Vert&( {\mathcal {S}}(t)Y_L(1) )^{\ell - m} - ({\mathcal {S}}(t)Y_{L'} (1) )^{\ell - m}\Vert _{L^\infty _t W_x^{\varepsilon , p_2}(B_R)} \\&\le C(R) \Big ( \Vert Y_L(1) \Vert ^{\ell - m- 1} _{B^{1 - \frac{2}{(\ell - m)p_2} + 2\varepsilon }_{p_3, \infty }(B_R)} + \Vert Y_{L'}(1) \Vert ^{\ell - m- 1} _{B^{1 - \frac{2}{(\ell - m)p_2} + 2\varepsilon }_{p_3, \infty }(B_R)} \Big )\\&\quad \times \Vert Y_L(1) - Y_{L'}(1) \Vert _{B^{1 - \frac{2}{(\ell - m)p_2} + 2\varepsilon }_{p_3, \infty }(B_R)} \end{aligned} \end{aligned}$$

for any $$L, L' \ge 1$$.

Given $L \ge 1$, let ${\textbf{Z}}_L$ and ${\textbf{Y}}_L$ be the solutions to

$$\begin{aligned} {\left\{ \begin{array}{ll} \partial _t{\textbf{Z}}_L + (1-\Delta ) {\textbf{Z}}_L = \sqrt{2}\xi _L \\ {\textbf{Z}}_L|_{t = 0} = X_{0, L} \end{array}\right. } \end{aligned}$$

and

$$\begin{aligned} {\left\{ \begin{array}{ll} \partial _t{\textbf{Y}}_L + (1-\Delta ) {\textbf{Y}}_L + \sum _{\ell =0}^k {k\atopwithdelims ()\ell } :\!{\textbf{Z}}_L^\ell \!: \,{\textbf{Y}}_L^{k-\ell } = 0\\ {\textbf{Y}}_L|_{t=0} = 0, \end{array}\right. } \end{aligned}$$

respectively. Note that we have $X_L = Y_L + Z_L = {\textbf{Y}}_L + {\textbf{Z}}_L$ and that

4.66 $$\begin{aligned} Y_L(1) - Y_{L'}(1) = {\textbf{Y}}_L(1) - {\textbf{Y}}_{L'}(1) - P(1) (X_{0, L} - X_{0, L'}). \end{aligned}$$

By the Schauder estimate (Proposition 5 in [60]^35^) and the almost sure convergence of $X_{0, L}$ in $B^{ -\varepsilon , \mu }_{p_3, \infty }({\mathbb {R}}^2)$, we have

4.67 $$\begin{aligned} \Vert P(1) (X_{0, L} - X_{0, L'})\Vert _{B^{1 - \frac{2}{(\ell - m)p_2} + 2\varepsilon }_{p_3, \infty }(B_R)} \longrightarrow 0, \end{aligned}$$

almost surely, as $L, L' \rightarrow \infty$ (with $L, L' \in {\mathcal {A}}$).

Next, we study the difference ${\textbf{Y}}_L(1) - {\textbf{Y}}_{L'}(1)$ in (4.66). Write $:\!{\textbf{Z}}_L^\ell \!:$ as

$$\begin{aligned} :\!{\textbf{Z}}_L^\ell \!:\, = \sum _{m=0}^\ell {\ell \atopwithdelims ()m} \big (P(t)X_{0, L}\big )^{\ell - m} :\!Z_L^m\!:. \end{aligned}$$

Then, it follows from the almost sure convergence of $P(t)X_{0, L}$ and Theorem 5.1 in [60]^36^ on convergence in probability of $:\!Z_L^m\!:$ together with Lemma 2.11 (ii) that $:\!{\textbf{Z}}_L^\ell \!:$ converges in probability to a limit in $C([0, 1]; B^{-\varepsilon , \mu }_{p_4, \infty }({\mathbb {R}}^2))$ for some $p_4 \gg 1$, as $L \rightarrow \infty$ (with $L \in {\mathcal {A}}$). Now, recall from Theorem 8.1 and Theorem 9.2 in [60]^37^ that the solution map for the following (deterministic) equation:

$$\begin{aligned} {\left\{ \begin{array}{ll} \partial _t{\textbf{Y}}+ (1-\Delta ) {\textbf{Y}}+ \sum _{\ell =0}^k {k\atopwithdelims ()\ell } {\textbf{Z}}^{(\ell )} {\textbf{Y}}^{k-\ell } = 0\\ {\textbf{Y}}|_{t=0} = 0, \end{array}\right. } \end{aligned}$$

sending $\{ {\textbf{Z}}^{(\ell )}\}_{\ell = 0}^k$ (with ${\textbf{Z}}^{(0)} = 1$) to a solution ${\textbf{Y}}$ is continuous from $\big ( C([0, 1]; B^{-\varepsilon , \mu }_{p_4, \infty }({\mathbb {R}}^2))\big )^{\otimes k}$ to $C([0, 1]; B^{2-\varepsilon , \mu }_{p_5, \infty }({\mathbb {R}}^2))$ for some $p_5 \gg 1$. In view of the aforementioned convergence in probability of $:\!{\textbf{Z}}_L^\ell \!:$, $\ell = 1, \dots , k$, the continuous mapping theorem then yields that

4.68 $$\begin{aligned} \Vert {\textbf{Y}}_L(1) - {\textbf{Y}}_{L'}(1)\Vert _{B^{1 - \frac{2}{(\ell - m)p_2} + 2\varepsilon }_{p_3, \infty }(B_R)} \longrightarrow 0 \end{aligned}$$

in probability, as $L, L' \rightarrow \infty$ (with $L, L' \in {\mathcal {A}}$).

Therefore, putting (4.66), (4.67), and (4.68) together with the aforementioned uniform (in *L*) boundedness of the $B^{1 - \frac{2}{(\ell - m)p_2} + 2\varepsilon }_{p_3, \infty }(B_R)$-norm of $Y_L(1)$ (see the discussion after (4.64)), we conclude from (4.65) that $( {\mathcal {S}}(t)Y_L(1) )^{\ell - m}$ converges in probability to a unique limit in $W^{\varepsilon , p_2}(B_R)$ as $L \rightarrow \infty$ (with $L \in {\mathcal {A}})$. Moreover, by repeating the argument with $L' = \infty$, we see that the limit is given by $({\mathcal {S}}(t)Y_{\infty } (1) )^{\ell - m}$, where $Y_\infty$ is the solution to (3.4) with the Gibbsian initial data $X_{0, \infty }$.

$\bullet$ **Part 2:** In view of (4.62) and the discussion above, it suffices to show that, given any $R > 0$, there exists $r \ge 1$ such that

4.69 $$\begin{aligned} : \!( {\mathcal {S}}(t)Z_{L}(1))^m \!: \ \ \text {converges to} \ \ : \!( {\mathcal {S}}(t)Z_\infty (1))^m \!: \end{aligned}$$

in $L^r(\Omega ; W^{-\varepsilon , p_1}(B_R))$ as $L \rightarrow \infty$, $m = 1, \dots , k$. We point out that in showing (4.69), there is no need to restrict *L* in ${\mathcal {A}}$. Indeed, once we prove (4.69), it follows from (4.62), Part 1, and (4.69) (which in particular implies convergence in probability) that $:\! ({\mathcal {S}}(t) X_L(1))^\ell \!:$ converges in law to $:\!({\mathcal {S}}(t) X_{\infty }(1))^\ell \!:$ in ${W^{-\varepsilon , p}(B_R)}$ as $L \rightarrow \infty$ (with $L \in {\mathcal {A}}$) for any $t \in {\mathbb {R}}_+$, $R > 0$, and each $\ell = 1, \dots , k$.

Hence, it remains to prove (4.69). Fix $t \in {\mathbb {R}}_+$ and $R> 0$. Given $L, L' \ge 1$, $x, y \in {\mathbb {R}}^2$, define $K_{L, L'}(x, y;t)$ by

4.70 $$\begin{aligned} \begin{aligned} K_{L, L'}(x, y;t)&= {\mathbb {E}}\big [ \big ({\mathcal {S}}(t)Z_L(1)\big )(x) \big ({\mathcal {S}}(t)Z_{L'}(1)\big )(y) \big ] \\&= K_{L, L'}(y, x;t). \end{aligned} \end{aligned}$$

Then, from Lemma 2.13, we have

4.71 $$\begin{aligned} {\mathbb {E}}\big [ :\!( {\mathcal {S}}(t)Z_L(1) )^m (x) \!: \, :\!( {\mathcal {S}}(t)Z_{L'}(1) )^m(y)\!: \big ] = m ! \big \{K_{L, L'}(x, y;t)\big \}^m . \end{aligned}$$

Given a test function $\varphi \in {\mathcal {D}}({\mathbb {R}}^2)$ supported on a ball *B* of radius 1 with $B \subset B_{2R}$, we set

4.72 $$\begin{aligned} K^{m,\varphi }_{L, L'}(t)&= {\mathbb {E}}\Big [ | \langle :\!( {\mathcal {S}}(t)Z_L(1) )^m\! : - :\!( {\mathcal {S}}(t)Z_{L'}(1) )^m \!: \, , \varphi \rangle |^2 \Big ], \end{aligned}$$

where $\langle \cdot , \cdot \rangle$ denotes the ${\mathcal {D}}'$-${\mathcal {D}}$ duality pairing. Then, from (4.71) and Hölder’s inequality, we have

4.73 $$\begin{aligned} \begin{aligned} K^{m,\varphi }_{L, L'}(t)&= m ! \iint _{(B_{2R})^2} \Big ( \big \{K_{L, L}(x, y;t)\big \}^m - 2\big \{K_{L, L'}(x, y;t)\big \}^m \\&\qquad \quad + \big \{K_{L', L'}(x, y;t)\big \}^m \Big ) \varphi (x)\varphi (y) dxdy \\&\lesssim \big \Vert \big \{K_{L, L}(x, y;t)\big \}^m - 2\big \{K_{L, L'}(x, y;t)\big \}^m\\&\qquad \quad + \big \{K_{L', L'}(x, y;t)\big \}^m \big \Vert _{L^{q}_{x,y}(B_{2R})} \Vert \varphi \Vert _{L^{q'}}^2 \end{aligned} \end{aligned}$$

for any $1 \le q \le \infty$ with $\frac{1}{q} + \frac{1}{q'} = 1$.

Now, set $K_{\infty , \infty }(x, y;t) = \lim _{L, L'\rightarrow \infty } K_{L, L'}(x, y;t)$; see (4.95) below for the precise definition. We then claim that there exists $c > 0$ such that

4.74 $$\begin{aligned} \begin{aligned} |K_{L, L'}(x, y;t) | + |K_{\infty , \infty }(x, y;t) |&\lesssim 1+ (- \log |x-y| )_+ , \\ | K_{L, L'}(x, y;t)- K_{\infty , \infty } (x, y;t)|&\lesssim e^{-cL} + e^{-cL'} \end{aligned} \end{aligned}$$

for any $L, L' \gg R+t$ and $x, y \in B_{2R}$, where $a_+ = \max (a, 0)$.

For now, we assume (4.74) and prove (4.69). By the triangle inequality and (4.74), we have

4.75 $$\begin{aligned} \begin{aligned} \big |&\big \{K_{L, L}(x, y;t)\big \}^m - 2\big \{K_{L, L'}(x, y;t)\big \}^m + \big \{K_{L', L'}(x, y;t)\big \}^m \big | \\&\le \big | \big \{K_{L, L}(x, y;t)\big \}^m - \big \{K_{\infty ,\infty }(x, y;t)\big \}^m \big |\\&\quad + 2 \big | \big \{K_{L, L'}(x, y;t)\big \}^m - \big \{K_{\infty ,\infty }(x, y;t)\big \}^m \big | \\&\quad + \big | \big \{K_{L', L'}(x, y;t)\big \}^m - \big \{K_{\infty ,\infty }(x, y;t)\big \}^m \big | \\&\lesssim \big ( 1 + (-\log |x-y| )_+ \big )^{m -1} \big ( e^{-c L} + e^{-c L'} \big ). \end{aligned} \end{aligned}$$

Hence, by choosing $q = q(\varepsilon ) \gg 1$ and applying Lemma 2.14 with (4.72), (4.73), and (4.75), we obtain

4.76 $$\begin{aligned} \begin{aligned} {\mathbb {E}}&\Big [ \Vert :\!( {\mathcal {S}}(t)Z_L(1) )^m \!: - :\!( {\mathcal {S}}(t)Z_{L'}(1) )^m \! : \Vert _{W^{-\varepsilon , p_1}(B_R)}^r \Big ] \\&\le C(R, r) \sup _{ \begin{array}{c} \Vert \varphi \Vert _{L^{q'}} \le 1 \\ \mathop {\textrm{supp}}\limits (\varphi )\subset B \subset B_{2R} \end{array}} \big (K^{m, \varphi }_{L, L'}(t)\big )^\frac{r}{2}\\&\lesssim C(R, r) \big ( e^{-c L} + e^{-c L'} \big ), \end{aligned} \end{aligned}$$

which tends to zero as $L, L'\rightarrow \infty$. This shows that $: \!( {\mathcal {S}}(t)Z_{L}(1))^m \!:$ converges to some limit in $L^r(\Omega ; W^{-\varepsilon , p_1}(B_R))$ as $L \rightarrow \infty$. By repeating an analogous argument with $L' = \infty$, we obtain (4.69). See Remark 4.8 below.

The remaining part of the proof is devoted to proving the claim (4.74); see Remark 4.8 below for the case $L' = \infty$. Let $\varphi \in L^2({\mathbb {R}}^2)$. Then, from (4.43) with (4.4) and (4.5), we have

$$\begin{aligned} \langle {\mathcal {S}}(t) Z_L(1), \varphi \rangle = \sqrt{2} \xi \Big ( {\textbf{1}}_{[0, 1]}(t'){\textbf{1}}_{[-\frac{L}{2}, \frac{L}{2})^2} P(1- t') {\mathcal {S}}(t) \varphi ^\text {per}_L \Big ), \end{aligned}$$

where $\varphi ^\text {per}_L$ is the *L*-periodized version of $\varphi$ defined by

4.77 $$\begin{aligned} \varphi ^\text {per}_L (x) = \sum _{n \in {\mathbb {Z}}^2} \varphi (x + n L). \end{aligned}$$

Given $x \in {\mathbb {R}}^2$, we then have

$$\begin{aligned} \big ({\mathcal {S}}(t) Z_L(1)\big )(x)&= \langle {\mathcal {S}}(t) Z_L(1), \delta _x \rangle \\&= \sqrt{2} \xi \Big ( {\textbf{1}}_{[0, 1]}(t'){\textbf{1}}_{[-\frac{L}{2}, \frac{L}{2})^2} P(1- t') {\mathcal {S}}(t) (\delta _x)^\text {per}_L \Big ), \end{aligned}$$

where $\delta _x$ denotes the Dirac delta function at $x \in {\mathbb {R}}^2$, and hence we can rewrite (4.70) as

4.78 $$\begin{aligned} \begin{aligned} K_{L, L'}(x, y;t)&=2{\mathbb {E}}\bigg [ \xi \Big ( {\textbf{1}}_{[0, 1]}(t'){\textbf{1}}_{[-\frac{L}{2}, \frac{L}{2})^2} P(1- t') {\mathcal {S}}(t) (\delta _x)^\text {per}_L \Big )\\&\qquad \quad \times \xi \Big ( {\textbf{1}}_{[0, 1]}(t') {\textbf{1}}_{[-\frac{L'}{2}, \frac{L'}{2})^2} P(1- t') {\mathcal {S}}(t) (\delta _y)^\text {per}_{L'} \Big ) \bigg ]\\&=2 \int _{{\mathbb {R}}^2}\int _0^1 \Big ( {\textbf{1}}_{[-\frac{L}{2}, \frac{L}{2})^2} P(t') {\mathcal {S}}(t) (\delta _x)^\text {per}_L \Big )(z)\\&\qquad \quad \times \Big ( {\textbf{1}}_{[-\frac{L'}{2}, \frac{L'}{2})^2} P(t') {\mathcal {S}}(t) (\delta _y)^\text {per}_{L'} \Big )(z)dt' dz . \end{aligned} \end{aligned}$$

In the following, we assume $x, y \in B_{2R}$. By the finite speed of propagation, we have

4.79 $$\begin{aligned} \mathop {\textrm{supp}}\limits {\mathcal {S}}(t) \delta _{x+ nL} \subset x + nL + B_t. \end{aligned}$$

Let $\{\psi _j^L\}_{j \in {\mathbb {Z}}_{\ge 0}}$ be a smooth partition of unity on ${\mathbb {R}}^2$ such that $\psi _0^L = 1$ on $B_{\frac{L}{8}}$, $\mathop {\textrm{supp}}\limits \psi _0^L \subset B_{\frac{L}{4}}$, and

4.80 $$\begin{aligned} \mathop {\textrm{supp}}\limits \psi _j^L \subset \big \{ \max \big ((j - 1), \tfrac{1}{8}\big )L \le |z| \le (j + 1) L\big \}. \end{aligned}$$

In particular, for $j = 0$, we set $\psi _0^L(z) = \psi _0\big (\frac{z}{L}\big )$ for some $\psi _0 \in C_c^\infty ({\mathbb {R}}^2)$. Here, we choose $\{\psi _j^L\}_{j \in {\mathbb {Z}}_{\ge 0}}$ such that their first and second derivatives are uniformly bounded. Then, we have

$$\begin{aligned} {\textbf{1}}_{[-\frac{L}{2}, \frac{L}{2})^2} P(t') {\mathcal {S}}(t) (\delta _x)^\text {per}_{L} = e^{-t'} \sum _{j = 0}^\infty {\textbf{1}}_{[-\frac{L}{2}, \frac{L}{2})^2} \big \{ (\psi _j^L p_{t'})* ({\mathcal {S}}(t) (\delta _x)^\text {per}_{L})\big \}, \end{aligned}$$

where $p_{t'}$ is as in (2.6). Under the assumption $L \gg R+t$ and $x \in B_{2R}$, it follows from (4.77) and (4.79) that

4.81 $$\begin{aligned} \begin{aligned} e^{-t'}{\textbf{1}}_{[-\frac{L}{2}, \frac{L}{2})^2} (z) \cdot \big \{ (\psi _0^L p_{t'})* ({\mathcal {S}}(t) (\delta _x)^\text {per}_{L})\big \}(z)&= e^{-t'} (\psi _0^L p_{t'})* ({\mathcal {S}}(t) \delta _x)(z)\\&= e^{-t'} {\mathcal {S}}(t)(\psi _0^L p_{t'})(z-x). \end{aligned} \end{aligned}$$

By the boundedness of ${\mathcal {S}}(t)$ on $H^s({\mathbb {R}}^2)$ and $\psi _0^L(z) = \psi _0\big (\frac{z}{L}\big )$ for some $\psi _0 \in C_c^\infty ({\mathbb {R}}^2)$, we have

4.82 $$\begin{aligned} \begin{aligned} \Vert (4.81)\Vert _{H^{-1-\varepsilon }_z}&\lesssim e^{-t'} \Vert \psi _0^L p_{t'}\Vert _{H^{-1-\varepsilon }}\\&\lesssim e^{-t'}\bigg \Vert \int _{\eta = \eta _1 + \eta _2} L^2 \widehat{\psi }_0(L\eta _1) e^{-4\pi ^2 t' |\eta _2|^2} d\eta _1\bigg \Vert _{L^\infty _\eta }\\&\lesssim e^{-t'}\int _{{\mathbb {R}}^2} \frac{L^2}{\langle L\eta _1 \rangle ^{100}} d\eta _1 \lesssim e^{-t'}, \end{aligned} \end{aligned}$$

uniformly in $x \in B_{2R}$, $t \ge 0$, and $t' \in [0, 1]$ with $R + t \ll L$.

For $j \in {\mathbb {N}}$, it follows from (4.77) and (4.80) with $L \gg R+t$ and $x \in B_{2R}$ that

4.83 $$\begin{aligned} \begin{aligned}&e^{-t'}{\textbf{1}}_{[-\frac{L}{2}, \frac{L}{2})^2} (z) \cdot \big \{ (\psi _j^L p_{t'})* ({\mathcal {S}}(t) (\delta _x)^\text {per}_{L})\big \}(z) \\&\quad = e^{-t'}{\textbf{1}}_{[-\frac{L}{2}, \frac{L}{2})^2} (z) \sum _{\begin{array}{c} n \in {\mathbb {Z}}^2\\ |n| = j+O(1) \end{array}} \bigg \{ (\psi _j^L p_{t'})* \Big ({\mathcal {S}}(t) \delta _{x+n L}\Big )\bigg \}(z) \\&\quad = e^{-t'}{\textbf{1}}_{[-\frac{L}{2}, \frac{L}{2})^2} (z) \bigg \{ \sum _{\begin{array}{c} n \in {\mathbb {Z}}^2\setminus \{0\}\\ |n| = j+O(1) \end{array}} {\mathcal {S}}(t)( \psi _j^L p_{t'})(z-x- nL)\\&\qquad \quad + {\textbf{1}}_{\{|z|\ge \frac{L}{16}\}} {\mathcal {S}}(t)( \psi _j^L p_{t'})(z-x)\bigg \}, \end{aligned} \end{aligned}$$

where the second term of the last factor is relevant only for $j = 1$. Note that, for $|z|\ge \frac{L}{16}$ and $x \in B_{2R}$ with $L \gg R+t$, we have $|z-x|\ge \frac{L}{32}$. From the definition of $\psi _j^L$ and (2.6), we then have

$$\begin{aligned} \Vert (4.83) \Vert _{L^2_z}&\lesssim j e^{-t'} \Vert \psi _j^L p_{t'} \Vert _{L^2_z(|z| \ge \frac{L}{32})}\\&\lesssim {\left\{ \begin{array}{ll} \exp \big (- c \frac{j^2 L^2}{t'} - t' \big ), & \text {for }0 < t'\ll jL, \\ \exp (- cjL), &  \text {for }t'\sim j L, \\ \exp (-ct'), &  \text {for }t' \gg j L. \end{array}\right. } \end{aligned}$$

Thus, we obtain

4.84 $$\begin{aligned} \Vert (4.83) \Vert _{L^2_z} \lesssim e^{-cjL}, \end{aligned}$$

uniformly in $x \in B_{2R}$, $t \ge 0$, and $t' \in [0, 1]$ with $R + t \ll L$.

Let $\chi _L \in C^\infty _c({\mathbb {R}}^2; [0, 1])$ be a smooth cutoff function such that $\chi _L \equiv 1$ on $\big [-\frac{L}{2}, \frac{L}{2}\big )^2$ and $\mathop {\textrm{supp}}\limits \chi _L \subset \big [-\frac{(1+ \gamma )L}{2}, \frac{(1+\gamma )L}{2}\big )^2$ for some small $\gamma > 0$. We assume that the first and second derivatives of $\chi _L$ are bounded uniformly in $L\gg 1$. Define $F(z) = F_{j, L, x, t, t'}(z)$by

$$\begin{aligned} F(z) = F_{j, L, x, t, t'}(z) = e^{-t'}\chi (z)\cdot \big \{ (\psi _j^L p_{t'})* ({\mathcal {S}}(t) (\delta _x)^\text {per}_{L})\big \}(z). \end{aligned}$$

Then, a similar computation with the uniform boundedness of the first and second derivatives of $\psi _j^L$ shows that

4.85 $$\begin{aligned} \Vert F_{j, L, x, t, t'} \Vert _{H^2_z} \lesssim \Vert F_{j, L, x, t, t'} \Vert _{L^2_z} + \Vert \Delta _z F_{j, L, x, t, t'} \Vert _{L^2_z} \lesssim e^{-cjL}, \end{aligned}$$

uniformly in $x \in B_{2R}$, $t \ge 0$, and $t' \in [0, 1]$ with $R + t \ll L$.

In view of the discussion above (in particular, see (4.81)), we write $K_{L, L'}(x,y; t)$ in (4.78) as

4.86 $$\begin{aligned} K_{L, L'}(x,y; t) = K_{L, L'}^{(0)}(x,y; t) + K_{L, L'}^{(1)}(x,y; t), \end{aligned}$$

where $$ K_{L, L'}^{(0)}(x,y; t)$$ is defined by

4.87 $$\begin{aligned} \begin{aligned} K_{L, L'}^{(0)}(x,y; t)&=2 \int _{{\mathbb {R}}^2}\int _0^1 \Big ( e^{-t'} (\psi _0^L p_{t'})* ({\mathcal {S}}(t) \delta _x) \Big )(z)\\&\qquad \quad \times \Big ( e^{-t'} (\psi _0^{L'} p_{t'})* ( {\mathcal {S}}(t) \delta _y) \Big )(z)dt' dz \end{aligned} \end{aligned}$$

and $K_{L, L'}^{(1)}( x,y; t) = K_{L, L'}(x,y; t) - K_{L, L'}^{(0)}(x,y; t)$. Then, from (4.78) and (4.87), we have

4.88 $$\begin{aligned} K_{L, L'}^{(1)}(x,y; t)&=2 \sum _{j = 1}^\infty \sum _{j' = 1}^\infty \int _{{\mathbb {R}}^2}\int _0^1 \Big ( {\textbf{1}}_{[-\frac{L}{2}, \frac{L}{2})^2} \, e^{-t'} (\psi _j^L p_{t'})* ({\mathcal {S}}(t) (\delta _x)^\text {per}_L) \Big )(z)\nonumber \\&\qquad \qquad \quad \times \Big ( {\textbf{1}}_{[-\frac{L'}{2}, \frac{L'}{2})^2} \, e^{-t'} (\psi _{j'}^{L'} p_{t'})* ( {\mathcal {S}}(t) (\delta _y)^\text {per}_{L'}) \Big )(z)dt' dz \nonumber \\&\qquad + 2 \sum _{j = 1}^\infty \int _{{\mathbb {R}}^2}\int _0^1 \Big ( {\textbf{1}}_{[-\frac{L}{2}, \frac{L}{2})^2} e^{-t'} (\psi _j^L p_{t'})* ({\mathcal {S}}(t) (\delta _x)^\text {per}_L) \Big )(z)\nonumber \\&\qquad \qquad \quad \times \Big ( e^{-t'} (\psi _{0}^{L'} p_{t'})* ( {\mathcal {S}}(t) \delta _y) \Big )(z)dt' dz \nonumber \\&\qquad + 2 \sum _{j' = 1}^\infty \int _{{\mathbb {R}}^2}\int _0^1 \Big ( e^{-t'} (\psi _0^{L} p_{t'})* ({\mathcal {S}}(t) \delta _x) \Big )(z)\nonumber \\&\qquad \qquad \quad \times \Big ( {\textbf{1}}_{[-\frac{L'}{2}, \frac{L'}{2})^2} e^{-t'} (\psi _{j'}^{L'} p_{t'})* ( {\mathcal {S}}(t) (\delta _y)^\text {per}_{L'}) \Big )(z)dt' dz \nonumber \\&= : \hspace{0.5mm}\text {I}\hspace{0.5mm} (x,y; t)+ \text {II}(x,y; t) + \text {III}(x,y; t). \end{aligned}$$

Note that, in view of (4.81), we dropped the cutoff function ${\textbf{1}}_{[-\frac{L'}{2}, \frac{L'}{2})^2}$ (and ${\textbf{1}}_{[-\frac{L}{2}, \frac{L}{2})^2}$) in $\text {II}$ (and in $\text {III}$, respectively). By Cauchy-Schwarz’s inequality with (4.83) and (4.84), we have

4.89 $$\begin{aligned} |\hspace{0.5mm}\text {I}\hspace{0.5mm}(x,y; t)|&\lesssim \sum _{j = 1}^\infty \sum _{j' = 1}^\infty e^{- cj L} e^{- cj'L'} \lesssim e^{- c( L+L')}. \end{aligned}$$

Next, we estimate II and III in (4.88). Without loss of generality, assume $L \ge L'$. As for $\text {II}$, we first insert $\chi _L$ and then drop the cutoff function ${\textbf{1}}_{[-\frac{L}{2}, \frac{L}{2})^2}$ in view of (4.81). Thus, from (4.82) and (4.85), we have

4.90 $$\begin{aligned} \begin{aligned} |\text {II} (x, y; t)|&= 2 \bigg | \sum _{j = 1}^\infty \int _{{\mathbb {R}}^2}\int _0^1 \Big ( e^{-t'} \chi _L(z)(\psi _j^L p_{t'})* ({\mathcal {S}}(t) (\delta _x)^\text {per}_L) \Big )(z)\\&\qquad \qquad \quad \times \Big ( e^{-t'} (\psi _{0}^{L'} p_{t'})* ( {\mathcal {S}}(t) \delta _y) \Big )(z)dt' dz \bigg |\\&\lesssim \sum _{j = 1}^\infty \int _0^1 \big \Vert e^{-t'} \chi _L (\psi _j^L p_{t'})* ({\mathcal {S}}(t) (\delta _x)^\text {per}_L) \big \Vert _{H^2_z}\\&\qquad \qquad \quad \times \Vert e^{-t'} (\psi _{0}^{L'} p_{t'})* ( {\mathcal {S}}(t) \delta _y) \Vert _{H^{-1-\varepsilon }_z}dt' \\&\lesssim e^{-cL}. \end{aligned} \end{aligned}$$

As for the term III, we can not drop the cutoff function ${\textbf{1}}_{[-\frac{L'}{2}, \frac{L'}{2})^2}$ under the current assumption $L \ge L'$, and thus we need to proceed with more care. We first note that by the mean value theorem, we have

4.91 $$\begin{aligned} |{\mathcal {F}}\big ( {\textbf{1}}_{[-\frac{L'}{2}, \frac{L'}{2})^2} \big )(\eta )| \lesssim \frac{(L')^2}{\langle \eta ^1 \rangle \langle \eta ^2 \rangle }, \end{aligned}$$

where $\eta = (\eta ^1, \eta ^2)$. Then, proceeding as in (4.82) with the triangle inequality $\langle \eta _1 \rangle ^\varepsilon \lesssim \langle \eta \rangle ^\varepsilon \langle \eta _2 \rangle ^\varepsilon \langle \eta _3 \rangle ^\varepsilon$, Young’s inequality, and (4.91), we have

4.92 $$\begin{aligned} \begin{aligned} \big \Vert&{\textbf{1}}_{[-\frac{L'}{2}, \frac{L'}{2})^2}(z) {\mathcal {S}}(t)(\psi _0^L p_{t'})(z-x)\big \Vert _{H^{-1-2\varepsilon }_z}\\&\lesssim \bigg \Vert \frac{1}{\langle \eta \rangle ^{1 + 2\varepsilon }} \int _{\eta = \eta _1 + \eta _2+ \eta _3} |{\mathcal {F}}\big ( {\textbf{1}}_{[-\frac{L'}{2}, \frac{L'}{2})^2} \big )(\eta _1)| L^2|\widehat{\psi }_0(L\eta _2)| e^{-4\pi ^2 t' |\eta _3|^2} d\eta _1d\eta _2 \bigg \Vert _{L^2_\eta }\\&\lesssim \bigg \Vert \frac{1}{\langle \eta \rangle ^{1 + \varepsilon }} \int _{\eta = \eta _1 + \eta _2+ \eta _3} \langle \eta _1 \rangle ^{-\varepsilon }|{\mathcal {F}}\big ( {\textbf{1}}_{[-\frac{L'}{2}, \frac{L'}{2})^2} \big )(\eta _1)|\\&\times \langle \eta _2 \rangle ^{\varepsilon } L^2|\widehat{\psi }_0(L\eta _2)| \langle \eta _3 \rangle ^{\varepsilon } e^{-4\pi ^2 t' |\eta _3|^2} d\eta _1d\eta _2 \bigg \Vert _{L^2_\eta }\\&\lesssim (L')^2 (t')^{-\frac{\varepsilon }{2}}. \end{aligned} \end{aligned}$$

Hence, from (4.85) and (4.92), we obtain

4.93 $$\begin{aligned} \begin{aligned} |\text {III}(x,y; t)|&= 2\bigg | \sum _{j' = 1}^\infty \int _{{\mathbb {R}}^2}\int _0^1 e^{-t'} {\textbf{1}}_{[-\frac{L'}{2}, \frac{L'}{2})^2}(z) {\mathcal {S}}(t)(\psi _0^L p_{t'})(z-x)\\&\qquad \qquad \quad \times \Big ( e^{-t'} \chi _{L'}(z) (\psi _{j'}^{L'} p_{t'})* ( {\mathcal {S}}(t) (\delta _y)^\text {per}_{L'}) \Big )(z)dt' dz\bigg | \\&\lesssim (L')^2 e^{-cL'} \int _0^1 (t')^{-\frac{\varepsilon }{2}} dt' \\&\lesssim e^{-c'L'}. \end{aligned} \end{aligned}$$

Therefore, from (4.88), (4.89), (4.90), and (4.93), we obtain

4.94 $$\begin{aligned} |K_{L, L'}^{(1)}(x,y; t)| \lesssim e^{-cL} + e^{-cL'}. \end{aligned}$$

Next, we study $$K^{(0)}_{L, L'}(x, y;t) $$ for $$L, L' \gg 1$$. Define $$K_{\infty , \infty }(x, y; t) $$ by

4.95 $$\begin{aligned} \begin{aligned} K_{\infty , \infty }(x,y; t)&=2 \int _{{\mathbb {R}}^2} \int _0^1 \big ( P(t') ({\mathcal {S}}(t) \delta _x \big )(z) \big (P(t') {\mathcal {S}}(t) \delta _y \big )(z)dt' dz \\&= \big \langle (1-\Delta )^{-1} (1- e^{-2(1-\Delta )}) {\mathcal {S}}(t) \delta _{x}, {\mathcal {S}}(t) \delta _{y} \big \rangle _{L^2_z}. \end{aligned} \end{aligned}$$

From (4.87) and (4.95), we have

4.96 $$\begin{aligned} \begin{aligned}&K_{\infty , \infty } (x,y; t) - K_{L, L'}^{(0)}(x,y; t)\\&\quad = 2 \int _{{\mathbb {R}}^2}\int _0^1 \Big ( e^{-t'} \big ((1- \psi _0^L) p_{t'}\big )* ({\mathcal {S}}(t) \delta _x) \Big )(z) \\&\qquad \qquad \qquad \times \Big ( P(t') {\mathcal {S}}(t) \delta _y \Big )(z)dt' dz \\&\quad \quad + 2 \int _{{\mathbb {R}}^2}\int _0^1 \Big ( e^{-t'} (\psi _0^L p_{t'})* ({\mathcal {S}}(t) \delta _x) \Big )(z) \\&\qquad \qquad \qquad \times \Big ( e^{-t'} \big ((1-\psi _0^{L'}) p_{t'}\big )* ( {\mathcal {S}}(t) \delta _y) \Big )(z)dt' dz \\&\quad = 2 \int _{{\mathbb {R}}^2}\int _0^1 \Big ( e^{-t'}{\mathcal {S}}(t) \big ((1- \psi _0^L) p_{t'}\big ) \Big )(z-x) \\&\qquad \qquad \qquad \times \Big ( e^{-t'} {\mathcal {S}}(t) p_{t'}\Big )(z-y)dt' dz \\&\quad \quad + 2 \int _{{\mathbb {R}}^2}\int _0^1 \Big ( e^{-t'} {\mathcal {S}}(t)(\psi _0^L p_t') \Big )(z-x) \\&\qquad \qquad \qquad \times \Big ( e^{-t'} {\mathcal {S}}(t) \big ((1-\psi _0^{L'}) p_{t'}\big ) \Big )(z-y)dt' dz \\&\quad =: \text {IV}(x, y; t) + \text {V}(x, y; t). \end{aligned} \end{aligned}$$

We only consider the first term IV on the right-hand side of (4.96) since the term $\text {V}$ can be treated in a similar manner. Recalling that $\psi _0^L = 1$ on $B_{\frac{L}{8}}$, we have

4.97 $$\begin{aligned} \Vert e^{-t'}(1- \psi _0^L )p_{t'}\Vert _{L^2_x} \lesssim e^{- c\frac{L^2}{t'} -t' } \lesssim e^{-cL}, \end{aligned}$$

where the second inequality follows from separately estimating the cases (i) $t' \ll L$, (ii) $t' \sim L$, and (iii) $t' \gg L$. Moreover, since $\psi _0^L = 1$ on $B_{\frac{L}{8}}$, $L \gg R + t$, and $x, y \in B_{2R}$, we have

$$\begin{aligned} |z-y| \ge |z- x| - |x| - |y| \ge \frac{L}{8} - t - 4R \ge \frac{L}{16} \end{aligned}$$

in the domain of integration for $\text {IV}$ in (4.96). Under this condition, we have, as in (4.84),

4.98 $$\begin{aligned} \Vert {\textbf{1}}_{\{|z- y|\ge \frac{L}{16}\}}e^{-t'} {\mathcal {S}}(t) p_{t'}(z-y) \Vert _{L^2_z} \lesssim e^{-cL}. \end{aligned}$$

Then, by applying Cauchy-Schwarz’s inequality with (4.97) and (4.98), we obtain

4.99 $$\begin{aligned} |\text {IV}(x, y; t)| \lesssim e^{-cL}. \end{aligned}$$

A similar computation yields

4.100 $$\begin{aligned} |\text {V}(x, y; t)| \lesssim e^{-cL'}. \end{aligned}$$

Therefore, from (4.96), (4.99), and (4.100), we conclude that

4.101 $$\begin{aligned} | K_{\infty , \infty } (x,y; t) - K_{L, L'}^{(0)}(x,y; t)| \lesssim e^{-cL} + e^{-cL'}. \end{aligned}$$

It remains to estimate $K_{\infty , \infty } (x,y; t)$. In view of (4.95) with (4.42), we have

4.102 $$\begin{aligned} \begin{aligned} K_{\infty , \infty }(x,y; t)&=K^t(x-y) \\ : \!&= \int _{{\mathbb {R}}^2} \frac{(\widehat{{\mathcal {S}}(t)}(\eta ))^2}{\langle 2\pi \eta \rangle ^2}(1 - e^{-\langle 2\pi \eta \rangle ^2}) e^{2\pi i \eta \cdot (x - y)} d \eta . \end{aligned} \end{aligned}$$

From (4.42) with (4.41), we have

4.103 $$\begin{aligned} \big |(\widehat{{\mathcal {S}}(t)}(\eta ))^2(1 - e^{-\langle 2\pi \eta \rangle ^2}) - e^{-t} \cos ^2( t [\hspace{-0.6mm}[ 2\pi \eta ]\hspace{-0.6mm}] )\big |\lesssim \frac{1}{\langle \eta \rangle }, \end{aligned}$$

uniformly in $t \in {\mathbb {R}}_+$. Then, by defining $K^t_1$ by

4.104 $$\begin{aligned} K^t_1(z)= e^{-t} \int _{{\mathbb {R}}^2} \frac{ \cos ^2(t[\hspace{-0.6mm}[ 2\pi \eta ]\hspace{-0.6mm}]) }{\langle 2\pi \eta \rangle ^2} e^{2\pi i\eta \cdot z}d\eta , \end{aligned}$$

it follows from (4.102) and (4.103) that

4.105 $$\begin{aligned} |K_{\infty , \infty }(x,y; t) - K_1^t(x- y)|\lesssim 1, \end{aligned}$$

uniformly in $t \in {\mathbb {R}}_+$ and $x, y \in {\mathbb {R}}^2$.

We now estimate $K_1^t$. Recall from Appendix B.5 in [37] that the Fourier transform of a radial function $F(x) = f(|x|)$ on ${\mathbb {R}}^2$ is given by

4.106 $$\begin{aligned} \widehat{F}(\eta ) = 2\pi \int _0^\infty f(r) J_0 (2\pi r|\eta |) r dr, \end{aligned}$$

where $J_0$ is the Bessel function of order zero, satisfying the following asymptotics as $r \rightarrow \infty$:

4.107 $$\begin{aligned} J_0(r) = \sqrt{\frac{2}{\pi r}} \cos \Big (r- \frac{\pi }{4} \Big ) + O(r^{-\frac{3}{2}}). \end{aligned}$$

See Appendix B.8 in [37] for the asymptotics (4.107). Thus, from (4.104) and (4.106), we have

4.108 $$\begin{aligned} K_1^t(z) = 2\pi e^{-t} \int _0^\infty \frac{ \cos ^2( t [\hspace{-0.6mm}[ 2\pi r ]\hspace{-0.6mm}]) }{ \langle 2\pi r \rangle ^2} J_0(2\pi r|z| ) rdr. \end{aligned}$$

Recalling that $J_0$ is bounded on ${\mathbb {R}}_+$, it follows from (4.108) with (4.107) that

4.109 $$\begin{aligned} \begin{aligned} |K_1^t(z) |&\lesssim e^{-t} \int _0^{ \max ( \frac{1}{|z|} , 1) } \frac{1}{\langle r \rangle } dr + \int _{ \max ( \frac{1}{|z|} , 1) }^\infty \frac{1}{r^{\frac{3}{2}} |z|^{\frac{1}{2}}} dr \\&\lesssim 1+ (- \log |z| )_+ . \end{aligned} \end{aligned}$$

Hence, putting together (4.86), (4.94), (4.101), (4.105), and (4.109), we obtain

4.110 $$\begin{aligned} |K_{L, L'}(x,y; t) | + |K_{\infty , \infty }(x,y; t) | \lesssim 1+ (- \log |x-y| )_+ . \end{aligned}$$

Therefore, the claim (4.74) follows from (4.86), (4.94), (4.101), and (4.110). $\square$

#### Remark 4.8

When $L' = \infty$, we write

4.111 $$\begin{aligned} K_{L, \infty }(x,y; t) = K_{L, \infty }^{(0)}( x,y; t) + K_{L, \infty }^{(1)}( x,y; t), \end{aligned}$$

where

4.112 $$\begin{aligned} \begin{aligned} K_{L, \infty }^{(0)}(x,y; t)&=2 \int _{{\mathbb {R}}^2}\int _0^1 \Big ( e^{-t'} (\psi _0^L p_{t'})* ({\mathcal {S}}(t) \delta _x) \Big )(z)\\&\qquad \qquad \times \big (P(t') {\mathcal {S}}(t) \delta _y \big )(z)dt' dz \end{aligned} \end{aligned}$$

and

$$\begin{aligned} K_{L, \infty }^{(1)}(x,y; t)&=2 \sum _{j = 1}^\infty \int _{{\mathbb {R}}^2}\int _0^1 \Big ( {\textbf{1}}_{[-\frac{L}{2}, \frac{L}{2})^2} \, e^{-t'} (\psi _j^L p_{t'})* ({\mathcal {S}}(t) (\delta _x)^\text {per}_L) \Big )(z)\nonumber \\&\qquad \qquad \times \big (P(t') {\mathcal {S}}(t) \delta _y \big )(z)dt' dz . \end{aligned}$$

Proceeding as in (4.92), we have

$$\begin{aligned} \big \Vert {\textbf{1}}_{[-\frac{L}{2}, \frac{L}{2})^2}(z) \big (P(t') {\mathcal {S}}(t) \delta _y \big )(z)\big \Vert _{H^{-1-\varepsilon }_z} \lesssim L^2 (t')^{-\frac{\varepsilon }{2}}. \end{aligned}$$

Then, proceeding as in (4.93), we obtain

4.113 $$\begin{aligned} |K_{L, \infty }^{(1)}(x,y; t)| \lesssim e^{-cL}. \end{aligned}$$

From (4.95) and (4.112), we have

$$\begin{aligned}&K_{\infty , \infty } (x,y; t) - K_{L, \infty }^{(0)}(x,y; t)\\&\quad = 2 \int _{{\mathbb {R}}^2}\int _0^1 \Big ( e^{-t'} \big ((1- \psi _0^L) p_{t'}\big )* ({\mathcal {S}}(t) \delta _x) \Big )(z) \\&\qquad \qquad \quad \times \Big ( P(t') {\mathcal {S}}(t) \delta _y \Big )(z)dt' dz, \end{aligned}$$

which is precisely $\text {IV}(x, y; t)$ in (4.96). Hence, from (4.99), we have

4.114 $$\begin{aligned} | K_{\infty , \infty } (x,y; t) - K_{L, \infty }^{(0)}(x,y; t)| \lesssim e^{-cL}. \end{aligned}$$

Therefore, putting (4.111), (4.113), and (4.114) together, we obtain

4.115 $$\begin{aligned} | K_{\infty , \infty } (x,y; t) - K_{L, \infty }(x,y; t)| \lesssim e^{-cL}. \end{aligned}$$

On the other hand, from (4.74) and (4.115), we have

4.116 $$\begin{aligned} \begin{aligned} |K_{L, L}(x, y;t) | + |K_{L, \infty }(x, y;t) | + |K_{\infty , \infty }(x, y;t) |&\lesssim 1+ (- \log |x-y| )_+ , \\ | K_{L, L}(x, y;t)- K_{\infty , \infty } (x, y;t)|&\lesssim e^{-cL}. \end{aligned} \end{aligned}$$

A computation analogous to (4.76) with Lemma 2.14, (4.75) (with $L' = \infty$), (4.115), and (4.116) yields

$$\begin{aligned} {\mathbb {E}}&\Big [ \Vert :\!( {\mathcal {S}}(t)Z_L(1) )^m \!: - :\!( {\mathcal {S}}(t)Z_{\infty }(1) )^m \! : \Vert _{W^{-\varepsilon , p_1}(B_R)}^r \Big ] \\&\lesssim C(R, r) e^{-c L} \longrightarrow 0, \end{aligned}$$

as $L \rightarrow \infty$. This proves (4.69).

### Estimates on the enhanced data sets

In this subsection, we establish the following proposition on regularities of the enhanced data sets.

#### Proposition 4.9

(i) Let $R > 0$. Given small $\varepsilon > 0$, there exists finite $p = p(\varepsilon ) \gg 1$ such that the following holds true. Suppose that random *k*-tuple of functions $\Xi _0, \widetilde{\Xi }_0 \in {\mathbb {W}}(R)$ are independent of $\Phi _1 = \Phi _1(u_1, \xi )$ defined in (4.10), where ${\mathbb {W}}(R)$ is as in (4.15). Then, we have

$$\begin{aligned} {\mathbb {E}}_{u_1, \xi , \Xi _0, \widetilde{\Xi }_0}\Big [ \Vert \Xi _1(u_1, \xi ) \Vert _{\Xi _0, \widetilde{\Xi }_0, R} \Big ] \lesssim 1, \end{aligned}$$

where the implicit constant is independent of the distributions of $\Xi _0$ and $\widetilde{\Xi }_0$. Here, $\Xi _1(u_1, \xi )$ is as in (4.13) and $\Vert \cdot \Vert _{\Xi _0, \widetilde{\Xi }_0, R}$ is as in Definition 4.1 (iii).

(ii) Let $\Xi _0(\phi )$ be as in (4.12). Then, given any $\varepsilon > 0$, finite $p \gg 1$, and $\mu > 0$, we have

4.117 $$\begin{aligned} \sup _{L\in {\mathcal {A}}}{\mathbb {E}}_{\rho _L} \Big [ \Vert \Xi _0( \phi ) \Vert _{(L^\infty _t W^{-\varepsilon , p}_\mu )^{\otimes k} } \Big ] <\infty . \end{aligned}$$

We first state and prove an auxiliary lemma. The proof of Proposition 4.9 is presented at the end of this subsection.

#### Lemma 4.10

Let $\Phi _1 = \Phi _1(u_1, \xi )$ be as in (4.10), where $u_1$ and $\xi$ denote the independent spatial and space-time white noises as in (4.1). Define *H*(*x*, *y*; *t*) by

4.118 $$\begin{aligned} H(x, y; t) = {\mathbb {E}}\big [ \Phi _1(x, t) \Phi _1(y, t)\big ]. \end{aligned}$$

Let $$R > 0$$. Then, given small $$\varepsilon > 0$$ and $$1 \le q < \frac{1}{\varepsilon }$$, there exists a finite constant $$C(R) > 0 $$ such that

4.119 $$\begin{aligned} \Vert \langle \nabla _x \rangle ^\varepsilon \langle \nabla _y \rangle ^\varepsilon H(x, y; t) \Vert _{L^{q}_{x, y}(B_R\times B_R)} \le C(R), \end{aligned}$$

uniformly in $$t \in {\mathbb {R}}_+$$.

#### Proof

With a slight abuse of notation, it follows from Definition 3.4 and an analogous property for the space-time white noise $\xi$ that

$$\begin{aligned} H( x-y;t )&\equiv H(x, y; t) \\&= \langle {\mathcal {D}}(t) \delta _x, {\mathcal {D}}(t) \delta _y \rangle _{L^2({\mathbb {R}}^2)} + \int _0^t \langle {\mathcal {D}}(t- t') \delta _x, {\mathcal {D}}(t- t') \delta _y \rangle _{L^2({\mathbb {R}}^2)} dt'. \end{aligned}$$

Then, we have

4.120 $$\begin{aligned} \langle \nabla _x \rangle ^\varepsilon \langle \nabla _y \rangle ^\varepsilon H( x-y;t ) = \langle \nabla _z \rangle ^{2\varepsilon } H(z; t)|_{z = x-y}. \end{aligned}$$

Define $$\widetilde{H}^t(z)$$ by

$$\begin{aligned} \widetilde{H}^t(z) = \int _{{\mathbb {R}}^2} \frac{\sin ^2(t [\hspace{-0.6mm}[ 2\pi \eta ]\hspace{-0.6mm}])}{[\hspace{-0.6mm}[ 2\pi \eta ]\hspace{-0.6mm}]^2} e^{2\pi i\eta \cdot z} d\eta . \end{aligned}$$

Then, from (1.23), we have

4.121 $$\begin{aligned} H(z; t)&=e^{-t} \widetilde{H}^t(z) + \int _0^t e^{-t'} \widetilde{H}^{t'}(z) dt' . \end{aligned}$$

In view of (4.120) and (4.121), it suffices to study

$$\begin{aligned} \langle \nabla \rangle ^{2\varepsilon } \widetilde{H}^t(z) = \int _{{\mathbb {R}}^2}\langle \eta \rangle ^{2\varepsilon } \frac{\sin ^2(t [\hspace{-0.6mm}[ 2\pi \eta ]\hspace{-0.6mm}])}{[\hspace{-0.6mm}[ 2\pi \eta ]\hspace{-0.6mm}]^2} e^{2\pi i\eta \cdot z} d\eta . \end{aligned}$$

As the inverse Fourier transform of a radial function, $\langle \nabla \rangle ^{2\varepsilon } \widetilde{H}^t$ is radial. Hence, from (4.106), we have

$$\begin{aligned} \langle \nabla \rangle ^{2\varepsilon } \widetilde{H}^t(z) = 2\pi \int _0^\infty \langle r \rangle ^{2\varepsilon } \frac{\sin ^2(t [\hspace{-0.6mm}[ 2\pi r ]\hspace{-0.6mm}])}{[\hspace{-0.6mm}[ 2\pi r ]\hspace{-0.6mm}]^2} J_0(2\pi r|z|) r dr, \end{aligned}$$

where $J_0$ is the Bessel function of order zero. Using the boundedness of $J_0$ on ${\mathbb {R}}_+$ and (4.107), we then have

4.122 $$\begin{aligned} \begin{aligned} |\langle \nabla \rangle ^{2\varepsilon } \widetilde{H}^t(z) |&\lesssim \int _0^{ \max ( \frac{1}{|z|} , 1) } \frac{1}{\langle r \rangle ^{1-2\varepsilon }} dr + \int _{ \max ( \frac{1}{|z|} , 1) }^\infty \frac{1}{r^{\frac{3}{2}-2\varepsilon } |z|^{\frac{1}{2}}} dr \\&\lesssim 1 + |z|^{-2\varepsilon }. \end{aligned} \end{aligned}$$

Therefore, from (4.120), (4.121), and (4.122), we obtain

$$\begin{aligned} | \langle \nabla _x \rangle ^{\varepsilon } \langle \nabla _y \rangle ^{\varepsilon } H(x, y; t) | \lesssim 1 + |x-y|^{-2\varepsilon }, \end{aligned}$$

uniformly in $t \in {\mathbb {R}}_+$. Then, by integrating in $x, y \in B_R$, we obtain (4.119), provided that $q < \frac{1}{\varepsilon }$. $\square$

We are now ready to present the proof of Proposition 4.9.

#### Proof of Proposition 4.9

(i) We only show that

4.123 $$\begin{aligned} {\mathbb {E}}\big [ \Vert \Xi _1 (u_1, \xi )\Vert _{\Xi _0, R}\big ] < \infty , \end{aligned}$$

where $\Vert \Xi _1 (u_1, \xi )\Vert _{\Xi _0, R}$ is as in Definition 4.1 (ii). A similar argument yields finiteness of the expectations of the constants $K_2$ and $K_3$ in (4.17) and (4.18), respectively.

Let $j = 1, \dots , k$. Given a function *f* on $B_R\subset {\mathbb {R}}^2$, let $\textbf{f}$ an extension of *f* onto ${\mathbb {R}}^2$. Given a test function $\varphi \in C^\infty _c({\mathbb {R}}^2)$ supported on a ball *B* of radius 1 with $B \subset B_{2R}$, it follows from Lemma 2.13 that

$$\begin{aligned}&{\mathbb {E}}\Big [\big | \langle \textbf{f}:\! \Phi _1^j(t)\!:\, , \varphi \rangle _{L^2({\mathbb {R}}^2)} \big |^2\Big ] \\&=\int _{(B_{2R})^2} \textbf{f}(x) \textbf{f}(y) \varphi (x) \varphi (y) {\mathbb {E}}\big [ : \!\Phi _1^j(x, t))\!: \, : \!\Phi _1^j(y, t)\!: \big ] dxdy \\&= j! \int _{{\mathbb {R}}^4} \textbf{f}(x) \textbf{f}(y) \varphi (x) \varphi (y) \big (H(x, y;t )\big )^j dxdy, \end{aligned}$$

where *H*(*x*, *y*; *t*) is as in (4.118). Then, given small $\varepsilon > 0$, it follows from the duality, Lemma 2.8 (ii), the bi-parameter fractional Leibniz rule (Lemma 2.10), and Lemma 4.10 that there exist finite $p = p(\varepsilon ) \gg 1$ and $q = q(\varepsilon ) \gg 1$ (with $j q < \frac{1}{\varepsilon }\le \frac{p}{2}$) such that

$$\begin{aligned} {\mathbb {E}}&\Big [\big | \langle \textbf{f}: \!\Phi _1^j(t)\!:, \varphi \rangle _{L^2({\mathbb {R}}^2)} \big |^2 \Big ]\\&\lesssim \Vert \textbf{f}\varphi \Vert _{W^{-\varepsilon , q'}(B_{2R})}^2 \big \Vert \langle \nabla _x \rangle ^\varepsilon \langle \nabla _y \rangle ^\varepsilon \big ( H(x, y; t)\big )^j \big \Vert _{L^q_{x, y}(B_{2R} \times B_{2R})}\\&\lesssim \Vert \varphi \Vert _{W^{\varepsilon , q'}(B_{2R})}^2 \Vert \textbf{f}\Vert _{W^{-\varepsilon , p}(B_{2R} )}^2 \Vert \langle \nabla _x \rangle ^\varepsilon \langle \nabla _y \rangle ^\varepsilon H(x, y; t) \Vert ^j_{L^{jq}_{x, y}(B_{2R} \times B_{2R})}\\&\lesssim \Vert \varphi \Vert _{W^{\varepsilon , q'}(B_{2R})}^2 \Vert \textbf{f}\Vert _{W^{-\varepsilon , p}({\mathbb {R}}^2 )}^2. \end{aligned}$$

Hence, by taking an infimum over the extension $\textbf{f}$ and applying Lemma 2.14 (with $j \le k$), we obtain

$$\begin{aligned} {\mathbb {E}}\Big [ \Vert \, f : \!\Phi ^j_1(t) \!:\Vert _{W^{-2(k+1)\varepsilon , p}(B_R)}^p \Big ] \lesssim \Vert f \Vert _{W^{-\varepsilon , p}(B_R)}^p, \end{aligned}$$

uniformly in $t \in {\mathbb {R}}_+$. Then, by first conditioning on $\Xi _0$, we obtain

$$\begin{aligned} {\mathbb {E}}\Bigg [\frac{ \int _0^T \Vert :\! \Phi ^j_1(t)\!: \Xi _{0\ell }(t) \Vert _{W^{-2(k+1)\varepsilon , p}(B_R)}^p dt}{ \int _0^T \Vert \Xi _{0\ell }(t) \Vert _{W^{-\varepsilon , p}(B_R)}^p dt }\Bigg ] \le A_{j, \ell } < \infty . \end{aligned}$$

Hence, we conclude that

$$\begin{aligned} {\mathbb {E}}\big [ \Vert \Xi _1 (u_1, \xi )\Vert _{\Xi _0, R}\big ]&={\mathbb {E}}\Bigg [ \max _{0\le j, \ell \le k} \frac{ \int _0^T \Vert :\! \Phi ^j_1(t)\!: \Xi _{0\ell }(t)\Vert _{W^{-2(k+1)\varepsilon , p}(B_R)}^p dt}{ \int _0^T \Vert \Xi _{0\ell }(t) \Vert _{W^{-\varepsilon , p}(B_R)}^p dt }\Bigg ]\\&\le \sum _{j,\ell = 0}^k A_{j, \ell } < \infty . \end{aligned}$$

This proves (4.123).

(ii) The bound (4.117) follows from the proof of Proposition 4.4. Namely, in view of (4.37), (4.39), and (4.60) with (4.40), (4.56), (4.57), (4.58), and (4.59), it follows from Kolmogorov’s continuity criterion ( [5, Theorem 8.2]) together with the exponential decay in time (see Remark 4.5) that

$$\begin{aligned} \rho _L\Big ( \Vert \Xi _0( \phi ) \Vert _{(L^\infty _t W^{-\varepsilon , p}_\mu )^{\otimes k} }> K \Big ) \lesssim \frac{1}{K^q} \end{aligned}$$

for any finite $q \ge 1$, from which the desired bound (4.117) follows. $\square$

### Global well-posedness on the plane

We are now ready to prove global well-posedness of the hyperbolic $\Phi ^{k+1}_2$-model (4.1) on the plane. We do this by constructing a solution on each cone ${\textbf{C}}_R$, $R >0$.

#### Proposition 4.11

Let $\vec {\rho }_\infty$ be as in Theorem 1.2 (i) and ${{\,\textrm{Law}\,}}(u_0, u_1) = \vec {\rho }_\infty$. Then, given $R>0$, the hyperbolic $\Phi ^{k+1}_2$-model (4.1) is well-posed on the cone ${\textbf{C}}_R$. More precisely, there exists a function *u* of the form (4.8) such that the following statements holds true $\vec {\rho }_\infty$-almost surely:

- $$(u, \partial _tu) \in L^\infty ([0, R]; \vec {H}^{-\varepsilon _k}(B_{R-t}))$$,
- the remainder term $v = u - {\mathcal {S}}(t)u_0 - {\mathcal {D}}(t) u_1 - \Psi$ satisfies the equation (4.11) on ${\textbf{C}}_R$, and $(v, \partial _tv) \in L^\infty ([0, R]; \vec {H}^{1-\varepsilon _k}(B_{R-t}))$,

where $\varepsilon _k = 2(k+1) \varepsilon$ is as in (4.19).

For the convergence of the *L*-periodic solution $u_L$, $L \in {\mathcal {A}}$, to the solution *u* constructed in Proposition 4.11, see Remark 4.12 below.

#### Proof

Let ${\mathcal {A}}= \{ L_j: j \in {\mathbb {N}}\} \subset {\mathbb {N}}$ be the index set such that Theorem 1.2 (i) and Proposition 4.6 hold. Given a spatial white noise $\zeta$ on ${\mathbb {R}}^2$ and a space-time white noise $\xi$ on ${\mathbb {R}}^2\times {\mathbb {R}}_+$, let $\zeta _L$ be the *L*-periodic spatial white noise defined in (3.53) and (3.54), and $\xi _L$ be the *L*-periodic space-time white noise defined in (4.4) and (4.5), $L \ge 1$, respectively.

In Proposition 4.6, we proved that the $L_j$-periodic enhanced Gibbs measure $\nu _{L_j} = (\Xi _0)_\# \rho _{L_j}$ on ${\mathbb {W}}= \big (C({\mathbb {R}}_+; W^{-\varepsilon , p}_\mu ({\mathbb {R}}^2))\big )^{\otimes k}$ converges weakly to $\nu _\infty = (\Xi _0)_\# \rho _\infty$ as $j \rightarrow \infty$. Then, by the Skorokhod representation theorem (Lemma 2.19), there exist a probability space $(\widetilde{\Omega }, \widetilde{\mathcal {F}}, \widetilde{\mathbb {P}})$, and random variables $\Xi _0^j, \Xi _0:\widetilde{\Omega }\rightarrow {\mathbb {W}}$ such that

4.124 $$\begin{aligned} {{\,\textrm{Law}\,}}( \Xi _0^j) = \nu _{L_j} \qquad \text {and}\qquad {{\,\textrm{Law}\,}}(\Xi _0) = \nu _\infty , \end{aligned}$$

and $\Xi _0^j$ converges $\widetilde{\mathbb {P}}$-almost surely to $\Xi _0$ in ${\mathbb {W}}$ as $j \rightarrow \infty$. Given $j \in {\mathbb {N}}$, we define a transport plan ${\mathfrak {p}}_{L_j} \in \Pi (\nu _\infty , \nu _{L_j})$ by

4.125 $$\begin{aligned} {\mathfrak {p}}_{L_j} = (\Xi _0, \Xi _0^j)_\#\widetilde{\mathbb {P}}. \end{aligned}$$

Then, by the bounded convergence theorem, we have

4.126 $$\begin{aligned} \begin{aligned} \int _{{\mathbb {W}}\times {\mathbb {W}}} d_{\mathbb {W}}( \Theta , \Theta ' ) d{\mathfrak {p}}_{L_j}(\Theta , \Theta ')&= \int _{\widetilde{\Omega }} d_{\mathbb {W}}(\Xi _0(\widetilde{\omega }), \Xi _0^j(\widetilde{\omega })) d\widetilde{\mathbb {P}}(\widetilde{\omega })\\&\longrightarrow 0, \end{aligned} \end{aligned}$$

as $j \rightarrow \infty$, where the metric $d_{\mathbb {W}}$ is as in (4.14). See Remark 6.11 in [70].

From (4.36) and (4.61) with (4.12), we have

4.127 $$\begin{aligned} \rho _{L_j} = (\text {eval}_{t = 0}\circ \text {proj}_1 \big )_\# \nu _{L_j} \qquad \text {and} \qquad \rho _{\infty } = (\text {eval}_{t = 0}\circ \text {proj}_1 \big )_\# \nu _{\infty } , \end{aligned}$$

where $\text {eval}_{t = 0}$ denotes the evaluation map at time $t = 0$ and $\text {proj}_1$ is the projection onto the first component. We now define the first components of the initial data by

4.128 $$\begin{aligned} u_{0, L_j} = \text {eval}_{t = 0}\circ \text {proj}_1(\Xi _0^j) \qquad \text {and} \qquad u_{0} = \text {eval}_{t = 0}\circ \text {proj}_1(\Xi _0). \end{aligned}$$

Then, from (4.124), (4.127), and (4.128), we have

$$\begin{aligned} {{\,\textrm{Law}\,}}( u_{0, L_j}) = \rho _{L_j} \qquad \text {and}\qquad {{\,\textrm{Law}\,}}(u_{0}) = \rho _\infty . \end{aligned}$$

Finally, we let

- $\vec {u} = (u, \partial _tu) = \vec {u}(u_0, u_1, \xi )$ denote a solution to the hyperbolic $\Phi ^{k+1}_2$-model (4.1) on ${\mathbb {R}}^2$ with $u_0$ as in (4.128) and $u_1 = \zeta$, and
- $\vec {u}_{L_j} = (u_{L_j}, \partial _tu_{L_j}) = \vec {u}_{L_j}(u_{0, L_j}, u_{1, L_j}, \xi _{L_j})$ denote a solution to the $L_j$-periodic hyperbolic $\Phi ^{k+1}_2$-model (4.2) on ${\mathbb {R}}^2$ with $u_{0, L_j}$ as in (4.128) and $u_{1, L_j} = \zeta _{L_j}$.

In the following, we fix $R \in {\mathbb {N}}$ and work on the cone ${\textbf{C}}_R$. When $L_j \gg R$, it follows from the finite speed of propagation and (4.6) with the notation above that

4.129 $$\begin{aligned} \vec {u}_{L_j}(u_{0, {L_j}}, u_{1, {L_j}}, \xi _{L_j}) = \vec {u}_{L_j}(u_{0,{L_j}}, u_{1}, \xi ) \quad \text {on the cone }{\textbf{C}}_R. \end{aligned}$$

In particular, on ${\textbf{C}}_R$, $\vec {u}_{L_j}(u_{0, {L_j}}, u_{1}, \xi )$ satisfies (4.1) with the initial data $(u_{0, {L_j}}, u_1)$. We denote by ${\mathbb {P}}_{u_1, \xi } = {\mathbb {P}}_{u_1} \otimes {\mathbb {P}}_\xi$ the probability distribution of the spatial white noise $u_1$ and the space-time white noise $\xi$ which are independent from each other and also from the transport plan ${\mathfrak {p}}_{L_j}$, $j \in {\mathbb {N}}$, defined in (4.125). For simplicity of notation, we set ${\mathcal {X}}= {\mathcal {D}}'({\mathbb {R}}^2) \times {\mathcal {D}}'({\mathbb {R}}^2\times {\mathbb {R}}_+)$, where $(u_1, \xi )$ lives.

Given $\Theta = (\Theta _{1}, \cdots , \Theta _{k}) \in {\mathbb {W}}$, let $v = v(\Theta , u_1, \xi )$ be a solution to (4.11), where we replace $:\! \Phi _0^m(u_0) \!:$ by $\Theta _{m}$. Namely, $v = v(\Theta , u_1, \xi )$ satisfies

4.130 $$\begin{aligned} \begin{aligned} v(t)&= - \sum _{\ell = 0}^k \sum _{m = 0}^\ell {k\atopwithdelims ()\ell } {\ell \atopwithdelims ()m}\\&\times \int _0^t {\mathcal {D}}(t - t')\big ( \Theta _{m}(t') :\! \Phi _1^{\ell -m}(u_1, \xi ) (t')\!: v^{k- \ell }(t')\big ) dt'. \end{aligned} \end{aligned}$$

Define $$E_R \subset {\mathbb {W}}\times {\mathcal {X}}$$ by

$$\begin{aligned} E_R = \big \{&(\Theta , u_1, \xi ) \in {\mathbb {W}}\times {\mathcal {X}}: \text {there exists a solution }v = v(\Theta , u_1, \xi )\text { to }(4.130) \\&\text {on the cone }{\textbf{C}}_R\text { such that }\vec {v} = (v, \partial _tv) \in L^\infty ([0, R]; \vec {H}^{1-\varepsilon _k}(B_{R-t})) \big \}. \end{aligned}$$

Our goal is to show

4.131 $$\begin{aligned} \rho _\infty \otimes {\mathbb {P}}_{u_1, \xi } \bigg (\bigcap _{R \in {\mathbb {N}}} \big \{(u_0, u_1, \xi ): \big (\Xi _0(u_0) , u_1, \xi \big ) \in E_R \big \}\bigg ) = 1, \end{aligned}$$

where $\Xi _0(\cdot )$ is the map defined in (4.12).

Fix $R \in {\mathbb {N}}$. Given $M_0, M_1, M_2 \ge 1$ and $\delta > 0$, define

4.132 $$\begin{aligned} \begin{aligned} A_{R, M_0, M_2}&= \Big \{(\Theta ', u_1, \xi ) \in {\mathbb {W}}\times {\mathcal {X}}: \Vert \Theta ' \Vert _{{\mathbb {W}}(R)} \le M_0, \\&\qquad \qquad \Vert \vec {v}(\Theta ', u_1, \xi )\Vert _{L^\infty ([0, R]; \vec {H}^{1-\varepsilon _k}(B_{R - t}))} \le M_2 \Big \}, \\ B_{R, M_1}&= \Big \{ (\Theta , \Theta ', u_1, \xi ) \in {\mathbb {W}}\times {\mathbb {W}}\times {\mathcal {X}}: \Vert \Xi _1(u_1, \xi ) \Vert _{\Theta , \Theta ', R} \le M_1\Big \}, \\ C_{R, \delta }&= \Big \{ (\Theta , \Theta ') \in {\mathbb {W}}\times {\mathbb {W}}: d_{\mathbb {W}}( \Theta , \Theta ' ) \le \delta \Big \}, \end{aligned} \end{aligned}$$

where $\Theta ' = (\Theta _{1}', \cdots , \Theta _{k}')$, the ${\mathbb {W}}(R)$-norm is as in (4.15), and $\Vert \cdot \Vert _{\Theta , \Theta ', R}$ is as in Definition 4.1 (iii). Furthermore, we define $\widetilde{E}_R$, $\widetilde{A}_{R, M_0, M_2}$, and $\widetilde{C}_{R, \delta }$ by setting

4.133 $$\begin{aligned} \begin{aligned} \widetilde{E}_R&= \big \{(\Theta , \Theta ', u_1, \xi ) \in {\mathbb {W}}\times {\mathbb {W}}\times {\mathcal {X}}: (\Theta , u_1, \xi ) \in E_R \big \}, \\ \widetilde{A}_{R, M_0, M_2}&= \big \{(\Theta , \Theta ', u_1, \xi ) \in {\mathbb {W}}\times {\mathbb {W}}\times {\mathcal {X}}: (\Theta ', u_1, \xi ) \in A_{R, M_0, M_2}\big \}, \\ \widetilde{C}_{R, \delta }&= \big \{(\Theta , \Theta ', u_1, \xi ) \in {\mathbb {W}}\times {\mathbb {W}}\times {\mathcal {X}}: (\Theta , \Theta ') \in C_{R, \delta }\big \} \end{aligned} \end{aligned}$$

such that all the sets live in a common space ${\mathbb {W}}\times {\mathbb {W}}\times {\mathcal {X}}$. Then, it follows from Propositions 4.2 and  4.3 that

4.134 $$\begin{aligned} \begin{aligned} \widetilde{E}_R \supset \widetilde{A}_{R, M_0,M_2} \cap B_{R, M_1} \cap \widetilde{C}_{R, \delta }, \end{aligned} \end{aligned}$$

for any $M_0, M_1, M_2 \ge 1$ and $0 < \delta \le \delta ^* = \delta ^* (\mu , R, M_0, M_1, M_2)$. Here, $\delta ^* = \delta ^* (\mu , R, M_0, M_1, M_2)$ is chosen such that, in view of (4.14) and (4.15),

4.135 $$\begin{aligned} d_{\mathbb {W}}(f, g) \le \delta ^* \quad \text {implies}\quad \Vert f - g\Vert _{{\mathbb {W}}(R)} \le \delta _*, \end{aligned}$$

where $\delta _* = \delta _* (R, M_0, M_1, M_2)> 0$ is as in Proposition 4.3.^38^

Recalling (2.59) that

4.136 $$\begin{aligned} \int _{\Theta ' \in {\mathbb {W}}} d{\mathfrak {p}}_{L_j}(\Theta , \Theta ') = d\nu _\infty (\Theta ) \quad \text {and} \quad \int _{\Theta \in {\mathbb {W}}} d{\mathfrak {p}}_{L_j}(\Theta , \Theta ') = d\nu _{L_j}(\Theta '), \end{aligned}$$

it follows from (4.136), (4.133), (4.134), and (4.132) that

4.137 $$\begin{aligned} \rho _\infty&\otimes {\mathbb {P}}_{u_1, \xi } \Big (\big \{(u_0, u_1, \xi ): \big (\Xi _0(u_0) , u_1, \xi \big ) \in E_R \big \}\Big )\nonumber \\&= \int _{\mathcal {X}}\int _{{\mathbb {W}}\times {\mathbb {W}}} {\textbf{1}}_{(\Theta , u_1, \xi ) \in E_R}(\Theta , \Theta ', u_1, \xi ) d {\mathfrak {p}}_{L_j} (\Theta , \Theta ') d{\mathbb {P}}_{u_1, \xi }(u_1, \xi )\nonumber \\&= \int _{\mathcal {X}}\int _{{\mathbb {W}}\times {\mathbb {W}}} {\textbf{1}}_{\widetilde{E}_R}(\Theta , \Theta ', u_1, \xi ) d {\mathfrak {p}}_{L_j} (\Theta , \Theta ') d{\mathbb {P}}_{u_1, \xi }(u_1, \xi )\nonumber \\&\ge \int _{\mathcal {X}}\int _{{\mathbb {W}}\times {\mathbb {W}}} {\textbf{1}}_{\widetilde{A}_{R, M_0,M_2} \cap B_{R, M_1} \cap \widetilde{C}_{R, \delta }} (\Theta , \Theta ', u_1, \xi ) d {\mathfrak {p}}_{L_j} (\Theta , \Theta ') d{\mathbb {P}}_{u_1, \xi }(u_1, \xi )\nonumber \\&\ge 1 - \int _{{\mathbb {W}}\times {\mathbb {W}}} {\textbf{1}}_{\{\Vert \Theta ' \Vert _{{\mathbb {W}}(R)}> M_0\}} (\Theta ') d {\mathfrak {p}}_{L_j} (\Theta , \Theta ') \nonumber \\&\quad - \int _{\mathcal {X}}\int _{{\mathbb {W}}\times {\mathbb {W}}} {\textbf{1}}_{ \{ \Vert \vec {v}(\Theta ', u_1, \xi )\Vert _{L^\infty ([0, R]; \vec {H}^{1-\varepsilon _k}(B_{R - t}))} > M_2\}} (\Theta ', u_1, \xi ) \nonumber \\&d {\mathfrak {p}}_{L_j} (\Theta , \Theta ') d{\mathbb {P}}_{u_1, \xi }(u_1, \xi ) \nonumber \\&\quad - \int _{\mathcal {X}}\int _{{\mathbb {W}}\times {\mathbb {W}}} {\textbf{1}}_{B_{R, M_1}^c} (\Theta , \Theta ', u_1, \xi ) d {\mathfrak {p}}_{L_j} (\Theta , \Theta ') d{\mathbb {P}}_{u_1, \xi }(u_1, \xi ) \nonumber \\&\quad - \int _{{\mathbb {W}}\times {\mathbb {W}}} {\textbf{1}}_{C_{R, \delta }^c} (\Theta , \Theta ') d {\mathfrak {p}}_{L_j} (\Theta , \Theta ') \nonumber \\&=: 1 - \hspace{0.5mm}\text {I}\hspace{0.5mm} - \text {II} - \text {III} - \text {IV}. \end{aligned}$$

From (4.136), (4.36), (4.127), Markov’s inequality, and Proposition 4.9 (ii), we have

4.138 $$\begin{aligned} \begin{aligned} \hspace{0.5mm}\text {I}\hspace{0.5mm}&\lesssim _{\mu , R} \frac{1}{M_0} {\mathbb {E}}_{\rho _{L_j}}\Big [ \Vert \Xi _0(u_{0, L_j}) \Vert _{(L^\infty _t W^{-\varepsilon , p}_\mu )^{\otimes k} } \Big ] \lesssim \frac{1}{M_0}, \end{aligned} \end{aligned}$$

uniformly in $j \in {\mathbb {N}}$. Similarly, from (4.136), (4.36), (4.127), and Markov’s inequality, we have

4.139 $$\begin{aligned} \text {II} < \frac{1}{M_2} {\mathbb {E}}_{\rho _{L_j}\otimes {\mathbb {P}}_{u_1, \xi }} \Big [\Vert \vec {v}(\Xi _0(u_{0, L_j}), u_1, \xi )\Vert _{L^\infty ([0, R]; \vec {H}^{1-\varepsilon _k}(B_{R - t}))}\Big ]. \end{aligned}$$

In view of (4.129), we have

$$\begin{aligned} \vec {v}(\Xi _0(u_{0, L_j}), u_1, \xi ) = \vec {u}_{L_j} (u_{0, L_j}, u_1, \xi ) - {\mathcal {S}}(t)u_{0, L_j} + {\mathcal {D}}(t) u_1 + \Psi (t) \end{aligned}$$

on ${\textbf{C}}_R$, where $u_{L_j}$ is the solution to the $L_j$-periodic hyperbolic $\Phi ^{k+1}$-model (4.2). Thus, we see that $v(\Xi _0(u_{0, L_j}), u_1, \xi )$ satisfies

4.140 $$\begin{aligned} v(\Xi _0(u_{0, L_j}), u_1, \xi )(t)&= - \int _0^t {\mathcal {D}}(t-t') ({\textbf{1}}_{{\textbf{C}}_R}: \!u_{L_j}^k(t')\! : ) dt' \end{aligned}$$

on ${\textbf{C}}_R$. Compare this with (4.7), (4.8), and (4.9) (with an additional cutoff function ${\textbf{1}}_{{\textbf{C}}_R}$ as in (4.24)). Then, from (4.140) Minkowski’s integral inequality, and Hölder’s inequality, we have

4.141 $$\begin{aligned} \begin{aligned}&\Vert v(\Xi _0(u_{0, L_j}), u_1, \xi )\Vert _{L^\infty ([0, R];\vec {H}^{1-\varepsilon _k}(B_{R - t}))}\\&\quad \le \bigg \Vert \int _0^{t} \big \Vert {\mathcal {D}}(t-t')({\textbf{1}}_{{\textbf{C}}_R} : \!u_{L_j}^k(t')\!:) \Vert _{H^{1-\varepsilon _k}(B_{R - t})} dt' \bigg \Vert _{L^\infty ([0, R])} \\&\quad \lesssim \int _0^R \Vert :\!u_{L_j}^k(t')\!: \Vert _{H^{-\varepsilon _k}(B_{R - t'})} dt' . \end{aligned} \end{aligned}$$

Hence, from (4.139), (4.141), the invariance $\vec {\rho }_{L_j}$ under (4.2), and Proposition 4.9 (ii), we obtain

4.142 $$\begin{aligned} \begin{aligned} \text {II}&\lesssim _{\mu , R} \frac{1}{M_2} {\mathbb {E}}_{\rho _{L_j}}\Big [ \Vert \Xi _0(u_{0, L_j}) \Vert _{L^\infty _t (W^{-\varepsilon , p}_\mu )^{\otimes k} } \Big ] \lesssim \frac{1}{M_2}, \end{aligned} \end{aligned}$$

uniformly in $$j \in {\mathbb {N}}$$.

From (4.132) and Proposition 4.9 (i), we have

4.143 $$\begin{aligned} \begin{aligned} \text {III}&< \frac{1}{M_1} {\mathbb {E}}_{ {\mathfrak {p}}_{L_j} \otimes {\mathbb {P}}_{u_1, \xi }} \Big [ \Vert \Xi _1(u_1, \xi ) \Vert _{\Theta , \Theta ' , R} \Big ] \lesssim \frac{1}{M_1}, \end{aligned} \end{aligned}$$

uniformly in $j \in {\mathbb {N}}$. Finally, from (4.132) and (4.126), we have, for each fixed $\delta > 0$,

4.144 $$\begin{aligned} \text {IV}&< \frac{1}{\delta }\int _{{\mathbb {W}}\times {\mathbb {W}}} d_{\mathbb {W}}( \Theta , \Theta ' ) d {\mathfrak {p}}_{L_j} (\Theta , \Theta ') \longrightarrow 0, \end{aligned}$$

as $$j \rightarrow \infty $$.

Fix small $\kappa > 0$. We first choose $M_0, M_1, M_2 \gg 1$ such that (4.138), (4.142), and (4.143) imply

4.145 $$\begin{aligned} \hspace{0.5mm}\text {I}\hspace{0.5mm} + \text {II} + \text {III}< \frac{\kappa }{2}. \end{aligned}$$

Then, we choose sufficiently small $\delta = \delta (R, M_0, M_1, M_2)> 0$ such that $0 < \delta \le \delta ^*$ (and thus (4.137) holds). Finally, by taking sufficiently large $j \gg 1$, we obtain from (4.144) that

4.146 $$\begin{aligned} \text {IV} < \frac{\kappa }{2}. \end{aligned}$$

Hence, we obtain ${\mathbb {P}}(E_R^c) < \kappa$. Since the choice of $\kappa > 0$ was arbitrary, we then conclude from (4.137), (4.145), and (4.146) that

$$\begin{aligned} \rho _\infty \otimes {\mathbb {P}}_{u_1, \xi } \Big (\big \{(u_0, u_1, \xi ): \big (\Xi _0(u_0) , u_1, \xi \big ) \in E_R \big \}\Big ) = 1 \end{aligned}$$

for any $R \in {\mathbb {N}}$, which in turn implies (4.131). This concludes the proof of Proposition 4.11. $\square$

#### Remark 4.12

A slight modification of the proof of Proposition 4.11 shows that, on each cone ${\textbf{C}}_R$, $R> 0$, the $L_j$-periodic solution $\vec {u}_{L_j} = (u_{L_j}, \partial _tu_{L_j})$ to (4.2) converges in probability to the solution $\vec {u} = (u, \partial _tu)$ to (4.1) constructed in Proposition 4.11 in the $L^\infty ([0, R]; \vec {H}^{-\varepsilon _k}(B_{R-t}))$-topology. In particular, there exists a subsequence $\{u_{L_{j_\ell }}\}_{\ell \in {\mathbb {N}}}$ such that $\vec {u}_{L_{j_\ell }}$ converges almost surely to $\vec {u}$ in $L^\infty ([0, R]; \vec {H}^{-\varepsilon _k}(B_{R-t}))$ as $\ell \rightarrow \infty$. Since $\vec {u}_{L_{j_\ell }} \in C({\mathbb {R}}_+; \vec {H}^{-\varepsilon _k}_\text {loc}({\mathbb {R}}^2))$, we then deduce that $u \in C(\big [0, \tfrac{R}{2}\big ]; \vec {H}^{-\varepsilon _k}(B_{ R/2}))$ almost surely. Since the choice of $R> 0$ was arbitrary, we conclude that $(u, \partial _tu) \in C({\mathbb {R}}_+; \vec {H}^{-\varepsilon _k}_loc ({\mathbb {R}}^2))$ almost surely.

Let $u_0$ and $u_{0, L_j}$ be as in (4.128). Then, define a set $F_R \subset \widetilde{\Omega }\times {\mathcal {X}}$ by

4.147 $$\begin{aligned} \begin{aligned}&F_R = \bigcup _{M_0, M_1, M_2 = 1}^\infty \, \bigcap _{\begin{array}{c} m \in {\mathbb {N}}\\ m \ge \delta ^*(\mu , R, M_0, M_1, M_2)^{-1} \end{array}} \bigg (\bigcap _{\ell = 1}^\infty \bigcup _{j = \ell }^\infty \big (D_{R, M_0, M_1, M_2}^{L_j} \cap C_{R, \frac{1}{m} }^{L_j}\big ) \Big \}\bigg ), \end{aligned} \end{aligned}$$

where $\delta ^*(\mu , R, M_0, M_1, M_2)$ is as in the proof of Proposition 4.11 (see also (4.135)). Here, $D_{R, M_0, M_1, M_2}^{L_j}$ and $C_{R, \delta }^{L_j}$ are given by

4.148 $$\begin{aligned} \begin{aligned} D_{R, M_0, M_1, M_2}^{L_j}&= \Big \{ \big ( \Xi _0(u_0(\widetilde{\omega })), \Xi _0(u_{0, L_j}(\widetilde{\omega })), u_1, \xi \big ) \in \widetilde{A}_{R, M_0,M_2} \cap B_{R, M_1} \big )\Big \}, \\ C_{R, \delta }^{L_j}&= \Big \{ \big ( \Xi _0(u_0(\widetilde{\omega })), \Xi _0(u_{0, L_j}(\widetilde{\omega }))\big ) \in \widetilde{C}_{R, \delta } \Big \}, \end{aligned} \end{aligned}$$

where $\widetilde{A}_{R, M_0,M_2}^{L_j}$, $B_{R, M_1}^{L_j}$, and $\widetilde{C}_{R, \delta }^{L_j}$ are as in (4.132) and (4.133). As in (4.134), we then have

$$\begin{aligned} F_R \subset \Big \{ ( \widetilde{\omega }, u_1, \xi ) \in \widetilde{\Omega }\times {\mathcal {X}}: \big (\Xi _0(u_0(\widetilde{\omega })), u_1, \xi \big )\in E_R \Big \}. \end{aligned}$$

Given small $\kappa > 0$, by proceeding as in (4.137) with the uniform (in *j*) bounds (4.138), (4.142), and (4.143), choosing sufficiently large $M_0, M_1, M_2 \gg 1$, and then applying Markov’s inequality and (4.126), we obtain

$$\begin{aligned} \widetilde{\mathbb {P}}\otimes {\mathbb {P}}_{u_1, \xi } (F_R^c)&\le \kappa + \sup _{\begin{array}{c} m \in {\mathbb {N}}\\ m \ge \delta ^*(\mu , R, M_0, M_1, M_2)^{-1} \end{array}} \liminf _{j \rightarrow \infty } {\mathfrak {p}}_{L_j} \Big ( d_{\mathbb {W}}\big ( \Theta , \Theta ' ) \big ) > \tfrac{1}{m} \Big )\\&\le \kappa + \sup _{\begin{array}{c} m \in {\mathbb {N}}\\ m \ge \delta ^*(\mu , R, M_0, M_1, M_2)^{-1} \end{array}} m \cdot \liminf _{j \rightarrow \infty } \int _{{\mathbb {W}}\times {\mathbb {W}}} d_{\mathbb {W}}( \Theta , \Theta ' ) d {\mathfrak {p}}_{L_j} (\Theta , \Theta ') \\&= \kappa . \end{aligned}$$

Since the choice of $\kappa > 0$ was arbitrary, we conclude that

$$\begin{aligned} \widetilde{\mathbb {P}}\otimes {\mathbb {P}}_{u_1, \xi }(F_R) = 1. \end{aligned}$$

With a slight abuse of notation, let us denote by $\omega '$ an element $(\widetilde{\omega }, u_1, \xi ) \in \widetilde{\Omega }\times {\mathcal {X}}$. Given $\omega ' \in F_R$, it follows from the definition (4.147) of $F_R$ with (4.134) and Propositions 4.2 and 4.3 that there exists an $\omega '$-dependent subsequence $L_{j_\ell } \subset {\mathcal {A}}$ such that $\vec {u}_{L_{j_\ell }}(\omega ')$ converges almost surely to $\vec {u}(\omega ')$ in $L^\infty ([0, R]; \vec {H}^{-\varepsilon _k}(B_{R-t}))$ as $\ell \rightarrow \infty$. On the other hand, we claim that $\{u_{L_j}\}_{j \in {\mathbb {N}}}$ is Cauchy in probability in $L^\infty ([0, R]; \vec {H}^{-\varepsilon _k}(B_{R-t}))$. Then, from the uniqueness of a limit, we conclude that $\vec {u}_{L_j}$ converges in probability to $\vec {u}$ in $L^\infty ([0, R]; \vec {H}^{-\varepsilon _k}(B_{R-t}))$ as $j \rightarrow \infty$.

It remains to show that $\{\vec {u}_{L_j}\}_{j \in {\mathbb {N}}}$ is Cauchy in probability (with respect to $\widetilde{\mathbb {P}}\otimes {\mathbb {P}}_{u_1, \xi }$) in $L^\infty ([0, R]; \vec {H}^{-\varepsilon _k}(B_{R-t}))$. From (4.126) with (4.128), we have, for each fixed $\delta _0 > 0$,

4.149 $$\begin{aligned} \begin{aligned} \widetilde{\mathbb {P}}&\Big ( d_{\mathbb {W}}\big ( \Xi _0(u_{0, L_{j_1}}), \Xi _0(u_{0, L_{j_2}} ) \big )> \delta _0 \Big )\\&\le \widetilde{\mathbb {P}}\Big ( d_{\mathbb {W}}\big ( \Xi _0(u_{0, L_{j_1}}), \Xi _0(u_{0} ) \big )> \tfrac{\delta _0}{2} \Big ) + \widetilde{\mathbb {P}}\Big ( d_{\mathbb {W}}\big ( \Xi _0(u_{0}), \Xi _0(u_{0, L_{j_2}} ) \big ) > \tfrac{\delta _0}{2} \Big )\\&< \frac{2}{\delta _0} \int _{\widetilde{\Omega }} d_{\mathbb {W}}(\Xi _0^{j_1}(\widetilde{\omega }), \Xi _0(\widetilde{\omega })) d\widetilde{\mathbb {P}}(\widetilde{\omega }) + \frac{2}{\delta _0} \int _{\widetilde{\Omega }} d_{\mathbb {W}}(\Xi _0(\widetilde{\omega }), \Xi _0^{j_2}(\widetilde{\omega })) d\widetilde{\mathbb {P}}(\widetilde{\omega })\\&\longrightarrow 0, \end{aligned} \end{aligned}$$

as $j_1, j_2 \rightarrow \infty$. Given $\kappa > 0$, from (4.138), (4.142), and (4.143), there exist $M_0, M_1, M_2 \gg 1$ such that

4.150 $$\begin{aligned} \widetilde{\mathbb {P}}\otimes {\mathbb {P}}_{u_1, \xi }\Big ( (D_{R, M_0, M_1, M_2}^{L_{j_1}})^c \cup (D_{R, M_0, M_1, M_2}^{L_{j_2}})^c \Big ) < \frac{\kappa }{2}, \end{aligned}$$

uniformly in $j \in {\mathbb {N}}$, where $D_{R, M_0, M_1, M_2}^{L_j}$ is as in (4.148). Then, it follows from Proposition 4.3 with (4.149) and (4.150) (see also (4.6)) that

$$\begin{aligned}&\widetilde{\mathbb {P}}\otimes {\mathbb {P}}_{u_1, \xi } \Big ( \big \Vert \vec {u}_{L_{j_1}}(u_{0, L_{j_1}}(\widetilde{\omega }), u_1, \xi ) - \vec {u}_{L_{j_2}} (u_{0, L_{j_2}}(\widetilde{\omega }), u_1, \xi ) \big \Vert _{L^\infty ([0, R]; \vec {H}^{-\varepsilon _k}(B_{R-t}))}> \delta \Big ) \\&\quad \le \widetilde{\mathbb {P}}\Big ( d_{\mathbb {W}}\big ( \Xi _0(u_{0, L_{j_1}}), \Xi _0(u_{0, L_{j_2}} ) \big ) > \delta _0 \Big )\\&\quad \quad + \widetilde{\mathbb {P}}\otimes {\mathbb {P}}_{u_1, \xi } \Big ( (D_{R, M_0, M_1, M_2}^{L_{j_1}})^c \cup (D_{R, M_0, M_1, M_2}^{L_{j_2}})^c \Big )\\&\quad < \kappa \end{aligned}$$

for any $$j_2 \ge j_1 \gg 1$$. Here, $$\delta _0 = \delta _0(\delta ) > 0$$ is a small number such that $$ d_{\mathbb {W}}(f, g) \le \delta _0 $$ implies $$\Vert f - g\Vert _{{\mathbb {W}}(R)} \le \frac{1}{2} C_0^{-1}\delta $$, where $$C_0 = C_0(R, M_0, M_1, M_2)> 0 $$ are as in Proposition 4.3. This shows that $$\{\vec {u}_{L_j}\}_{j \in {\mathbb {N}}}$$ is Cauchy in probability in $$L^\infty ([0, R]; \vec {H}^{-\varepsilon _k}(B_{R-t}))$$.

### Invariance of the gibbs measure $\vec {\rho }_\infty$

We conclude this section by briefly discussing invariance of the Gibbs measure $\vec {\rho }_\infty$ under the dynamics of the hyperbolic $\Phi ^{k+1}_2$-model (4.1) on the plane. We only consider the values of *L* belonging to ${\mathcal {A}}= \{ L_j: j \in {\mathbb {N}}\} \subset {\mathbb {N}}$, along which we proved Theorem 1.2 (i).

Let *u* be the solution to the hyperbolic $\Phi ^{k+1}_2$-model (4.1) with ${{\,\textrm{Law}\,}}(u_{0}, u_{1}) = \vec {\rho }_\infty$. Our goal is to show

4.151 $$\begin{aligned} {{\,\textrm{Law}\,}}(\vec {u}(t)) = \vec {\rho }_\infty \end{aligned}$$

for each $t \in {\mathbb {R}}_+$. Fix $t > 0$. Given $j \in {\mathbb {N}}$, let $u_{L_j}$ be the solution to the $L_j$-periodic hyperbolic $\Phi ^{k+1}_2$-model (4.2) with ${{\,\textrm{Law}\,}}(u_{0, L_j}, u_{1, L_j}) = \vec {\rho }_{L_j}$ such that $(u_{0, L_j}, u_{1, L_j})$ converges almost surely to $(u_{0}, u_{1})$ in $\vec {H}_loc ^{-\varepsilon }({\mathbb {R}}^2)$, as $j \rightarrow \infty$. By the invariance of the Gibbs measure $\vec {\rho }_{L_j}$ under the $L_j$-periodic dynamics, we have ${{\,\textrm{Law}\,}}(\vec {u}_{L_j}(t)) = \vec {\rho }_{L_j}$ for any $t \in {\mathbb {R}}_+$. Then, it follows from the weak convergence of $\{ \vec {\rho }_{L_j}\}_{j \in {\mathbb {N}}}$ to $\vec {\rho }_{\infty }$ that

4.152 $$\begin{aligned} \vec {\rho }_\infty = \mathop {\mathrm {w-lim}}\limits _{j \rightarrow \infty }{{\,\textrm{Law}\,}}(\vec {u}_{L_j}(t)), \end{aligned}$$

where $\mathop {\mathrm {w-lim}}\limits$ denotes a weak limit of probability measures.

On the other hand, given $R> t$, let $\vec {\varphi } = (\varphi _0, \varphi _1)\in ({\mathcal {D}}({\mathbb {R}}^2))^{\otimes 2} = (C_c^\infty ({\mathbb {R}}^2))^{\otimes 2}$ be a pair of test functions with $\mathop {\textrm{supp}}\limits \varphi _\ell \subset B_R$, $\ell = 1, 2$. Then, it follows from the proof of Proposition 4.11 (see Remark 4.12) that the solution $\vec {u}_{L_j}$ to (4.2) converges in probability to the solution $\vec {u}$ to (4.1) on ${\textbf{C}}_{R + t}$ as $j \rightarrow \infty$. This in particular implies $\langle \vec {u}_{L_j}(t), \vec {\varphi } \rangle$ converges in probability to $\langle \vec {u}(t), \vec {\varphi } \rangle$ as $j \rightarrow \infty$, where $\langle \cdot , \cdot \rangle$ denotes $({\mathcal {D}}'({\mathbb {R}}^2))^{\otimes 2}$-$({\mathcal {D}}({\mathbb {R}}^2))^{\otimes 2}$ duality pairing. In particular, as a $({\mathcal {D}}'({\mathbb {R}}^2))^{\otimes 2}$-valued random variable $\vec {u}_{L_j}(t)$ converges in law to $\vec {u}(t)$ and, therefore, together with (4.152) and the uniqueness of a limit, we obtain (4.151). This concludes the proof of Theorem 1.2 (ii).

## Data Availability

This manuscript has no associated data.

## Appendix A Embeddings Between Weighted Sobolev and Besov Spaces

In this appendix, we present the proof of Lemma 2.3. Fix $1\le p < \infty$. We first prove

A.1 $$\begin{aligned} \Vert f \Vert _{W^{s, p}_\mu } \lesssim \Vert f\Vert _{B^{s', \mu '}_{p, 1}}. \end{aligned}$$

for $s < s'$ and $\mu \ge c(p) \mu ' > 0$. Let us state an auxiliary lemma.

### Lemma A.1

Let $s \in {\mathbb {R}}$, finite $p \ge 1$, and $\mu > 0$. Then, given any $\varepsilon > 0$, there exists $c_0 > 0$ such that

A.2 $$\begin{aligned} \Vert \phi _j {\textbf{Q}}_k f\Vert _{W^{s, p}} \lesssim e^{c_0\frac{\mu }{p} 2^{j\delta }} 2^{(s+\varepsilon ) k} \Vert {\textbf{Q}}_k f\Vert _{L^p_\mu } \end{aligned}$$

for any $j, k \in {\mathbb {Z}}_{\ge 0}$, where ${\textbf{Q}}_k$ is as in (2.8).

We first prove (A.1) by assuming Lemma A.1. We present the proof of Lemma A.1 at the end of this section. From (2.23) and Lemma A.1, we have

$$\begin{aligned} \Vert f \Vert _{W^{s, p}_\mu }&\le \sum _{k = 0}^\infty \Vert {\textbf{Q}}_k f \Vert _{W^{s, p}_\mu } \le \sum _{k = 0}^\infty \sum _{j = 0}^\infty e^{-\frac{\mu }{p} 2^{j\delta } } \Vert \phi _j {\textbf{Q}}_k f \Vert _{W^{s, p}}\\&\lesssim \sum _{k = 0}^\infty \sum _{j = 0}^\infty e^{-\frac{\mu }{p} 2^{j\delta } } e^{c_0\frac{\mu '}{p} 2^{j\delta }} 2^{(s+\varepsilon ) k} \Vert {\textbf{Q}}_k f \Vert _{L^p_{\mu '}}\\&\lesssim \Vert f\Vert _{B^{s', \mu '}_{p, 1}}, \end{aligned}$$

provided that $\mu > c_0 \mu '$ and $s < s'$. This proves (A.1).

Next, we prove

A.3 $$\begin{aligned} \Vert f\Vert _{B^{s, \mu }_{p, \infty }} \lesssim \Vert f \Vert _{W^{s, p}_{\mu '}} \end{aligned}$$

for any $s \in {\mathbb {R}}$ and $0< \mu ' < c_1 \mu$ for some small $c_1 > 0$.

From (2.17) and (2.18), we have

A.4 $$\begin{aligned} 2^{sk}\Vert {\textbf{Q}}_k f\Vert _{L^p_\mu } \le \sum _{j, j' = 0}^\infty 2^{sk}\Vert \phi _{j'} {\textbf{Q}}_k (\phi _j f)\Vert _{L^p_\mu } \le \sum _{j, j' = 0}^\infty e^{-c \frac{\mu }{p} 2^{j' \delta }} 2^{sk}\Vert \phi _{j'} {\textbf{Q}}_k (\phi _j f)\Vert _{L^p}.\nonumber \\ \end{aligned}$$

Note that we have

A.5 $$\begin{aligned} \big (\phi _{j'} {\textbf{Q}}_k (\phi _j f)\big )(x) = \phi _{j'}(x) \int _{{\mathbb {R}}^d} \eta _k (x-y) (\phi _j f)(y) dy, \end{aligned}$$

where $\eta _k$ is as in (2.9). In view of (2.16), we have $|x| \sim 2^{j'}$ and $|y|\sim 2^j$ in the integration above.

We first estimate the contribution to (A.4) from the case $j' \ge j - 2$. By first summing over $j'$ and applying Bernstein’s inequality and (2.23), we have

A.6 $$\begin{aligned} \begin{aligned}&\sum _{j = 0}^\infty \sum _{j' = j - 2}^\infty e^{-c \frac{\mu }{p} 2^{j' \delta }} 2^{sk}\Vert \phi _{j'} {\textbf{Q}}_k (\phi _j f)\Vert _{L^p}\\&\quad \lesssim \sum _{j = 0}^\infty e^{-c' \frac{\mu }{p} 2^{j \delta }} 2^{sk}\Vert {\textbf{Q}}_k (\phi _j f)\Vert _{L^p}\\&\quad \lesssim \sum _{j = 0}^\infty e^{-c' \frac{\mu }{p} 2^{j \delta }} e^{\frac{\mu '}{p} 2^{j\delta }} \Vert f\Vert _{W^{s, p}_{\mu '}}\\&\quad \lesssim \Vert f\Vert _{W^{s, p}_{\mu '}}, \end{aligned} \end{aligned}$$

uniformly in $k \in {\mathbb {Z}}_{\ge 0}$, provided that $0< \mu ' < c' \mu$.

Next, we consider the case $j' \le j - 3$. In this case, we have $|x - y| \sim |y|\sim 2^j$ in (A.5). Then, it follows from (2.10) that

A.7 $$\begin{aligned} |\eta _k(x-y)| \lesssim 2^{d k} e^{-c |2^k (x-y)|^\frac{1}{\theta _0}} \lesssim 2^{dk} e^{- c' |2^k \cdot 2^j |^\frac{1}{\theta _0}} e^{-c' |2^k (x-y)|^\frac{1}{\theta _0}}. \end{aligned}$$

In the following, it is understood that, given $j \in {\mathbb {Z}}_{\ge 0}$, we have $|x- y|\sim |y|\sim 2^j$ in (A.5). Hence, by first summing over $j'$ and applying Bernstein’s inequality followed by Young’s inequality with (A.7) and (2.23), we obtain

A.8 $$\begin{aligned} \begin{aligned}&\sum _{j = 0}^\infty \sum _{j' = 0}^{j-3} e^{-c \frac{\mu }{p} 2^{j' \delta }} 2^{sk}\Vert \phi _{j'}( \eta _k* (\phi _j f))\Vert _{L^p} \lesssim \sum _{j = 0}^\infty \Vert \eta _k* \langle \nabla \rangle ^s (\phi _j f)\Vert _{L^p}\\&\quad \lesssim \sum _{j = 0}^\infty e^{-c'' |2^k \cdot 2^j |^\frac{1}{\theta _0}} \Vert \phi _j f\Vert _{W^{s, p}} \lesssim \sum _{j = 0}^\infty e^{- c'' |2^k \cdot 2^j |^\frac{1}{\theta _0}} e^{\frac{\mu '}{p}2^{j\delta }} \Vert f\Vert _{W^{s, p}_{\mu '}}\\&\quad \lesssim \Vert f\Vert _{W^{s, p}_{\mu '}} \end{aligned} \end{aligned}$$

for any $\mu ' \in {\mathbb {R}}$ since we have $0< \delta < \frac{1}{\theta _0}$; see the definition (2.11) of the weight $w_\mu$.

Putting (A.4), (A.6), and (A.8) together, we obtain (A.3).

We conclude this paper by presenting the proof of Lemma A.1.

### Proof of Lemma A.1

We first consider the case $s \le 0$. From (2.23), we have

A.9 $$\begin{aligned} \Vert \phi _j {\textbf{Q}}_k f\Vert _{W^{s, p}} \le \Vert \phi _j {\textbf{Q}}_k f\Vert _{L^p} \le e^{\frac{ \mu }{p} a_1 2^{j\delta }} \Vert {\textbf{Q}}_k f \Vert _{L^p_\mu } \end{aligned}$$

for any $\mu > 0$, uniformly in $k \in {\mathbb {Z}}_{\ge 0}$. By (2.27) and Bernstein’s inequality, we have

A.10 $$\begin{aligned} \Vert \phi _j {\textbf{Q}}_k f\Vert _{W^{s, p}} \lesssim \Vert {\textbf{Q}}_k f\Vert _{W^{s, p}} \lesssim 2^{sk}\Vert {\textbf{Q}}_k f\Vert _{L^p}, \end{aligned}$$

uniformly in $k \in {\mathbb {Z}}_{\ge 0}$. Then, (A.2) follows from interpolating (A.9) and (A.10); see [8, Theorem 5.4.1] for an interpolation of weighted Lebesgue spaces.

Next, we consider the case $s > 0$. In this case, (A.2) follows from interpolating

$$\begin{aligned} \Vert \phi _j {\textbf{Q}}_k f\Vert _{L^p} \le e^{ \frac{\mu }{p} a_1 2^{j\delta }} \Vert {\textbf{Q}}_k f \Vert _{L^p_\mu } \end{aligned}$$

and (A.10) (with *s* replaced by $s + \varepsilon$). $\square$

## Footnotes

1 Namely, the ‘Hamiltonian’ stochastic quantization equation given as the Langevin equation with the momentum $v = \partial _tu$; see (1.6). The parabolic $\Phi ^{k+1}_d$-model is the stochastic gradient flow for the energy functional *E*(*u*) defined in (1.3).

2 The subscript *d* denotes the dimension of the underlying space.

3 Remark 1.5 in the arXiv version.

4 Hereafter, we may drop the inessential factor $2\pi$.

5 We use ${\mathcal {Z}}_{s, L}$, etc. to denote various normalizing constants, which may vary line by line.

6 Note that the definition (1.17) of the Wick power depends on the period $L > 0$. We, however, suppress the subscript *L* from the Wick power, when it is clear from the context.

7 Namely, $\vec {\rho }_L$ and $\vec {\mu }_L$ are mutually absolutely continuous.

8 In particular, $B_0$ is a standard real-valued Brownian motion.

9 Here, we endow the space $C({\mathbb {R}}_+; H^s({\mathbb {T}}^2_L))$ with the compact-open topology in time, namely with the topology of uniform convergence on compact intervals.

10 We say that $u \in C({\mathbb {R}}_+; \vec {H}_loc ^{s}({\mathbb {R}}^2))$ if $u \in C({\mathbb {R}}_+; \vec {H}^{s}(K))$ for each compact subset $K \subset {\mathbb {R}}^2$.

11 See also [30] which appeared after the the first version of this paper.

12 Some of the works mentioned below are on SNLW without damping.

13 As pointed out in [93], local well-posedness of of SdNLW (1.1) or SNLW (1.35) on ${\mathbb {R}}^2$ essentially requires a global control (which also yields global well-posedness) and remains open for $k \ge 5$.

14 Namely, the Wick powers of the associated linear solution.

15 In a recent preprint [4], Barashkov and Laarne independently obtained global well-posedness and invariance of the Gibbs measure the deterministic NLW (1.4) on ${\mathbb {R}}^2$ (with $k = 3$).

16 See a recent preprint [87] on the triviality of the $\Phi ^3_2$-measure on the plane.

17 Definition 2.1 in the arXiv version.

18 Proposition 2.2 in the arXiv version.

19 (3.24) in the arXiv version.

20 Lemma 2.6 in the arXiv version.

21 This means that $\langle X, \varphi \rangle \in {\mathcal {H}}_{\le k}$ for any test function $\varphi$.

22 When $\ell = k$, we have $q \le 2$ for $r > 1$ sufficiently close to 1, and thus there is no need to apply Sobolev’s inequality.

23 Theorem 5.4 in the arXiv version.

24 Lemma 5.2 in the arXiv version.

25 More precisely, recall that the product of independent homogenous Wiener chaoses is a homogenous Wiener chaos of the order given by the sum of the two.

26 Recall that the space of continuous functions from a separable metric space *X* to another separable metric space *Y* with the compact-open topology is separable; see [57]. See also the paper [50, Corollary 3.3]

27 The condition $p = p(\varepsilon ) \gg 1$ is needed in Propositions 4.2, 4.3, and 4.9.

28 As for $\xi$, we should really view $\Psi = \Psi (\xi )$ as a given space-time distribution.

29 Namely, the laws of $\{ {\mathcal {S}}(t)Z_L(1): t\in [0,T] \}_{L\ge 1}$ are tight as probability measures on $C([0, T]; W^{-\varepsilon , p}_\mu ({\mathbb {R}}^2))$.

30 Strictly speaking, in applying Lemma 2.13, we need to go back to the frequency truncated version of the Wick power, just as in (1.25) for the damped wave equation, and remove the frequency truncation.

31 We assume that $t + h \ge 0$.

32 Theorem 5.4 in the arXiv version.

33 Theorem 5.4 in the arXiv version.

34 Theorem 5.4 in the arXiv version.

35 Proposition 3.11 in the arXiv version.

36 Theorem 5.4 in the arXiv version.

37 Theorems 8.1 and 9.5 in the arXiv version.

38 It suffices to take $\delta ^* \le C(\mu , R) \delta _*$ for some $C(\mu , R) > 0$.
